# Phytochemistry and Biological Activities of Iris Species Growing in Iraqi Kurdistan and Phenolic Constituents of the Traditional Plant *Iris postii*

**DOI:** 10.3390/molecules26020264

**Published:** 2021-01-07

**Authors:** Hawraz Ibrahim M. Amin, Faiq H. S. Hussain, Soran K. Najmaldin, Zaw Min Thu, Mohammed Farhad Ibrahim, Gianluca Gilardoni, Giovanni Vidari

**Affiliations:** 1 Dipartimento di Scienze del Farmaco, Università del Piemonte Orientale, Largo Donegani 2, 28100 Novara, Italy; 2 Department of Chemistry, College of Science, Salahaddin University-Erbil, Erbil 44001, Kurdistan Region, Iraq; 3 Medical Analysis Department, Faculty of Science, Tishk International University, Erbil 44001, Kurdistan Region, Iraq; faiq.hussain@tiu.edu.iq; 4 Biology Department, Faculty of Education, Tishk International University, Erbil 44001, Kurdistan Region, Iraq; soran.kayfi@tiu.edu.iq; 5 Department of Chemistry, Kalay University, Kalay 03044, Myanmar; zawminthu87@gmail.com; 6 Department of Drug Science, University of Pavia, Viale Taramelli 10, 27100 Pavia, Italy; gardy1988@gmail.com; 7 Departamento de Química y Ciencias Exactas, Universidad Técnica Particular de Loja, Calle Marcelino Champagnat s/n, Loja 110107, Ecuador; ggilardoni@utpl.edu.ec; 8 Dipartimento di Chimica, Università di Pavia, Via Taramelli 12, 27100 Pavia, Italy

**Keywords:** phenolics, terpenoids, isoflavones, swertisin 6-*C*-glycosides, resveratrol 3,4′-*O*-diglucoside, *Iris postii*, antioxidant activity, Kurdish medicinal plants

## Abstract

A dozen *Iris* species (Iridaceae) are considered traditional remedies in Kurdistan, especially for treating inflammations. Phytochemical studies are still scarce. The information reported in the literature about *Iris* species growing in Kurdistan has been summarized in the first part of this paper, although, except for *Iris persica*, investigations have been performed on vegetal samples collected in countries different from Kurdistan. In the second part of the work, we have investigated, for the first time, the contents of the methanolic extracts of *Iris postii* aerial parts and rhizomes that were collected in Kurdistan. Both extracts exhibited a significant dose-dependent free radical scavenging and total antioxidant activities, comparable to those of ascorbic acid. Medium-pressure liquid chromatographic separations of the two extracts afforded l-tryptophan, androsin, isovitexin, swertisin, and 2″-*O*-α-l-rhamnopyranosyl swertisin from the aerial parts, whereas ε-viniferin, *trans*-resveratrol 3,4′-*O*-di-β-d-glucopyranoside, and isotectorigenin were isolated from the rhizomes. This is the first finding of the last three metabolites from an *Iris* species. The various remarkable biological activities of isolated compounds scientifically sustain the traditional use of *I. postii* as a medicinal plant.

## 1. Introduction

Traditional medicines still hold an important role among health care practices of many countries, including Arab countries and Iraqi Kurdistan [1]. A Neanderthal burial discovered at Shanidar cave (number IV in the series of skeletons) in northern Iraq, dated approximately 60,000 years ago [2], is evidence that herbal medicine has probably been practiced in the mountains and plains of Kurdistan since the dawn of civilization. Indeed, a well-organized form of medicine, which made intense uses of plant-derived drugs, remedies, potions and oils, can be traced back in Iraq to the Sumerian period (3000–1970 B.C.) and then to the Babylonian and Assyrian periods (1970–539 B.C). Later, this knowledge was translated and enriched by Arab physicians during the Abbasid period (500–1038 A.D). Still today, the majority of the approximately 1500 plants used in Iraq are appreciated for their medicinal and aromatic properties. Most medicinal plants are collected from their wild habitats, but some are also cultivated [3]. 

**Figure 1:**
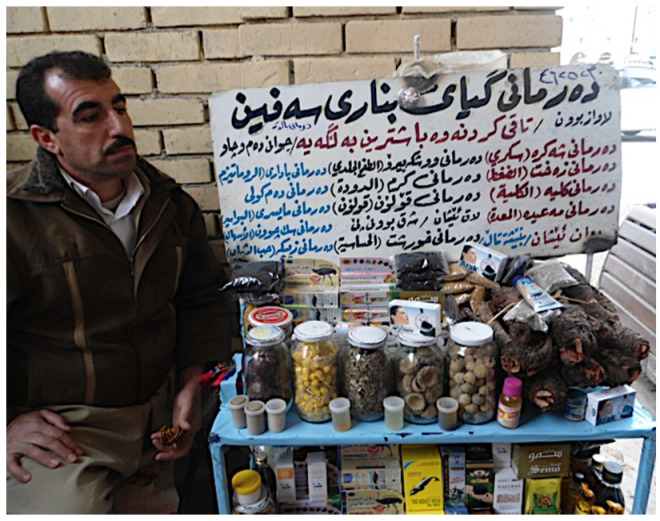
A Kurdish seller of traditional remedies (photo taken by H.I.M.A.).

Medicinal herbs growing in Iraqi Kurdistan are especially used by people living in the villages on the mountains and in the rural areas; however, sellers of natural medicinal products (Figure 1) are also present in the bazaars of the main towns, such as Erbil and Sulaymaniya [4,5].

Despite the wide use of herbal remedies, phytochemical studies on Kurdistan medicinal plants are still in their infancy and only a few papers have been published so far that describe the structures and bioactivities of isolated metabolites. As part of our ongoing project on scientific validation of Kurdistan traditional plants, we directed our attention toward the genus *Iris*. This large genus of the family Iridaceae (Angiosperms) contains about 260–300 species [6,7] of perennial plants growing from creeping rhizomes (rhizomatous irises) or, in drier climates, from bulbs (bulbous irises). The showy flowers are characterized by a violet-like scent. The plants grow in temperate regions across the Northern Hemisphere, from Eurasia to North America [8]. Many *Iris* species are ornamental plants; however, they are also used in various traditional medicines for the treatment of inflammations, cancer, bacterial and viral infections, and other diseases. Extensive phytochemical investigations of the genus have led to the isolation of different isoprenoids, flavonoids, isoflavonoids and their glycosides, xanthones, quinones, and stilbene glycosides, among others [8,9]. On the other hand, isolated bioactive compounds have shown antibacterial, anti-neoplastic, antioxidant, cytotoxic, anti-plasmodial, molluscicidal, anti-inflammatory, phytoestrogenic and antituberculosis properties [9]. Moreover, an essence called “orris butter” and an absolute essential oil with the scent of the flowers are derived from the bulbs of some *Iris*, e.g., *I. florentina* and *I. germanica*; they are used in the manufacture of luxury expensive perfumes, such as Chanel *No. 19* (1970) and *So pretty* by Cartier (1995) [10].

Twelve species of *Iris* are reported to grow in Iraq; in the Kurdistan region, they occur especially on mountainous regions, such as Halgurd Mountain (Choman) and Korek Mountain (Rawanduz). These plants include *Iris aucheri* (Baker) Sealy, *I. barnumiae* Baker et Foster, *I. caucasica* Hoffm., *I. gatesii* Foster, *I. germanica* L., *I. heylandiana* Boiss. et Reut. ex Boiss., *I. hymenospatha* B. Mathew et Wendelbo, *I. masia* Dykes, *I. persica* L., *I. postii* Mouterde, *I. pseudocaucasica* Grossh., and *I. reticulata* M. Bieb. [11]. Of these species only *I. germanica* L. and *I. persica* L. have been investigated phytochemically. *I. persica* has been investigated by a Kurdish research group [12,13]; instead, due to the wide geographical distribution and economic importance, *I. germanica* has been subjected to several investigations in countries different from Kurdistan. The results of these investigations have been summarized in the first part of this paper. The phytochemical literature reported in Scifinder and Google Scholar databases up to August 2020 has been reviewed. In the second part of this paper, we describe the results of our phytochemical investigation of non-volatile secondary metabolites isolated from the aerial parts and rhizomes of *Iris postii* Mouterdi. 

## 2. Results and Discussion

### 2.1. Literature Data about Iris germanica and Iris persica

#### 2.1.1. *Iris germanica*

*Iris germanica* L. is probably the most thoroughly investigated *Iris* species. Rhizomes have been traditionally used for various oral and topical applications, e.g., sores, freckles [14], and to relieve teething-associated pain [15]. Root decoctions of the plant have been commonly applied as antispasmodic, emmenagogue, diuretic, anti-insomnia, and cathartic agents [16]. They decrease smooth muscle activity in vivo and show anti-serotonin effects [17]. Extracts of *I. germanica* showed cytotoxic [18,19], antioxidant [20,21,22], antimutagenic [22], antifungal [23], antimicrobial [24,25,26], anti-inflammatory [24,27], anti-biofilm [28], antiulcer [29], hypolipidemic [30], molluscicidal [31], and amyloid β (Aβ) induced memory impairment activities [32]. The application of rhizomes in both traditional and modern medicine has been mainly based on the presence of isoflavones and essential oils in the extracts.

##### Isoflavonoids

**Figure 2:**
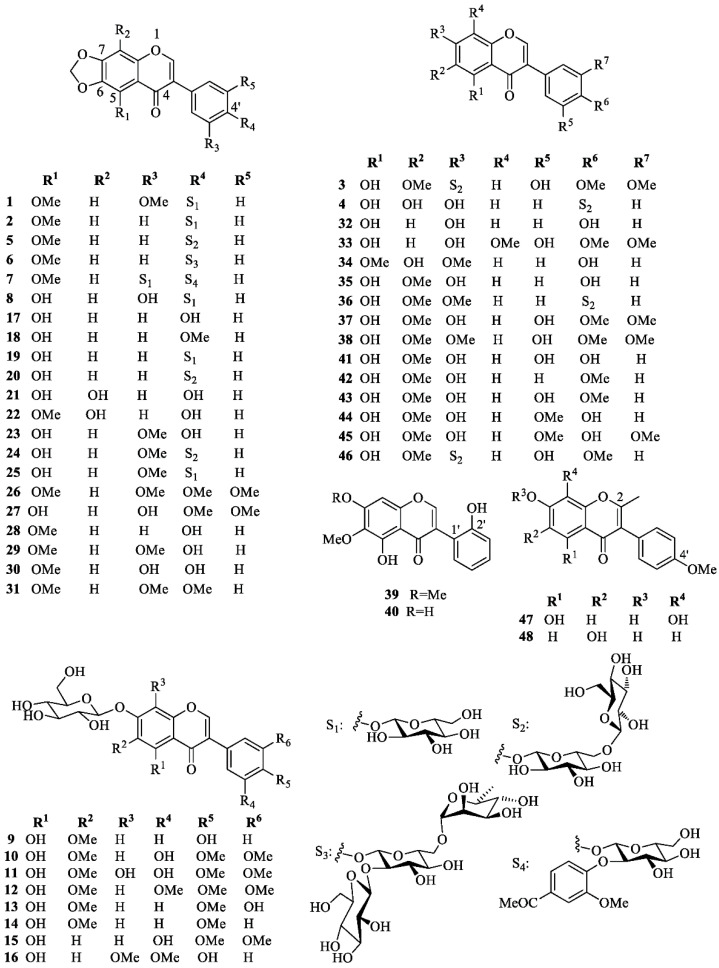
Characteristic isoflavonoids isolated from *Iris germanica*.

Isoflavonoids are mainly accumulate in the rhizomes and form the largest group of flavonoids isolated from *I. germanica*. Their structures (**1**–**48**) are shown in Figure 2, whereas the reported biological activities are shown in Table 1.

Iris isoflavonoids include germanaism A (**1**) [15,33,34], B (**2**) [15,17,33,34], C (**3**) [17], D (**4**) [17], E (**5**) [15,17], F (**6**) [17], G (**7**) [17], and H (**8**) [34], tectoridin (**9**) [35,36], iridin (**10**) [15,24,33,35,37,38,39,40], iridin A (**11**) [40], and S (**12**) [38,40,41], iristectorin A (**13**) [39], irisolidone-7-*O*-β-d-glucopyranoside (**14**) [15,37,42], 5,3′-dihydroxy-4′,5′-dimethoxyisoflavone 7-*O*-glucoside (**15**) [37], homotectoridin (**16**) [36], irilone (**17**) [14,15,24,34,38,40,42,43,44,45], irilone 4′-methyl ether (**18**) [14,25,38,40], irilone 4′-*O*-β-d-glucopyranoside (**19**) [15,24,33,34,38,40], irilone 4′-*O*-[β-d-glucopyranosyl (1→6)β-d-glucopyranoside] (**20**) [15], 8-hydroxyirilone (**21**) [38], 8-hydroxyirilone 5-methyl ether (**22**) [38], iriflogenin (**23**) [15,34,42,44], iriflogenin 4′-*O*-[β-d-glucopyranosyl-(1→6)-β-d-glucopyranoside] (**24**) [15], irifloside (**25**) [15,34], irisflorentin (**26**) [15,44,45], dichotomitin (**27**) [15,37], irisolone (nigricin) (**28**) [14,15,23,34,44,45,46], nigricanin (iriskashmirianin) (**29**) [42,44], iriskashmirianin A (**30**) [34], 3′-*O*-metyliriskumaonin (iriskumaonin methyl ether) (**31**) [15,34,44,45], genistein (**32**) [35], 5,7-dihydroxy-3-(3′-hydroxy-4′,5′-dimethoxy)-8-methoxy-4*H*-1-benzopyran-4-one (**33**) [14], muningin (**34**) [35], tectorigenin (**35**) [14,35], 7-*O*-methyl-tectorigenin-4′-*O*-[β-d-glucopyranosyl-(1→6)-β-d-glucopyranoside] (**36**) [15], irigenin (**37**) [14,15,24,25,35,37,38,39,40,42,44,45], irigenin S (**38**) [24,38,40], irilin A (**39**) [31], B (**40**) [31], and D (**41**) [35], irisolidone (**42**) [15,23,24,37,38,40,42,43,44,45,46], iristectorigenin A (**43**) [14,39,44,45], 5,7-dihydroxy-3-(3′-methoxy-4′-hydroxyphenyl)-6-methoxy-4*H*-1-benzopyran-4-one (**44**) [14,45], iristectorigenin B (**45**) [35], 5,7,3′-trihydroxy-6,4′-dimethoxyisoflavone-7-*O*-[β-d-glucopyranosyl-(1→6)-β-d-glucopyranoside] (**46**) [15], 5,7,8-trihydroxy-3-(4-methoxyphenyl)-2-methyl-4*H*-chromen-4-one (**47**) [47] and 6,7,-dihydroxy-3-(4-methoxyphenyl)-2-methyl-4*H*-chromen-4-one (**48**) [47]. *Iris* isoflavanoid structures can be collected in five different groups, depending on whether they contain: (A) a methylenedioxy group at C-6 and C-7 and an oxygenated group at C-4′; (B) an OH group at C-5 and a d-glucopyranosyl unit at 7-O; (C) different oxygenated functions but neither a methylenedioxy group nor a 7-*O*-d-glucopyranosyloxy unit; (D) a 2′-OH-group; (E) a methyl group attached to C-2. Group A includes 4′-*O*- mono-, di-, and triglycosides and one 3′-*O*-monoglucoside; group B contains only 7-*O*-monoglucosides; group C contains only 7-*O*- or 4′-*O*-diglycosides, while no sugar residue occurs in isoflavonoids of groups D and E. d-Glucopyranose is the only sugar present in mono- and diglucosides, whereas the only known triglycoside (**6**) contains an additional l-rhamnopyranosyl moiety. Diglucosides are β-d-glucopyranosyl-(1→6)-β-d-glucopyranosyl derivatives, whereas triglycoside **6** is characterized by a β-d-glucopyranosyl-(1→2)-rutinosyl unit. 

##### Other Flavonoids

**Figure 3:**
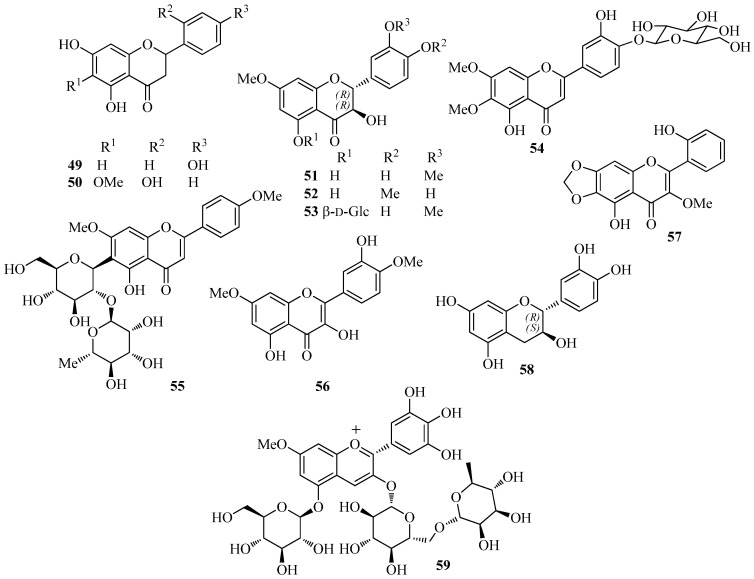
Characteristic flavonoids other than isoflavones isolated from *Iris germanica*.

Compared to isoflavonoids, representative compounds of other flavonoid classes occur less frequently in the extracts of *I. germanica*. Isolated flavanones (Figure 3) are naringenin (**49**) [35] and 5,7,2′-trihydroxy-6-methoxyflavanone (**50**) [31], whereas flavanonols include dihydroquercetin-7,3′-dimethylether (**51**) [45], dihydroquercetin-7,4′-dimethylether (**52**) [35], and dihydroquercetin-7,3′-dimethylether-5-*O*-β-d-glucopyranoside (**53**) [35]. Flavones are represented by the *O*-glucoside cirsiliol-4′-*O*-glucopyranoside (**54**) [35] and the *C*-diglucoside PID (**55**) [52,53]. Flavonols and flavan-3-ols include ombuin (**56**) [35] and 5,2′-dihydroxy-3-methoxy-6,7-methylenedioxyflavone (**57**) [31], and (+)-(2*R*,3*S*)-catechin (**58**) [22], respectively. The anthocyanin delphanin (delphinidin-3-(4-*p*-coumaroylrhamnosyl-(1→6)-glucoside)-5-glucoside) (**59**) was isolated from flowers of *I. germanica* [54].

##### Miscellaneous Aromatic Compounds 

**Figure 4:**
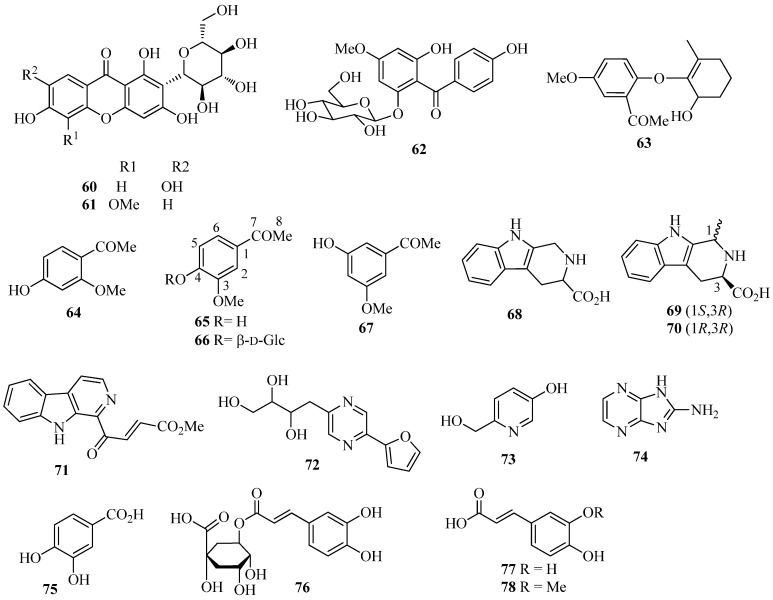
Miscellaneous aromatic compounds isolated from *Iris germanica*.

Other aromatic compounds (Figure 4) isolated from *I. germanica* include the *C*-glucosylxanthones mangiferin (**60**) [35] and irisxanthone (**61**) [35]; the phenol derivatives 2,4′,6-trihydroxy-4-methoxybenzophenone-2-*O*-β-d-glucoside (**62**) [35], 1-(2-(6′-hydroxy-2′-methylcyclohex-1′-enyloxy)-5-methoxyphenyl)ethanone (**63**) [23,44], isopeonol (**64**) [43], acetovanillone (**65**) [15,23,35,37,44,46], androsin (**66**) [35], and 3-hydroxy-5-methoxyacetophenone (**67**) [24]; the alkaloids 1,2,3,4-tetrahydro-β-carboline-3-carboxylic acid (**68**) [55] and the corresponding (*S*)-(–)-methyl ester [55], (1*S*,3*R*)-methyl 1-methyl-2,3,4,9-tetrahydro-1*H*-pyrido[3,4-b]indole-3-carboxylate (**69**) [55], methyl (1*R*,3*R*)-1-methyl-2,3,4,9-tetrahydro-1*H*-pyrido[3,4-b]indole-3-carboxylate (**70**) [55], 4-(9*H*-β-carbolin-1-yl)-4-oxobut-2-enoic acid methyl ester (**71**) [55], 2-(furan-2-yl)-5-(2,3,4-trihydroxybutyl)-1,4-diazine (crotonine) (**72**) [55], 3-β-d-ribofuranosyluracil (uridine) [55], 6-hydroxymethyl-3-pyridinol (**73**) [55], and 2-amino-1*H*-imidazo-[4,5-*b*]pyrazine (**74**) [55]; the benzoic and cinnamic acid derivatives pyroglutamic acid [35], protocatechuic acid (**75**) [22], chlorogenic acid (**76**) [22], caffeic acid (**77**) [22], ferulic acid (**78**) [22], and *p*-hydroxy benzoic acid [22]. Crotonine (**72**) showed good analgesic activity in vivo [55].

##### Terpenoids

**Figure 5:**
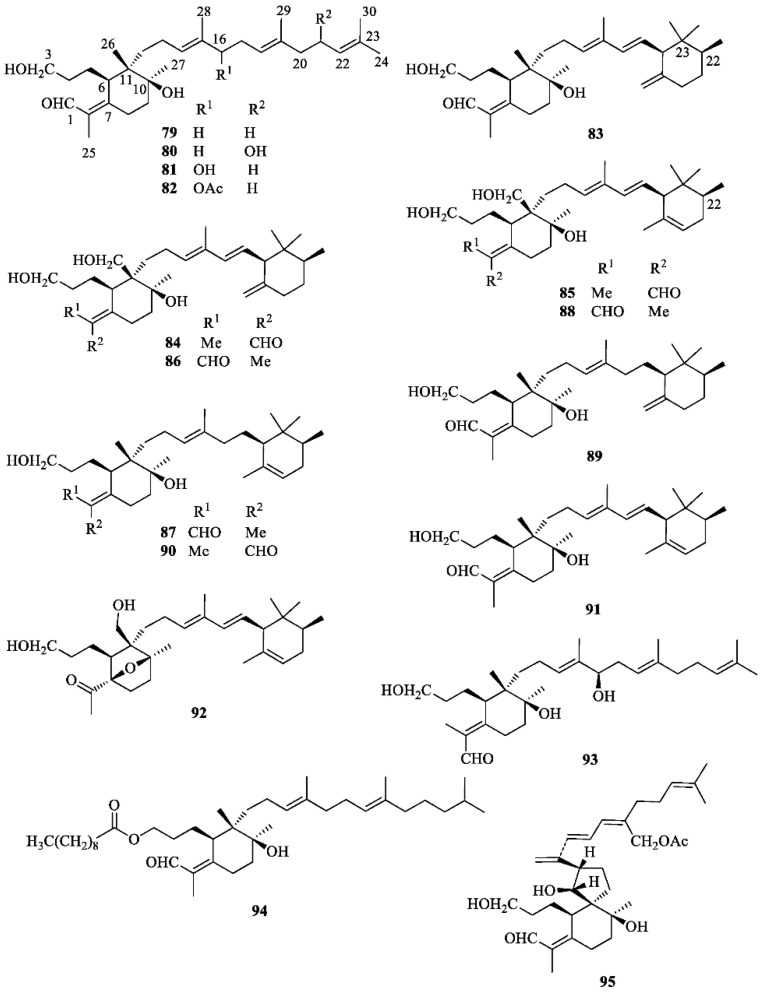
Iridals isolated from *Iris germanica*.

In addition to α- and β-amyrin [37], the most widespread triterpenoids occurring in *I. germanica* are iridals (Figure 5) [56]. These bitter tasting terpenoids can be isolated in appreciable amounts from the unsaponifiable fraction of lipid extracts from the rhizomes. Characteristic features of all iridals are a multi-substituted cyclohexane ring with a long side chain at C-11 (squalene numbering), an acrolein group at C-7, and a hydroxypropyl chain at C-6. The latter two substitutions are typical fragments of a seco A-ring of triterpenoids. Appropriate labeling experiments have shown that 2,3-epoxysqualene is the precursor of the iridals and that a bicyclic intermediate is possibly formed in the biosynthetic pathway, the A ring of which is subsequently opened to give the iridal skeleton [56]. Other labeling experiments have also proved the involvement of activated methionine for the introduction of the extra methyl group at C-22 of an open-chain precursor of methylated cycloiridals and irones [56]. The large group of iridals isolated from *I. germanica* include iridal (**79**) [57,58,59,60], iridogermanal (**80**) [61], isoiridogermanal (**81**) [60,62,63], 16-*O*-acetylisoiridogermanal (**82**) [59,60,62], irisgermanical A (**83**) [60,62], irisgermanical B (**84**) [60,62], irisgermanical C (**85**) [60,62,63], iriflorental (**86**) [59,60,62,63], α-irigermanal (**87**) [59,60,61,62,63], iripallidal (**88**) [60,62,63], γ-irigermanal (**89**) [29,59,60,61,62,63], compound **90** [63], α-dehydroirigermanal (**91**) [60,62,63], irigermanone (**92**) [63], iridobelamal A (**93**) [63], iristectorone K (**94**) [64], 29-acetoxyspiroiridal (**95**) [59]. Compound **79** showed potent antimalarial activity both in vitro and in vivo [57]. Compounds **79**, **82**, **86**, **87**, **89**, and **95** exhibited significant cytotoxicity against K562 leukema and A2780 ovarian cell lines [59]. Compounds **86**, **88** and **89** showed potent piscicidal activities at a concentration of less than 1 μg/mL of median tolerance limit (TLm) value [60,62], and compound **89** exhibited significant antiulcer activity [29,65].

**Figure 6:**
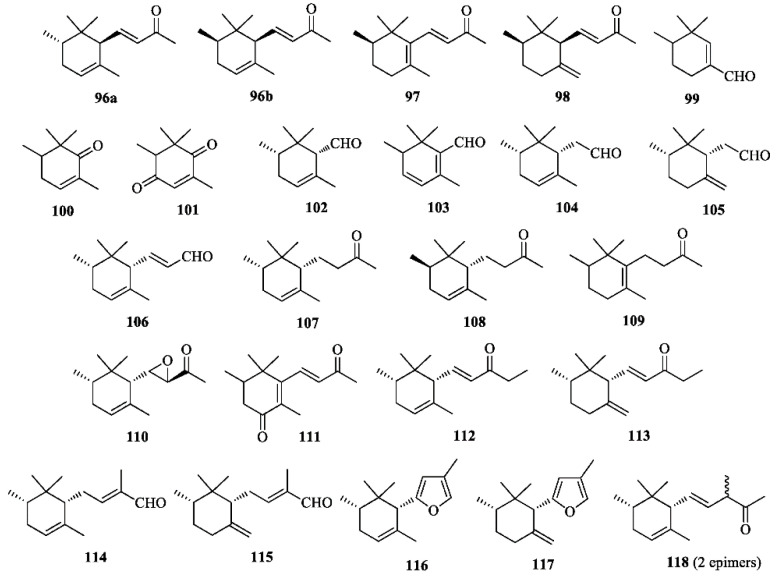
Irones and irone-related compounds isolated from *Iris germanica*.

α- (**96**), β- (**97**), and γ-Irone (**98**) (Figure 6) are the odoriferous principle of iris oils. It is well known that freshly harvested iris rhizomes do not contain irones, but their triterpenoid precursors iridals (Figure 5). According to the traditional procedure, decorticated rhizomes of some *Iris* species (e.g., *I. germanica*, *I. pallid*a, *I. florentina*) are kept in a dry and aerated environment for 2–3 years, then powdered, incubated with diluted sulphuric acid, and steam-distilled to provide the precious “orris butter”. The mechanism of the oxidative degradation affording irones from iridals is still poorly understood. The traditional process is long, troublesome and low yielding; hence, the high cost of the essence (butter). Purification of the essence eliminates the fatty acids and yields the absolute, which is sold at several thousands of dollars per kilogram [10]. It has been established that the distribution of irone isomers and enantiomers in different qualities of iris oils depends upon the botanical species of the plant [44]. Thus, the average composition of an iris butter prepared from *Iris germanica* is the following: 0.91% of (+)-*trans*-α-irone (ee = 96%, (+)-**96a**), 61.48% of (−)-*cis*-α-irone (ee = 82%, (−)-**96b**), 0.71% of β-irone (**97**) and 37.60% of (−)-*cis*-γ-irone (ee = 38%, (−)-**98**) [10]. Irone-related compounds **99**–**118** (Figure 6) are also occurring in the essential oil [66].

##### Steroids and Miscellaneous Compounds

**Figure 7:**
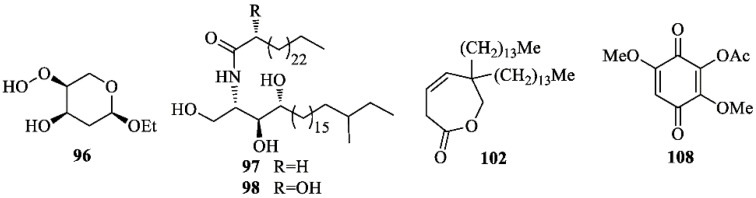
Miscellaneous compounds isolated from *Iris germanica*.

Common sterols isolated from *I. germanica* include: cholesterol [67], campesterol [67], sitosterol [37,67], β-sitosterol [35], daucosterol [35], stigmasterol [24,67], and stigmasterol-3-*O*-β-d-glucopyranoside [24]. Compounds of different biogenetic origin (Figure 7) include iriside A (**96**) [24], irisamides A (**97**) [41], irisamides B (**98**) [41], 6,6-ditetradecyl-6,7-dihydrooxepin-2(3*H*)-one (**102**) [23], 2-acetoxy-3,6-dimethoxy-1,4-benzoquinone (**108**) [23]. Compounds **97** and **98** were reported to be active against L5178Y and Hela tumor cell lines [41].

#### 2.1.2. *Iris persica*

*I. persica* is used to treat tumors and wound inflammation in the traditional medicine of Kurdistan [5]. Essential oils obtained by hydrodistillation of air-dried flowers, leaves, rhizomes, and fresh bulbs were investigated by GC–FID and GC–MS; moreover, the oil antifungal activities were determined [12]. The major constituents of the flower essential oil were phenylethanol (24.8%) and furfural (13.8%). This aldehyde was also the main component of the leaf and rhizome volatile fractions, with percentages of 39.0% and 22.2%, respectively. Phenylacetaldehyde (37.1%) was the main constituent of the volatile fraction from the bulbs. The oils exhibited moderate antifungal activity in vitro against strains of the human pathogenic fungi *Candida albicans*, *Microsporum canis*, and *Trichophyton mentagrophytes*, the plant–fungal pathogen *Pyricularia oryzae*, and the fungal food contaminant *Aspergillus carbonarius*. The highest activity was exhibited by the essential oils isolated from leaves and flowers, so that they could be considered natural antimicrobial agents.

**Figure 8:**
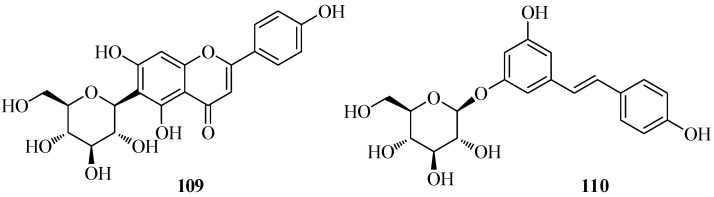
Characteristic phenolic compounds isolated from *Iris persica*.

A few known bioactive flavonoids (Figure 8) were abundant in the non-volatile extracts of the plant. Thus, the *C*-diglucoside flavone embinin (**55**) was isolated from flowers and leaves, the 6-*C*-glucoside flavone isovitexin (**109**) was isolated from flowers, the stilbenoid (−)-*trans*-resveratrol-3-*O*-β-d-glucopyranoside (**110**) was found in rhizomes, and the isoflavone (+)-tectorigenin (**35**) was isolated from bulbs [13]. In an MTT assay, embinin (**55**) showed a significant inhibitory activity that was higher than the well-known antitumor drug cisplatin, against MCF7, SkBr3, Ishikawa, BG-1, and A549 human tumor cells. Moreover, embinin showed a remarkable DPPH radical scavenging activity, that was comparable to that of the well-known antioxidant ascorbic acid [13].

### 2.2. Phytochemical Studies on Iris postii

**Figure 9:**
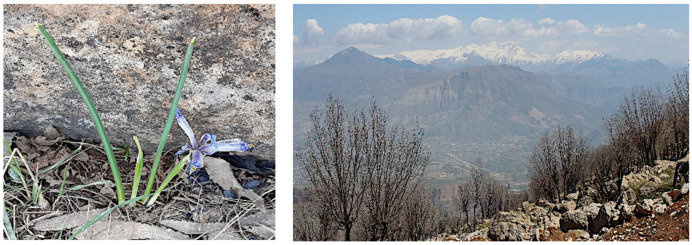
*Iris postii* and Korek mountain where the plant was collected (photos taken by H.I.M.A.).

A decoction of the aerial parts of *Iris postii* Mouterde is used in the Iraqi folkloric medicine as a general remedy against inflammations. The plant, which is native to Middle East, grows wildly on the slopes of Mount Korek (Figure 9), a mountain located in the Erbil province not far from the Iranian border, where it was collected for this investigation.

Neither phytochemical investigations nor evaluations of biological activities have been carried out on extracts of *I. postii* so far. Therefore, on the assumption that the bioactivity mostly resided in polar metabolites, we decided to examine the phytochemical contents and the antioxidant properties of polar extracts of the aerial parts and rhizomes.

**Figure 10:**
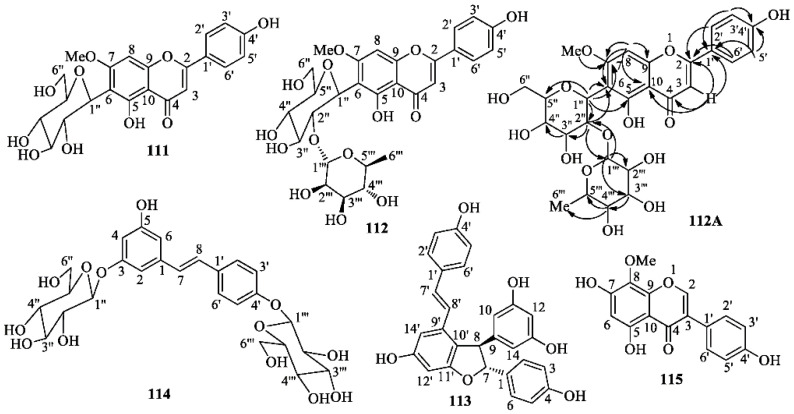
Structures of swertisin (**111**), 2″-*O*-rhamnosyl swertisin (**112**), viniferin (**113**), resveratrol 3,4′-*O*-di-β-d-glucopyranoside (**114**), isotectorigenin (**115**) isolated from *Iris postii* Mouterde; H→C HMBC correlations determined in the 2D-NMR spectrum of 2″-*O*-rhamnosyl swertisin (**112A**).

At first, powdered aerial parts and rhizomes were separately defatted by soaking in hexane at room temperature; most chlorophyll was also removed in this manner. Successively, each biomass was extracted with MeOH. The yields of the residues, IPA from the aerial parts and IPR from the rhizomes, were 1.3 and 2.75% (*w*/*w*), respectively. Successively, IPA and IPR were separately partitioned between H_2_O and dichloromethane and H_2_O and *n*-butanol, respectively, to give fractions IPAD and IPRB, respectively. Multiple medium-pressure liquid chromatographic separations of a sample of IPAD on reversed-phase (RP-18) columns afforded L-tryptophan, androsin (**66**), apigenin 6-C-glucoside (isovitexin) (**109**), swertisin (**111**), and 2″-*O*-rhamnosyl swertisin (**112**). Analogous chromatographic separations of a sample of IPRB gave *trans*-ε-viniferin (**113**), *trans*-resveratrol 3,4′-*O*-diglucoside (**114**), and isotectorigenin (**115**). The structures of isolated compounds (Figure 10) were established mainly by extensive 1D- and 2D-NMR experiments and MS spectrometry. Comparing our spectroscopic data with the pertinent literature, we found some differences between our data and those reported from different laboratories, especially for the NMR signals of swertisin (**111**) [68,69,70,71,72,73] and 2″-*O*-rhamnosylswertisin (**112**) [71,74]; moreover, literature data are often not consistent between each other and some spectra have been recorded in solvents different from those used in this work. Therefore, although the isolated compounds are known, the physical and spectroscopic data determined by us are reported in the Experimental section, whereas the graphics are included in the Supplementary Materials.

It is now generally accepted by scientists that excess oxidants and radicals, especially oxygen radicals, through damage and mutation of DNA and other biomolecules, play a major role in degenerative processes that may cause the insurgence and progression of inflammatory processes, cancer, cardiovascular and atherosclerotic diseases, neurodegeneration, and aging [75,76]. Antioxidants isolated from natural sources could thus become important chemotherapeutic agents in defense mechanisms against these toxic agents. Therefore, with the aim to give some scientific evidence to the traditional use of *I. postii* in the treatment of inflammations and in search of a new source of natural antioxidants, two simple tests were performed in vitro to determine the total antioxidant capacity (TAOC) of the crude extracts (see text) and the antiradical activity of the extracts and the isolated compounds. The TAOC values of the extracts were determined by the phosphomolybdate method (adjusted from references [77,78,79]), using ascorbic acid as the standard. The assay was based on the reduction of hexavalent molybdenum Mo (VI) to the pentavalent form [Mo (V)] by an antioxidant, and the formation of a green phosphate/Mo (V) complex at acidic pH and at high temperature. The TAOC values were expressed as µg ascorbic acid equivalent/mg extract (Table 2). The greater this value, the higher was the antioxidant capacity. Thus, the total methanol extract of the aerial parts (IPA) and the *n*-butanol sub-extract of the methanolic extract of the rhizomes (IPRB) exhibited the highest total antioxidant activity. Moreover, comparing the TAOC of the IPA extract with that of the dichloromethane sub-extract (IPAD), it appears that highly antioxidant compounds, likely very polar, were not adequately extracted by CH_2_Cl_2_. Therefore, they need further study.

Subsequently, the free radical scavenging (FRS) activity of the isolated compounds **66**, **109**, **111**–**115**, the crude extracts and the standard ascorbic acid were determined using the 2,2-diphenyl-1-picrylhydrazyl radical (DPPH) method (adjusted from references [78,79]). DPPH is a stable, nitrogen-centered free radical which produces violet/purple color in methanol solution and fades to shades of yellow color in the presence of a hydrogen radical/electron-donor compound. The antiradical activity was expressed as EC_50_ value, i.e., the concentration (μg/mL) of the sample required to scavenge 50% of the initial DPPH concentration and as μg ascorbic acid equivalents/μg sample. In the case of isolated compounds, the activity was also measured as EC_50_ (μM/L), which, in our opinion, is a more accurate measurement of the intrinsic antiradical activity of a compound. The results of the DPPH assay (Table 2) essentially confirmed those obtained by the molybdate test as far as the antioxidant activity of the extracts is concerned. On the other hand, concerning the DPPH scavenging activity of single compounds, 2″-*O*-α-l-rhamnosyl- swertisin (**112**), ε-viniferin (**113**), and resveratrol 3,4′-*O*-di-β-d-glucopyranoside (**114**) were as effective as the standard ascorbic acid or even more efficacious. The remaining isolated compounds, androsin (**66**), isovitexin (**109**), swertisin (**111**), and isotectorigenin (**115**), were moderately active, although the EC50 values of compounds **109**, **111** and **115**, expressed as μM/L, were lower than that of ascorbic acid.

## 3. Material and Methods

### 3.1. General Experimental Techniques and Procedures

For most general experimental techniques and procedures, see reference [80]; ^1^H-NMR and ^13^C-NMR chemical shifts (δ, ppm) are relative to signals of residual C*H*D_2_OD in CD_3_OD δ_H_ 3.27 (central line of a quintuplet), ^13^*C*D_3_OD [*δ*_C_ 49.0 (central line of a septuplet)], and ^13^C-4 of C_5_D_5_N [δ_C_ 134.3 (central line of a triplet)], respectively. All the NMR experiments were performed on a Bruker AV300 spectrometer, at 300 (^1^H) and 75.47 MHz (^13^C), respectively. Deuterated solvents (purity 99.8%) were purchased from Sigma-Aldrich (St. Louis, MO, USA). ESI-MS experiments were carried out on a Thermo-TSQ mass spectrometer, by flow injection analysis (FIA), with the electron-spray ionization source (ESI) at 5 kV on TIP capillary. Spectroscopy grade solvents (Sigma-Aldrich) were used. Preparative medium-pressure liquid chromatographic (MPLC) separations were carried out on a Biotage Isolera instrument (Biotage, Uppsala, Sweden).

### 3.2. Plant Material

Aerial parts and rhizomes of *I. postii* Mouterde were separately collected on Korek Mountain (GPS position: 36°35′20″ N, 44°27′32″ E). The plant was identified by botanist A. H. Al-khayyat of Salahaddin University-Erbil/Iraq. A voucher specimen (accession number 7230) has been deposited at the Education Salahaddin University Herbarium (ESUH). The vegetal materials were cleaned and air-dried under shade at room temperature (20–25 °C) in a ventilated room until they reached constant weight. After drying, each plant part was finely powdered using a laboratory grinding mill, and powdered materials were stored in bottles at room temperature until analyses.

### 3.3. Extraction of I. postii and Chromatographic Fractionation of Extracts

Powdered aerial parts and rhizomes (200 g each) were separately soaked in hexane (800 mL) with occasional shaking in an ultrasonic bath for 20 min, then left in the same solvent for 5 h under continuous stirring at room temperature. Subsequently, the mixture was decanted and filtered. This procedure was repeated three times for each part. Defatted rhizomes and aerial parts were subsequently separately suspended in MeOH (800 mL) n an ultrasonic bath for 20 min and then left in the same solvent for 5 h under continuous stirring, at room temperature. The procedure was repeated three times for each vegetable part. The mixtures were then filtered, and the solvent removed under vacuum in a rotary evaporator to afford two crude residues: IPA (2.6 g) from aerial parts and IPR (5.5 g) from rhizomes.

Subsequently, IPA (2.5 g) was suspended in MeOH-H_2_O (75:25, 350 mL) and extracted with CH_2_Cl_2_ (3 × 350 mL) to afford, upon evaporation, a dichloromethane soluble powder (1.9 g) (IPAD). Then, 500 mg of this residue was separated on a hand-packed reversed phase column (LiChroprep RP18, 25–40 μm, 120 g, MerckMillipore, Darmstadt, Germany) using a medium pressure liquid chromatographic (MPLC) instrument (Isolera ONE, Biotage, Uppsala, Sweden). Solvents A and B of the mobile phase were H_2_O and MeOH, respectively. A linear gradient was applied from a 70:30 A/B mixture to 100% solvent B (MeOH), over 30 min at room temperature, at a flow rate of 30 mL/min; the detection UV wavelength was set at UV 254–366 nm. Finally, the column was washed with 100% MeOH for 3 min to elute strongly adsorbed compounds. Forty-five fractions (20 mL each) were collected; the solvent in the tubes was evaporated using a centrifuge under vacuum and a liquid nitrogen trap, and the residues were weighed. The overall recovery of the chromatographed mixture was 95%. Then, the content of each fraction was analyzed by TLC on analytical silica gel 60 (GF_254_, Merck, Darmstadt, Germany) plates, eluted with EtOAc/*n*-BuOH/HCO_2_H/H_2_O (5:3:1:1), and on RP-18 (Sigma-Aldrich) plates, eluted with MeOH/H_2_O (1:1). Spots were detected under UV light at 254 and 366 nm and by spraying the plates with 0.5% vanillin in sulfuric acid/EtOH (4:1), followed by heating at 105 °C for about 1 min. Repeated MPLC separation of main fractions on reversed-phase columns afforded L-tryptophan (29 mg), androsin (**66**, 13 mg), apigenin 6-*C*-glucoside (isovitexin) (**109**, 45 mg), swertisin (**111**, 17 mg), 2″-*O*-rhamnosyl swertisin (**112**, 12 mg). A portion of IPR (5 g) was suspended in 500 mL of water and partitioned with 500 mL of *n*-butanol to yield, after evaporation, 2.2 g of residue IPRB. Then, 1 g of this mixture was loaded onto a preparative RP-MPLC column installed in the MPLC instrument that was eluted with a gradient of 20–100% MeOH in H_2_O for 2 h at a flow rate of 10 mL/min. Repeated MPLC chromatographic separations of the main collected fractions on reversed-phase columns afforded *trans*-ε-viniferin (**113**, 9 mg), *trans*-resveratrol 3,4′-*O*-diglucoside (**114**, 7 mg), and isotectorigenin (**115**, 21 mg).

### 3.4. Spectroscopic Data of Isolated Compounds

Androsin (4-*O*-β-d-glucopyranosyl-acetovanillone) (**66**): Colorless powder; TLC (silica gel, DCM/MeOH, 8:2): R_f_ = 0.67; UV λ_max_ (MeOH): 275, 308 nm; the molecular formula C_15_H_20_O_8_ was inferred from the [M + Na]^+^ ion peak at *m*/*z* 351.23 in the ESI-MS (positive ion mode) spectrum; ^1^H-NMR (300 MHz, CD_3_OD) δ 7.67 (1H, dd, *J* = 8.5 and 1.9 Hz, H-6), 7.60 (1H, d, *J* = 1.9 Hz, H-2), 7.25 (1H, d, = 8.5 Hz, H-5), 5.06 (1H, d, *J* = 7.2 Hz, H-1′), 3.92 (3H, s, OC*H*_3_), 3.89 (1H, dd, *J* = 12.0 and 1.7 Hz H-6′_b_), 3.71 (1H, dd, *J* = 12.0 and 5.2 Hz H-6′_a_), 3.35–3.55 (4H, m, H-2′,3′,4′,5′), 2.59 (3H, s, COC*H*_3_); ^13^C-NMR (75 MHz, CD_3_OD): aglycone moiety: δ_C_ 133.2 (0, C-1), 112.7 (1, C-2), 150.9 (0, C-3), 152.8 (0, C-4), 116.5 (1, C-5), 124.7 (1, C-6), 199.7 (0, C-7), 26.7 (3, C-8), 56.9 (3, O*C*H_3_); glucose moiety: δ_C_ 102.1 (1, C-1′), 75.0 (1, C-2′), 78.7 (1, C-3′), 71.5 (1, C-4′), 78.2 (1, C-5′), 62.8 (2, C-6′). The numbers in parentheses are the protons attached to the corresponding carbon and were determined by DEPT experiments. The ^1^H and ^13^C-NMR spectra were in accordance with the literature [81]. Acid hydrolysis (3% aqueous H_2_SO_4_, 80 °C, 2 h) provided d-glucose, identical by TLC and comparison of the optical rotation with an authentic sample.

Isovitexin (apigenin 6-*C*-β-glucopyranoside or 4′,5,7-trihydroxy-6-C-β-glucopyranoside) (**109**): Pale yellow powder; TLC (RP-18, MeOH/H_2_O, 6:4): Rf = 0.51; UV λ_max_ (MeOH): 270, 333 nm; the molecular formula C_21_H_20_O_10_ was determined from the [M + Na]^+^ ion peak at *m*/*z* 455.22 in the ESI-MS (positive ion mode) spectrum and the [M − H]^−^ ion peak at *m/z* 431.20 in the ESI-MS (negative ion mode) spectrum; ^1^H-NMR (300 MHz, CD_3_OD) aglycone moiety: δ 7.83 (2H, d, *J* = 8.7 Hz, H-2′ and H-6′), 6.93 (2H, d, *J* = 8.7 Hz, H-3′ and H-5′), 6.59 (1H, s, H-3), 6.50 (1H, s, H-8); glucose moiety: δ 4.91 (1H, d, *J* = 10.0 Hz, H-1″), 4.19 (1H, distorted t, H-2″), 3.90 (1H, dd, *J* = 12.2 and 1.7 Hz, H-6″_b_), 3.75 (1H, dd, *J* = 12.0 and 5.0 Hz, H-6″_a_), 3.52–3.35 (3H, m, H-3″,4″,5″); ^13^C-NMR (75 MHz, CD_3_OD) aglycone moiety: δ_C_ 184.0 (0, C-4), 166.1 (0, C-2), 164.9 (0, C-7), 162.8 (0, C-4′), 162.0 (0, C-5), 158.7 (0, C-9), 129.4 (1 and 1, overlapped C-2′ and C-6′), 123.1 (0, C-1′), 117.0 (1 and 1, overlapped C-3′ and C-5′), 109.2 (0, C-6), 105.2 (0, C-10), 103.8 (1, C-3), 95.2 (1, C-8); glucose moiety: δ_C_ 82.6 (1, C-5″), 80.1 (1, C-3″), 75.2 (1, C-1″), 72.6 (1, C-2″), 71.8 (1, C-4″), 62.9 (2, C-6″). The numbers in parentheses are the protons attached to the corresponding carbon and were determined by DEPT experiments. The ^1^H- and ^13^C-NMR spectra were in accordance with the literature [82]. No rotational isomerism was observed, in accordance with references [68,83].

Swertisin (4′,5-dihydroxy-7-methoxyflavone-6-*C*-β-d-glucopyranoside) (**111**): Pale yellow powder; TLC (RP-18, MeOH/H_2_O, 6:4): R_f_ = 0.53; UV λ_max_ (MeOH): 271, 332 nm; IR (nujol): 3360, 1650, 1605 cm^−1^; the molecular formula C_22_H_22_O_10_ was inferred from the [M + Na]^+^ ion peak at *m*/*z* 469.29 in the ESI-MS (positive ion mode) spectrum and the [M − H]^−^ ion peak at *m*/*z* 445.30 in the ESI-MS (negative ion mode) spectrum; ^1^H-NMR (300 MHz, CD_3_OD) δ 7.90 (2H, d, *J* = 8.6 Hz, H-2′, 6′), 6.94 (2H, d, *J* = 8.6 Hz, H-3′, 5′), 6.76 (1H, s, H-8), 6.67 (1H, s, H-3), 4.87 (1H, d, *J* = 10.0 Hz, H-1″), 4.35–4.48 and 4,15–4.27 (1H overall, 2 m, H-2″ of two rotamers), 3.96 (3H, s, OC*H*_3_), 3.80–3.95 (1H, m, H-6″_b_), 3.55–3.75 (1H, m, H-6″_a_), 3.25-3.45 (3H, m, H-3″, 4″, 5″); ^13^C-NMR (75 MHz, C_5_D_5_N) δ_C_ 181.8 (0, C-4), 163.4 (0, C-2), 162.2 (0, C-7)^+^, 162.2 (0, C-5) ^+^, 160.8 (0, C-4′) ^+^, 156.6 (0, C-9), 127.8 (1 and 1, overlapped C-2′ and C-6′), 120.5 (0, C-1′), 115.9 (1 and 1, overlapped C-3′ and C-5′), 109.8 (0, C-6), 104.6 (0, C-10), 102.8 (1, C-3), 89.1 (1, C-8), 82.1 (1, C-5″), 79.9 (1, C-3″) 73.7 (1, C-2″), 71.4 (1, C-1″) ^#^, 70.5 (1, C-4″) ^#^, 62.4 (2, C-6″), 55.1 (3, O*C*H_3_). ^13^C-NMR (75 MHz, CD_3_OD) δ_C_ 183.9 (0, C-4), 166.6 (0, C-2), 166.6 (0, C-7), 164.0 (0, C-4′) ^+^, 163.9 (0, C-5) ^+^, 159.1 (0, C-9), 129.6 (1 and 1, overlapped C-2′ and C-6′), 122.4 (0, C-1′), 117.4 (1 and 1, overlapped C-3′ and C-5′), 110.5 (0, C-6), 106.4 (0, C-10), 103.9 (1, C-3), 91.3 (1, C-8), 82.6 (1, C-5″), 80.5 (1, C-3″), 74.5 and 74.8 (1, C-2″) *, 72.2 and 72.4 (1, C-1″) *, 71.6 (1, C-4″), 63.4 (3, C-6″), 56.7 and 57.0 (3, O*C*H_3_) *. ^#^,^+^ Assignments are interchangeable; * signal duplication indicating the presence of two rotamers, in the ratio of about 52:48, due to restricted rotation around the *C*(sp^3^)-*C*(sp^2^) glucosyl-flavone linkage (C-6-*C*-1″) [68,83]. The numbers in parentheses are the protons attached to the corresponding carbon and were determined by DEPT experiments.

2”-*O*-α-l-Rhamnosyl swertisin [4′,5-dihydroxy-7-methoxyflavone-6-*C*-(α-l-rhamnopyranosyl-1→2-β-d-glucopyranoside)] (**112**): Yellow powder; TLC (RP-18, MeOH/H_2_O, 7:3): R_f_ = 0.53. UV λ_max_ (MeOH): 270, 332 nm; the molecular formula C_28_H_32_O_14_ was inferred from the [M + Na]^+^ ion peak at *m*/*z* 615.50 in the ESI-MS (positive ion mode) spectrum and the [M − H]^−^ ion peak at *m*/*z* 591.42 in the ESI-MS (negative ion mode) spectrum; ^1^H-NMR (300 MHz, CD_3_OD) δ 7.86 (2H, d, *J* = 8.8 Hz, H-2′, 6′), 6.90 (2H, d, *J* = 8.5 Hz, H-3′, 5′), 6.72 (1H, s, H-8), 6.63 (1H, s, H-3), 5.21 and 5.08 (1H overall, d and d, *J* = 1.6 Hz, H-1‴) *, 4.91 and 4.86 (1H overall, d and d, *J* = 9.8 Hz, H-1″) *, 4.52 and 4.46 (1H overall, dd and dd, *J* = 9.6 and 8.6 Hz, H-2″) *, 3.92 and 3.89 (3H overall, s and s, 7-OC*H*_3_) *, 3.85-3.95 (m, 2H, H-6″_b_ and H-2‴), 3.60–3.75 (1H, m, H-6″_a_), 3.50 (1H, t, *J* = 8.8 Hz, H-3″), 3,39 (1H, t, *J* = 9.0 Hz, H-3‴) *, 3.30-3.45 (3H, m, H-4″, H-5″, H-3‴), 3.08 (1H, t, *J* = 9.5 Hz, H-4‴), 2.30–2.40 and 2.60–2.70 (1H overall, m and m, H-5‴) *, 0.69 and 0.62 (3H overall, d and d, *J* = 6.2 Hz, H_3_-6‴) *, ^13^C-NMR (75 MHz, CD_3_OD) δ_C_ 184.0 and 184.3 (0, C-4) *, 166.5 and 166.7 (0, C-7) *, 165.4 (0, C-2), 162.9 and 163.0 (0, C-4′) *, 161.2 and 161.7 (0, C-5) *, 159.1 and 159.3 (0, C-9) *, 129.5 and 129.6 (1 and 1, overlapped C-2′ and C-6′) *, 123.0 and 122.9 (0, C-1′) *, 117.1 (1 and 1, overlapped C-3′ and C-5′), 110.7 and 111.0 (0, C-6), 105.9 and 106.4 (0, C-10), 104.1 and 104.3 (1, C-3) *, 102.2 and 103.0 (1, C-1‴) *, 91.6 and 92.2 (1, C-8) *, 82.4 and 82.5 (1, C-5″) *, 81.3 and 81.7 (1, C-3″), 76.8 and 78.9 (1, C-2″) *, 73.5 and 73.7 (1, C-4‴) *, 72.8 and 73.2 (1, C-1″) *, 72.4 (1, C-2‴) ^+^, 72.0, 72.1 and 72.2 (1 and 1, C-4″ and C-3‴) *^,+^, 69.9 (1, C-5‴), 63.3 (2, C-6″), 56.7 and 57.1 (3, 7-O*C*H_3_) *, 18.0 and 18.2 (3, C-6‴).* Signal duplication due to the presence of two rotamers [68,83] in the ratio of about 62:38; ^+^ assignments are interchangeable. The numbers in parentheses are the protons attached to the corresponding carbon and were determined by DEPT experiments. Proton and carbon signals were assigned on the basis of COSY, HSQC, HMBC (**112A**), and NOESY correlations. Acid hydrolysis (3% aqueous H_2_SO_4_) of **112** yielded swertisin (**111**) as the aglycone, and L-rhamnose, compared with an authentic sample by TLC and optical rotation.

Trans-ε-viniferin (**113**): Pale yellow powder; mp: 148–150 °C; UV λ_max_ (MeOH): 220, 305 and 320 nm; IR (nujol) 3350, 1610, 1575, 1505, 1330, 1256, 1160 cm^−1^; the molecular formula C_28_H_22_O_6_ was inferred from the [M + H]^+^, [M + Na]^+^ and [2M + Na]^+^ ion peaks at *m*/*z* 455.20, 477.21 and 930.94, respectively, in the ESI-MS (positive ion mode) spectrum and the [M − H]^−^ ion peak at *m*/*z* 453.31 in the ESI-MS (negative ion mode) spectrum; ^1^H-NMR (300 MHz, CD_3_OD) δ 7.11 (2H, d, *J* = 8.5 Hz, H-2′ and H-6′), 7.01 (2H, d, *J* = 8.5 Hz, H-2 and H-6), 6.79 (1H, d, *J* = 16.4 Hz, H-7′), 6.74 (2H, d, *J* = 8.5 Hz, H-3′ and H-5′), 6.62 (2H, d, *J* = 8.5 Hz, H-3 and H-5), 6.60 (1H, d, *J* = 1.8 Hz, H-14′), 6.54 (1H, d, *J* = 16.3 Hz, H-8′), 6.22 (1H, d, *J* = 1.8 Hz, H-12′), 6.16-6.14 (3H, m, H-10, H-12 and H-14), 5.33 (1H, d, *J* = 6.6 Hz, H-7), 4.32 (1H, d, *J* = 6.6 Hz, H-8); ^13^C-NMR (75 MHz, CD_3_OD) δ_C_ 162.7 (0, C-11′), 160.1 (0 and 0, overlapped C-11 and C-13), 159.8 (0, C-13′) ^+^, 158.5 (0, C-4′) ^+^, 158.4 (0, C-4) ^+^, 147.4 (0, C-9), 136.9 (0, C-9′), 133.9 (0, C-1), 130.4 (0, C-1′), 130.3 (1, C-7′), 128.8 (1 and 1, overlapped C-2′ and C-6′), 128.2 (1 and 1, overlapped C-2 and C-6), 123.7 (1, C-8′), 120.1 (0, C-10′), 116.4 (1 and 1, overlapped C-3 and C-5), 116.3 (1 and 1, overlapped C-3′ and C-5′), 107.4 (1 and 1, overlapped C-10 and C-14), 104.4 (1, C-14′), 102.3 (1, C-12), 96.9 (1, C-12′), 94.8 (1, C-7), and 58.3 (1, C-8); ^+^ assignments are interchangeable. The numbers in parentheses are the protons attached to the corresponding carbon and were determined by DEPT experiments. The NMR spectra matched those reported in literature [84,85]; however, some assignments were corrected.

Resveratrol 3,4′-*O*-di-β-d-glucopyranoside (**114**): Pale yellow powder; the molecular formula C_26_H_32_O_13_ was inferred from the [M + Na]^+^ ion peak at *m*/*z* 575.28 in the ESI-MS (positive-ion mode) spectrum; ^1^H-NMR (300 MHz, CD_3_OD) δ 7.44 (2H, d, *J* = 8.7 Hz, H-2′ and H-6′), 7.05 (2H, d, *J* = 8.7 Hz, H-3′ and H-5′), 7.03 (1H, d, *J* = 16.4 Hz, H-8), 6.90 (1H, d, *J* = 16.4 Hz, H-7), 6.78 (1H, br s, H-2), 6.57 (1H, br s, H-6), 6.44 (1H, t, *J* = 2.1 Hz, H-4), 4.89 (1H, d, *J* = 8.0 Hz, H-1″)^+^, 4.86 (1H, d, *J* = 8.0 Hz, H-1‴)^+^, 3.85-3.97 (2H, m, H-6″_b_ and H-6‴_b_), 3.67 (2H, two overlapped dd, *J* = 12.0 and 5.1 Hz, H-6″_a_ and H-6‴_a_), 3.35–3.50 (8H, m, H-2″, H-2‴, H-3′’, H-3‴, H-4′’, H-4‴, H-5″, H-5‴); ^+^assignments are interchangeable; ^13^C-NMR (75 MHz, CD_3_OD) δ_C_ 160.5 (0, C-3), 159.6 (0, C-5), 158.8 (0, C-4′), 141.1 (0, C-1), 133,1 (0, C-1′), 129.4 (1, C-8), 128.7 (1 and 1, overlapped C-2′ and C-6′), 128.2 (1, C-7), 117.9 (1 and 1, overlapped C-3′ and C-5′), 108.5 (1, C-6), 107.1 (1, C-2), 104.4 (1, C-4), 102.4 and 102.2 (1 each, C-1″ and C-1‴), 78.4, 78.3, 78.2, 78,1 (1 each, C-5″, C-5‴, C-3″ and C-3‴), 74.9 and 74.9 (1 each, C-2″ and C-2‴), 71.5 and 71.4 (1 each, C-4″ and C-4‴), 62.6 and 62.5 (2 each, C-6″ and C-6‴). The numbers in parentheses are the protons attached to the corresponding carbon and were determined by DEPT experiments. Signal assignments to protons and carbons were established on the basis of COSY, HSQC, HMBC, and NOESY correlations. The data are in accordance with those reported in literature [86]. Acid hydrolysis (3% aqueous H_2_SO_4_) provided d-glucose, identical by TLC and comparison of the optical rotation with an authentic sample.

Isotectorigenin (pseudotectorigenin or psi-tectorigenin) (4′,5,7-trihydroxy-8-methoxyisoflavone) (**115**): Pale yellow powder, TLC (RP-18, MeOH/H_2_O, 8:2): R_f_ = 0.52. UV λ_max_ (MeOH): 269, 336 (sh) nm; the molecular formula C_16_H_12_O_6_ was inferred from the [2M + Na]^+^ ion peak at *m*/*z* 622.97 and the [M+Na]^+^ ion peak at *m*/*z* 323.12 in the ESI-MS (positive ion mode) spectrum, and the [M − H]^−^ ion peak at *m*/*z* 299.17 in the ESI-MS (negative ion mode) spectrum. ^1^H-NMR (300 MHz, CD_3_OD) δ 7.81 (1H, s, H-2), 7.30 (2H, d, *J* = 8.4 Hz, H-2′, 6′), 6.79 (2H, d, *J* = 8.4 Hz, H-3′, 5′), 6.09 (1H, s, H-6), 3.77 (3H, s, 8-OC*H*_3_). ^13^C-NMR (75 MHz, CD_3_OD) δ_C_ 181.0 (0, C-4), 159.7 (0, C-7), 158.8 (0, C-4′), 156.7 (0, C-9), 153.7 (0, C-5), 153.3 (1, C-2), 137.2 (0, C-8), 131.5 (1 and 1, C-2′ and C-6′), 124.1 (0, C-3), 123.4 (0, C-1′), 116.3 (1 and 1, C-3′ and C-5′), 102.3 (0, C-10), 98.2 (1, C-6), 60.6 (3, 8-O*C*H_3_). The numbers in parentheses are the protons attached to the corresponding carbon and were determined by DEPT experiments. Proton and carbon signals were assigned on the basis of HSQC, HMBC, and NOESY correlations. The data are in accordance with those reported in literature [87].

### 3.5. Free Radical Scavenging Activity

Briefly, a 0.3 mM solution of DPPH in MeOH was prepared. To 1 mL of this solution, 3 mL of sample or extract solution in 10% aqueous MeOH at different concentrations (10, 25, 50, 100, 150, 200, 250, 350 μg/mL) was added. Subsequently, the mixture was shaken vigorously and incubated for 30 min at 22 °C in the dark until a stable absorbance value (*A*) at 517 nm was obtained, that was measured using a UV-Visible spectrophotometer (Lambda 25 UV/Vis spectrometer N.3903, Perkin Elmer instruments, Waltham, MA, USA). A lower absorbance of the reaction mixture indicated higher free radical scavenging (FRS) activity. The DPPH solution (1 mL), plus 10% MeOH (3 mL), was used as the control. The FRS% was calculated using the formula: [1 − (A_sample_/A_control_)] × 100. The curve of the % scavenging activity against the concentration was plotted for each sample using the MS Excel-based program to calculate the EC_50_ value, i.e., the concentration (μg/mL or μM/L) of the sample required to scavenge 50% of the initial DPPH concentration. Each analysis was carried out in triplicate and the mean ± SD (*n* = 3) was calculated. Ascorbic acid (Sigma-Aldrich) was used as a standard antiradical agent. The lower the EC_50_ value, the higher the sample antiradical activity. The antiradical activity was also expressed as ascorbic acid equivalents (AAEs) (Table 2), i.e., μg ascorbic acid equivalents/μg sample. These values were calculated using the formula: EC_50_ ascorbic acid (μg/mL)/EC_50_ sample (μg/mL) [77].

### 3.6. Total Antioxidant Capacity (TAOC—Ammonium Phosphomolybdate Assay)

Briefly, samples of dry extracts or standard ascorbic acid, dissolved in MeOH/distilled H_2_O (50:50), were combined with 3.0 mL of reagent solution (0.6 M sulfuric acid, 28 mM sodium phosphate and 4 mM ammonium molybdate) to achieve a series of eight final concentrations in the range of 12–450 μg/mL. The tubes were capped and incubated in a boiling water bath at 95 °C for 90 min. The samples were cooled to 22 °C and absorbance was measured at 695 nm against the blank using the cited UV-Visible spectrophotometer. From each series of measures, a sigmoidal curve was obtained by data interpolation and the EC_50_ value of each sample was calculated as the concentration corresponding to 50% activity. The blank contained 3.0 mL of the reagent solution and 0.3 mL of MeOH/distilled H_2_O (50:50), and it was incubated under the same conditions as the samples. Each analysis was carried out in triplicate and the mean ± SD (*n* = 3) was calculated. The TAOC values were expressed as μg ascorbic acid equivalents/μg extract (Table 2). They were calculated using the formula: EC_50_ (μg/mL) ascorbic acid)/EC_50_ (μg/mL) extract. The EC_50_ of ascorbic acid used in TAOC calculation was 26.12 ± 0.56 μg/mL.

### 3.7. Acid Hydrolysis of Compounds ***112*** and ***114***

Compounds **112** and **114** (2.0 mg each) were separately dissolved in 3% aqueous H_2_SO_4_ in a sealed vial and heated at 90 °C for 45 min. After cooling to room temperature and extraction with CHCl_3_, the aqueous layer was repeatedly evaporated to dryness with the aid of MeCN. The residues were identified as l-(+)-rhamnose and d-(+)-glucose, respectively, by TLC and optical rotation upon comparison with authentic samples.

## 4. Conclusions

A dozen *Iris* species are used in the traditional medicine of Kurdistan. In the first part of this paper, we have reported the structures of the main constituents, traditional uses and biological activities found in the literature for the few species growing in Kurdistan that have been investigated so far. The most characteristic secondary metabolites are various terpenoids, among which iridal derivatives are the most typical ones, and phenolic derivatives, among which isoflavones predominate. Most of these *Iris* metabolites exhibited various bioactivities. Based on these data, we then investigated, for the first time, the contents of the methanolic extracts of *I. postii* aerial parts and rhizomes. L-tryptophan, androsin (**66**), apigenin 6-*C*-glucoside (isovitexin) (**109**), swertisin (**111**), and 2″-*O*-rhamnosyl swertisin (**112**) were isolated from the aerial parts, whereas chromatographic separation of the extract from rhizomes afforded *trans*-ε-viniferin (**113**), *trans*-resveratrol 3,4′-O-diglucoside (**114**), and isotectorigenin (**115**). To the best of our knowledge, this is the first finding of compounds **112**–**115** in the genus *Iris*. Isolated compounds showed a wide range of bioactivities, in addition to the excellent radical-scavenging properties exhibited in this investigation by 2″-*O*-α-l-rhamnosyl- swertisin (**112**), ε-viniferin (**113**), and resveratrol 3,4′-*O*-di-β-d-glucopyranoside (**114**). Thus, isovitexin (**109**) has been reported to be an anti-inflammatory, antihyperglycemic, sedative agent with insulin secretagogue properties and to display antioxidant, anti-inflammatory, neuroprotective, anti-diabetic, antitumor effects [88,89]; swertisin (**111**) exhibited antioxidant, anti-inflammatory, antihyperglycemic activities with insulin secretagogue and adenosine A1 receptor antagonist properties [90]; swertisin (**111**) and 2″-O-rhamnosylswertisin (**112**) exhibited strong α-glucosidase inhibitory activity in vitro [91] and effective mechanical antinociceptive properties [81]; ε-viniferin exhibited relatively strong inhibition of α-glucosidase in vitro [92] and inhibited both human LDL and HDL oxidation in vitro [85]; resveratrol diglucoside **114** decreased ethanol-induced oxidative DNA damage in mouse brain cells, possibly via inhibition of oxidative stress [93], and displayed highly selective antiproliferative activity against tumor cells [94].

In conclusion, the few *Iris* species growing in Kurdistan that have been investigated so far demonstrated to be novel viable sources of various bioactive compounds. Moreover, the remarkable antioxidant and radical scavenging activities of the methanol extracts of aerial parts and rhizomes of *I. postii*, as well as the anti-inflammatory properties reported for different isolated compounds, validate the traditional medicinal use of this plant in Kurdistan. Further studies aimed to evaluate the in vivo potential of *Iris* extracts in various models and to isolate and identify the antioxidant principles occurring in the most polar fractions of the methanolic extract of *I. postii* aerial parts shall be carried out in due time.

## Figures and Tables

**Table 1:** Biological activities reported for the isoflavonoids isolated from *Iris germanica*.

Compound	Bioactivity	Reference
Germanaism A (**1**)	Cytotoxic activity IC_50_ = 43.9 ± 1.5 μM (MTT); 4.5 ± 0.4 μM (ATP)	[34]
Tectoridin (**9**)	Free-radical scavenger, antioxidant, anti-inflammatory, antiproliferative, oestrogenic, anti-alcohol injury, hepatoprotective effects	[48]
Iridin (**10**)	Potent anti-inflammatory effects (induced paw edema test)	[24]
Iridin A (**11**)	High antioxidant activity; α-amylase inhibitory activity	[38,40]
Irisolidone 7-*O*-β-d-glucopyranoside (**14**)	CyP1A inhibitor; QR inhibitor; DPPH scavenger	
Irilone (**17**)	Cytotoxic activity IC_50_ = 47.7 ± 3.5 μM (MTT), 17.7 ± 1.4 μM (ATP); potent anti-inflammatory effects (induced paw edema test): high antioxidant activity; α-amylase inhibitory activity; potent inhibitor of cytochrome P450 1A activity (IC_50_ = 0.3 ± 0.1 μM); immunomodulatory activity; CyP1A inhibitor; moderate QR inhibitor; DPPH scavenger	[34] [24] [38,40] [42] [43]
Irilone 4′-*O*-β-d-glucopyranoside (**19**)	Potent anti-inflammatory effects (induced paw edema test)	[24]
8-Hydroxyirilone (**21**)	High antioxidant activity; α-amylase inhibitory activity	[38,40]
8-Hydroxyirilone 5-methyl ether (**22**)	High antioxidant activity; α-amylase inhibitory activity	[38,40]
Iriflogenin (**23**)	Potent inhibitor of cytochrome P450 1A activity (IC_50_ = 1.4 ± 0.6 μM); CyP1A inhibitor; weak DPPH scavenger	[42]
Irifloside (**25**)	Cytotoxic activity IC_50_ = 21.5 ± 4.4 μM (MTT); 19.4 ± 1.3 μM (ATP)	[34]
Irisolone (nigricin) (**28**)	High anti-inflammatory activity	[14]
Nigricanin (iriskashmirianin) (**29**)	CyP1A inhibitor; moderate QR inhibitor; weak DPPH scavenger	[42]
Iriskashmirianin A (**30**)	Cytotoxic activity IC_50_ = 20.9 ± 2.7 μM (MTT); 4.3 ± 0.9 μM (ATP)	[34]
5,7-Dihydroxy-3-(3′-hydroxy-4′,5′-dimethoxy)-8-methoxy-4H-1-benzopyran-4-one (**33**)	Significant anti-inflammatory activity	[14]
Tectorigenin (**35**)	Antifungal, free radical scavenger, antioxidant, anti-inflammatory, anti-angiogenic, antiproliferative, antineoplastic, hypoglycaemic, oestrogenic, hepatoprotectiv, antithrombotic, cardiovascular, anti-alcohol injury activities; in patented pharmaceutical compositions for the treatment of hormone-related diseases	[48,49]
Irigenin (**37**)	Potent anti-inflammatory effects (induced paw edema test and inhibition against superoxide); α-amylase inhibitory activity; potent inhibitor of cytochrome P450 1A activity (IC_50_ = 1.2 ± 0.3 μM); CyP1A inhibitor; moderate QR inhibitor, DPPH scavenger	[14,24] [38,40] [42]
Irigenin S (**38**)	Potent anti-inflammatory effects (induced paw edema test)	[24]
Irisolidone (**42**)	Potent anti-inflammatory effects (induced paw edema test); α-amylase inhibitory activity; immunomodulatory activity; CyP1A inhibitor; QR inhibitor; DPPH scavenger; antiproliferative activity against amelanotic melanoma and large lung carcinoma cells; antioxidant properties	[24] [38,40] [43] [50] [51]
Iristectorigenin A (**43**)	Weak anti-inflammatory activity	[14]
Isoflavone (**44**)	Moderate anti-inflammatory activity	[14]
5,7,8-Trihydroxy-3-(4-methoxyphenyl)-2-methyl-4*H*-chromen-4-one (**47**)	Significant inhibition of TRAP in NF-kB ligand-induced osteoclastic RAW 264.7 cells (66.67 ± 2.71%)	[47]
6,7-Dihydroxy-3-(4-methoxyphenyl)-2-methyl-4*H*-chromen-4-one (**48**)	Significant inhibition of TRAP in NF-kB ligand-induced osteoclastic RAW 264.7 cells (57.32 ± 2.46%)	[47]

**Table 2:** Antiradical and antioxidant activities of extracts and compounds isolated from *Iris postii*.

Sample	DPPH Scavenging Activity			Total Antioxidant *Capacity* (TAOC) ^b^
	EC_50_ (μg/mL)	EC_50_ (μM/L)	AAE ^a^	
Androsin (**66**)	48.94 ± 0.09	149.21	0.48	-
Isovitexin (**109**)	50.97 ± 1.11	117.99	0.46	-
Swertisin (**111**)	37.35 ± 0.13	83.74	0.63	-
2″-*O*-α-l-Rhamnosyl swertisin (**112**)	26.52 ± 0.11	44.79	0.89	-
ε-Viniferin (**113**)	26.06 ± 0.01	57.39	0.90	-
*Trans*-resveratrol 3,4′-*O*-di-β-d-glucopyranoside (**114**)	22.91 ± 0.05	41.50	1.03	-
Isotectorigenin (**115**)	34.87 ± 0.13	116.23	0.67	-
Ascorbic acid	23.52 ± 0.22	133.63	1.00	-
IPA ^c^	19.21 ± 0.01	-	1.22	0.39
IPAD ^d^	62.79 ± 0.03	-	0.37	0.21
IPR ^e^	46.28 ± 0.12	-	0.51	0.29
IPRB ^f^	39.11 ± 0.10	-	0.60	0.46

^a^ Expressed as μg ascorbic acid equivalents/μg sample; ^b^ expressed as μg ascorbic acid equivalents/μg extract; ^c^ methanol extract of aerial parts; ^d^ dichloromethane fraction from IPA; ^e^ methanol extract of rhizomes; ^f^ butanol fraction from IPR.

## Data Availability

Raw data are available in supplementary material.

## Supplementary Materials

NMR and MS spectra of isolated compounds are available online, Figure S1: ^1^H-NMR spectrum (300 MHz, CD_3_OD) of androsin, Figure S2: ^13^C-NMR spectrum (75 MHz, CD_3_OD) of androsin, Figure S3: ESI-MS (positive ion mode) spectrum of androsin, Figure S4: ^1^H-NMR spectrum (300 MHz, CD_3_OD) of isovitexin, Figure S5: ^13^C-NMR spectrum (75 MHz, CD_3_OD) of isovitexin, Figure S6: ESI-MS (positive ion mode) spectrum of isovitexin, Figure S7: ESI-MS (negative ion mode) spectrum of isovitexin, Figure S8: ^1^H-NMR spectrum (300 MHz, CD_3_OD) of swertisin, Figure S9: ^13^C-NMR spectrum (75 MHz, CD_3_OD) of swertisin, Figure S10: ^13^C-NMR spectrum (75 MHz, C_5_D_5_N) of swertisin, Figure S11: ESI-MS (positive ion mode) spectrum of swertisin, Figure S12: ESI-MS (negative ion mode) spectrum of swertisin, Figure S13: ^1^H-NMR spectrum (300 MHz, CD_3_OD) of 2”-*O*-α-l-rhamnosyl swertisin, Figure S14: COSY spectrum of 2”-*O*-α-l-rhamnosyl swertisin, Figure S15: ^13^C-NMR spectrum (75 MHz, CD_3_OD) of 2”-*O*-α-l-rhamnosyl swertisin, Figure S16: HSQC spectrum of 2”-*O*-α-l-rhamnosyl swertisin, Figure S17: HMBC spectrum of 2”-*O*-α-l-rhamnosyl swertisin, Figure S18: HMBC spectrum (enlargement 1) of 2”-*O*-α-l-rhamnosyl swertisin, Figure S19: HMBC spectrum (enlargement 2) of 2”-*O*-α-l-rhamnosyl swertisin, Figure S20: NOESY spectrum of 2”-*O*-α-l-rhamnosyl swertisin, Figure S21: ESI-MS spectra (positive and negative ion mode) of 2”-*O*-α-l-rhamnosyl swertisin, Figure S22: ^1^H-NMR spectrum (300 MHz, CD_3_OD) of tryptophan, Figure S23: ^13^C-NMR spectrum (75 MHz, CD_3_OD) of tryptophan, Figure S24: ESI-MS (positive ion mode) spectrum of tryptophan, Figure S25: ^1^H-NMR spectrum (300 MHz, CD_3_OD) of isotectorigenin, Figure S26: ^13^C-NMR spectrum (75 MHz, CD_3_OD) of isotectorigenin, Figure S27: ESI-MS (positive ion mode) spectrum of isotectorigenin, Figure S28: ESI-MS (negative ion mode) spectrum of isotectorigenin, Figure S29: ^1^H-NMR spectrum (300 MHz, CD_3_OD) of *trans*-ε-viniferin, Figure S30: ^13^C-NMR spectrum (75 MHz, CD_3_OD) of *trans*-ε-viniferin, Figure S31: ESI-MS (positive ion mode) spectrum of *trans*-ε-viniferin, Figure S32: ESI-MS (negative ion mode) spectrum of *trans*-ε-viniferin, Figure S33: ^1^H-NMR spectrum (300 MHz, CD_3_OD) of resveratrol-3,4′-*O*-di-β-d-glucopyranoside, Figure S34: ^13^C-NMR spectrum (75 MHz, CD_3_OD) of resveratrol-3,4′-*O*-di-β-d-glucopyranoside, Figure S35: HSQC spectrum of resveratrol 3,4′-*O*-di-β-d-glucopyranoside, Figure S36: HMBC spectrum of resveratrol-3,4′-*O*-di-β-d-glucopyranoside, Figure S37: NOESY spectrum of resveratrol 3,4′-*O*-di-β-d-glucopyranoside, Figure S38: ESY-MS spectrum (positive ion mode) of resveratrol-3,4′-*O*-di-β-d-glucopyranoside.

## Sample Availability

Samples of the compounds are not available from the authors.
