# Supplementary material for: Phytochemistry and Biological Activities of Iris Species Growing in Iraqi Kurdistan and Phenolic Constituents of the Traditional Plant Iris postii
Source: Molecules. 2021 Jan 7;26(2):264. doi: 10.3390/molecules26020264 (PMC7827031; doi:10.3390/molecules26020264)
Supplement: Supplementary file 1 [file molecules-26-00264-s001.pdf]

Supplementary Information

Phytochemistry and Biological Activities of *Iris* Species Growing in Iraqi Kurdistan and Phenolic Constituents of the Traditional Plant *Iris postii*

Hawraz Ibrahim M. Amin, Faiq H. S. Hussain, Soran K. Najmaldin, Zaw Min Thu, Mohammed F. Ibrahim, Gianluca Gilardoni and Giovanni Vidari

Spectroscopic data of isolated compounds

NMR and MS spectra of androsin (**66**)

Figure S1. ^{1}H-NMR spectrum (300 MHz, CD_3OD) of androsin page 3
Figure S2. ^{13}C-NMR spectrum (75 MHz, CD_3OD) of androsin page 4
Figure S3. ESI-MS (positive ion mode) spectrum of androsin page 5

NMR and MS spectra of isovitexin (**109**)

Figure S4. ^{1}H-NMR spectrum (300 MHz, CD_3OD) of isovitexin page 6
Figure S5. ^{13}C-NMR spectrum (75 MHz, CD_3OD) of isovitexin page 7
Figure S6. ESI-MS (positive ion mode) spectrum of isovitexin page 8
Figure S7. ESI-MS (negative ion mode) spectrum of isovitexin page 9

NMR and MS spectra of swertisin (**111**)

Figure S8. ^{1}H-NMR spectrum (300 MHz, CD_3OD) of swertisin page 10
Figure S9. ^{13}C-NMR spectrum (75 MHz, CD_3OD) of swertisin page 11
Figure S10. ^{13}C-NMR spectrum (75 MHz, C_5D_5N) of swertisin page 12
Figure S11. ESI-MS (positive ion mode) spectrum of swertisin page 13
Figure S12. ESI-MS (negative ion mode) spectrum of swertisin page 14

NMR and MS spectra of 2″-O-α-L-rhamnosyl swertisin (**112**)

Figure S13. ^{1}H-NMR spectrum (300 MHz, CD_3OD) of 2''-*O*-α-L-rhamnosyl swertisin page 15
Figure S14. COSY spectrum of 2''-*O*-α-L-rhamnosyl swertisin page 16
Figure S15. ^{13}C-NMR spectrum (75 MHz, CD_3OD) of 2''-*O*-α-L-rhamnosyl swertisin page 17
Figure S16. HSQC spectrum of 2''-*O*-α-L-rhamnosyl swertisin page 18
Figure S17. HMBC spectrum of 2''-*O*-α-L-rhamnosyl swertisin page 19
Figure S18. HMBC spectrum (enlargement 1) of 2''-*O*-α-L-rhamnosyl swertisin page 20
Figure S19. HMBC spectrum (enlargement 2) of 2''-*O*-α-L-rhamnosyl swertisin page 21
Figure S20. NOESY spectrum of 2''-*O*-α-L-rhamnosyl swertisin page 22
Figure S21. ESI-MS spectra (positive and negative ion mode) of 2''-*O*-α-L-rhamnosyl swertisin page 23

NMR and MS spectra of tryptophan

Figure S22. ^{1}H-NMR spectrum (300 MHz, CD_3OD) of tryptophan page 24
Figure S23. ^{13}C-NMR spectrum (75 MHz, CD_3OD) of tryptophan page 25
Figure S24. ESI-MS (positive ion mode) spectrum of tryptophan page 26

NMR and MS spectra of isotectorigenin (115)

Figure S25. ^{1}H-NMR spectrum (300 MHz, CD_3OD) of isotectorigenin page 27
Figure S26. ^{13}C-NMR spectrum (75 MHz, CD_3OD) of isotectorigenin page 28
Figure S27. ESI-MS (positive ion mode) spectrum of isotectorigenin page 29
Figure S28. ESI-MS (negative ion mode) spectrum of isotectorigenin page 30

NMR and MS spectra of trans-ε-viniferin (113)

Figure S29. ^{1}H-NMR spectrum (300 MHz, CD_3OD) of *trans*-ε-viniferin page 31
Figure S30. ^{13}C-NMR spectrum (75 MHz, CD_3OD) of *trans*-ε-viniferin page 32
Figure S31. ESI-MS (positive ion mode) spectrum of *trans*-ε-viniferin page 33
Figure S32. ESI-MS (negative ion mode) spectrum of *trans*-ε-viniferin page 34

NMR and MS spectra of resveratrol 3,4′-O-di-β-D-glucopyranoside (114)

Figure S33. ^{1}H-NMR spectrum (300 MHz, CD_3OD) of resveratrol 3,4′-*O*-di-β-D-glucopyranoside page 35
Figure S34. ^{13}C-NMR spectrum (75 MHz, CD_3OD) of resveratrol 3,4′-*O*-di-β-D-glucopyranoside page 36
Figure S35. HSQC spectrum of resveratrol 3,4′-*O*-di-β-D-glucopyranoside page 37
Figure S36.HMBC spectrum of resveratrol 3,4′-*O*-di-β-D-glucopyranoside page 38
Figure S37. NOESY spectrum of resveratrol 3,4′-*O*-di-β-D-glucopyranoside page 39
Figure S38. ESY-MS spectrum (positive ion mode) of resveratrol 3,4′-*O*-di-β-D-glucopyranoside page 40

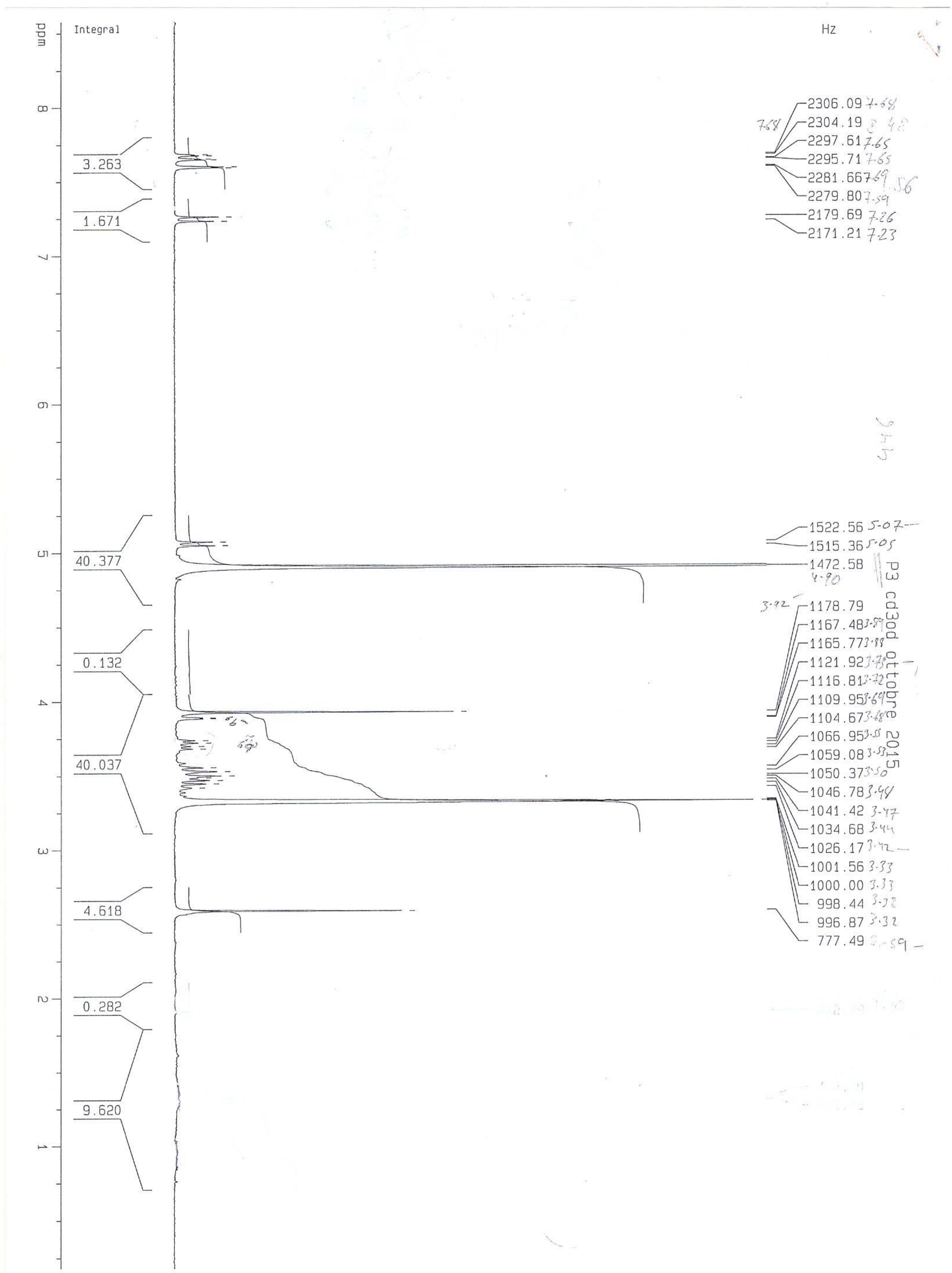

Figure S1. ^{1}H-NMR spectrum (300 MHz, CD_3OD) of androsin (**66**).

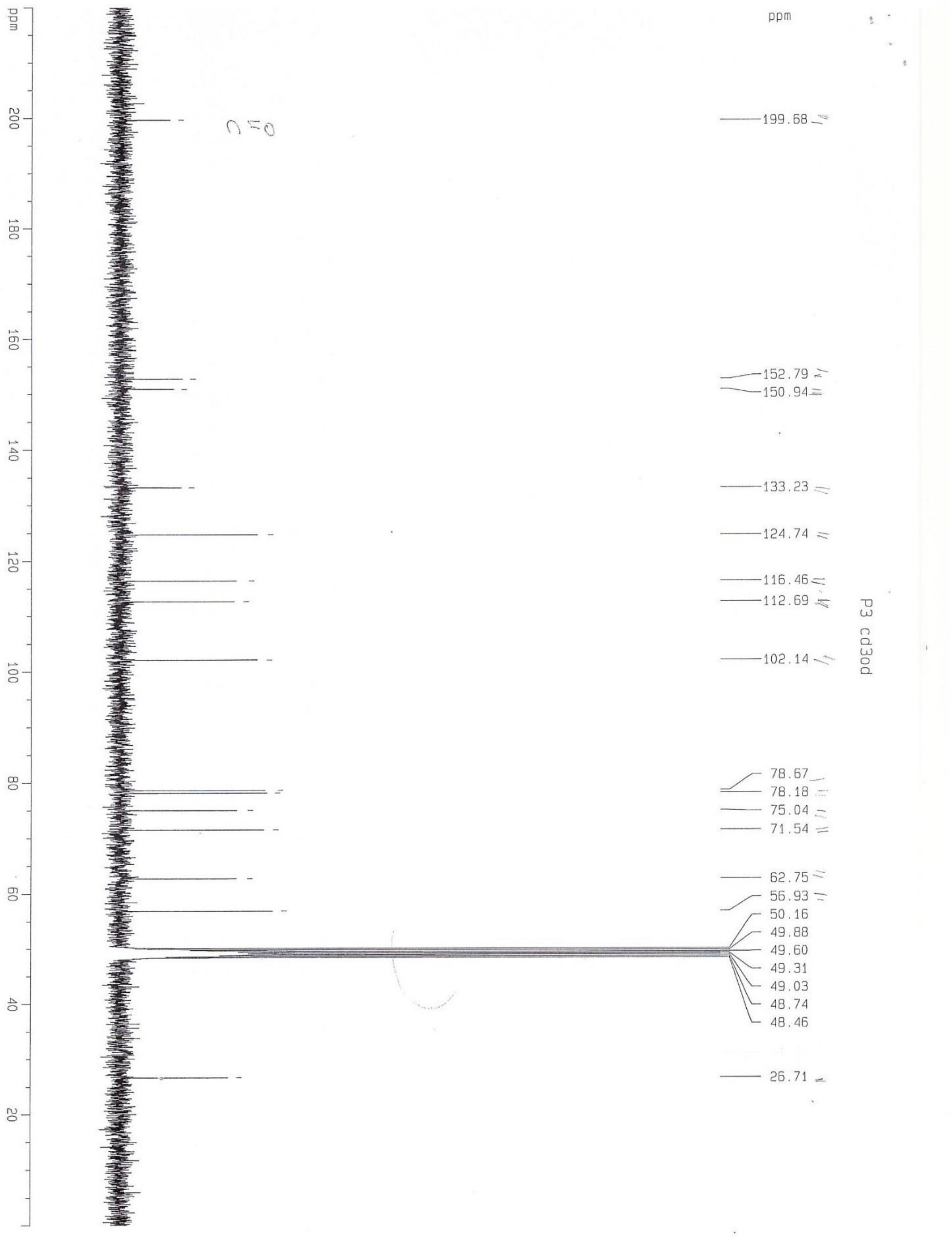

Figure S2. ^{13}C-NMR spectrum (75 MHz, CD_3OD) of androsin (**66**)

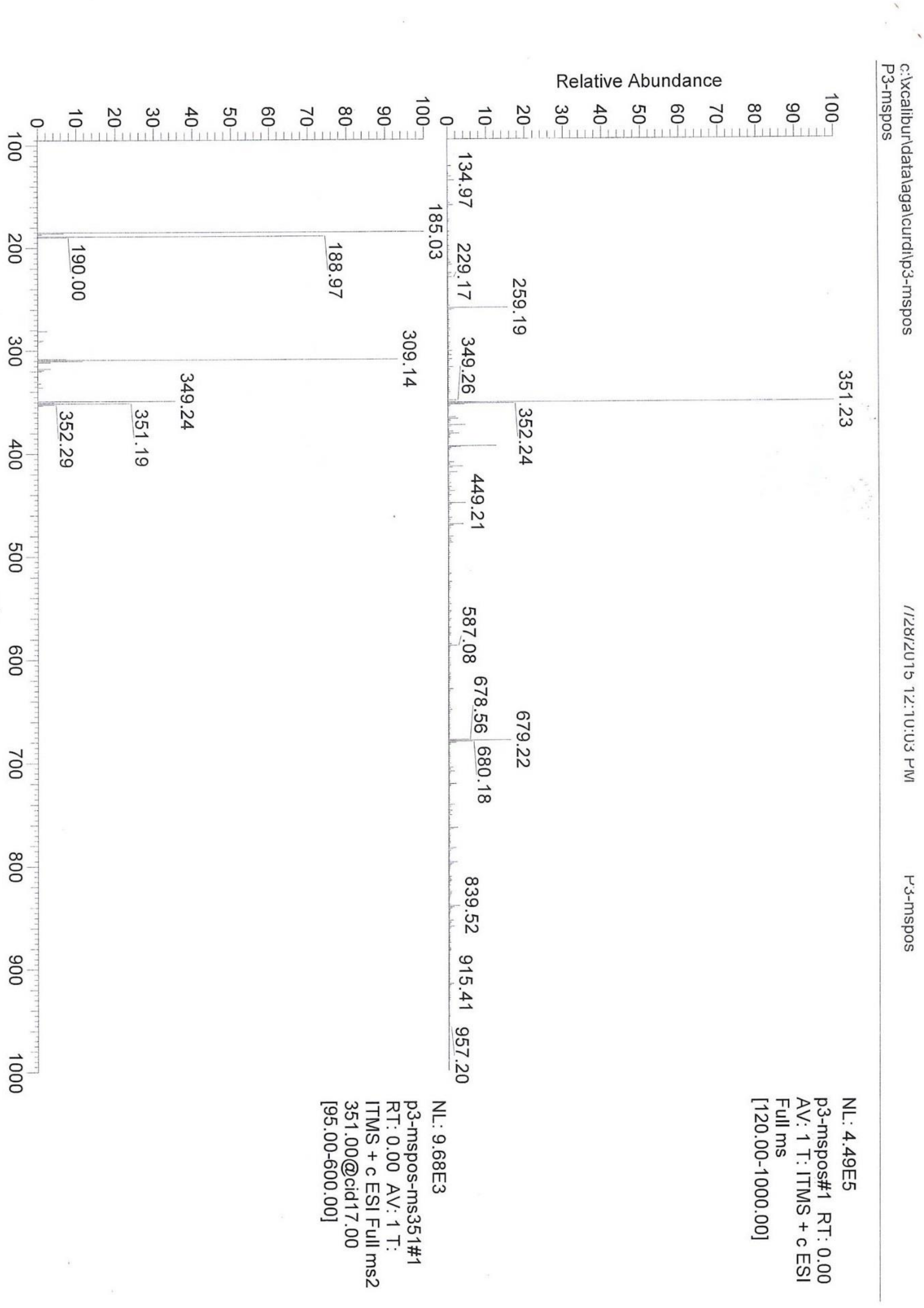

Figure S3. ESI-MS (positive ion mode) spectrum of androsin (**66**)

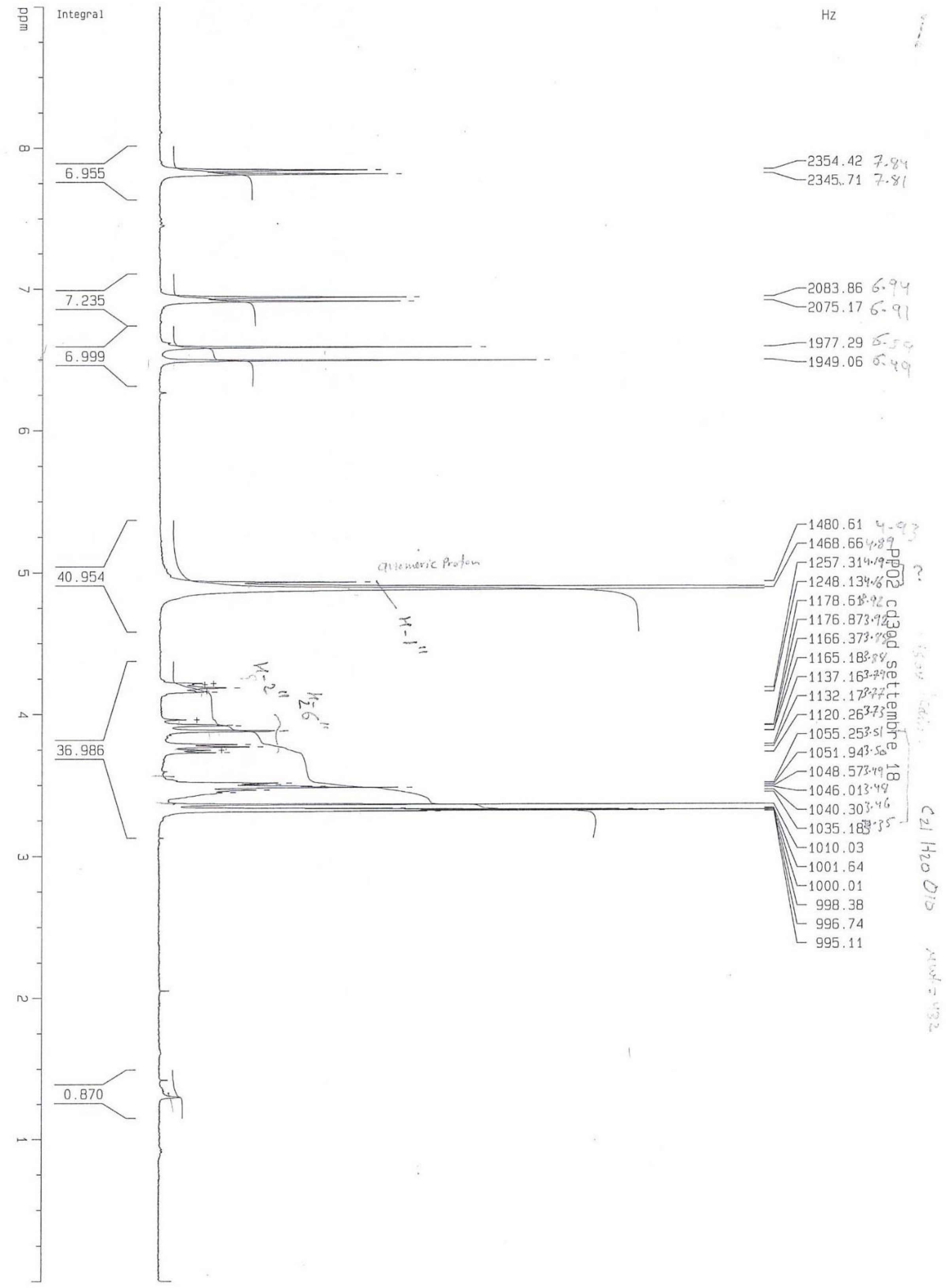

Figure S4. ^{1}H-NMR spectrum (300 MHz, CD_3OD) of isovitexin (**109**)

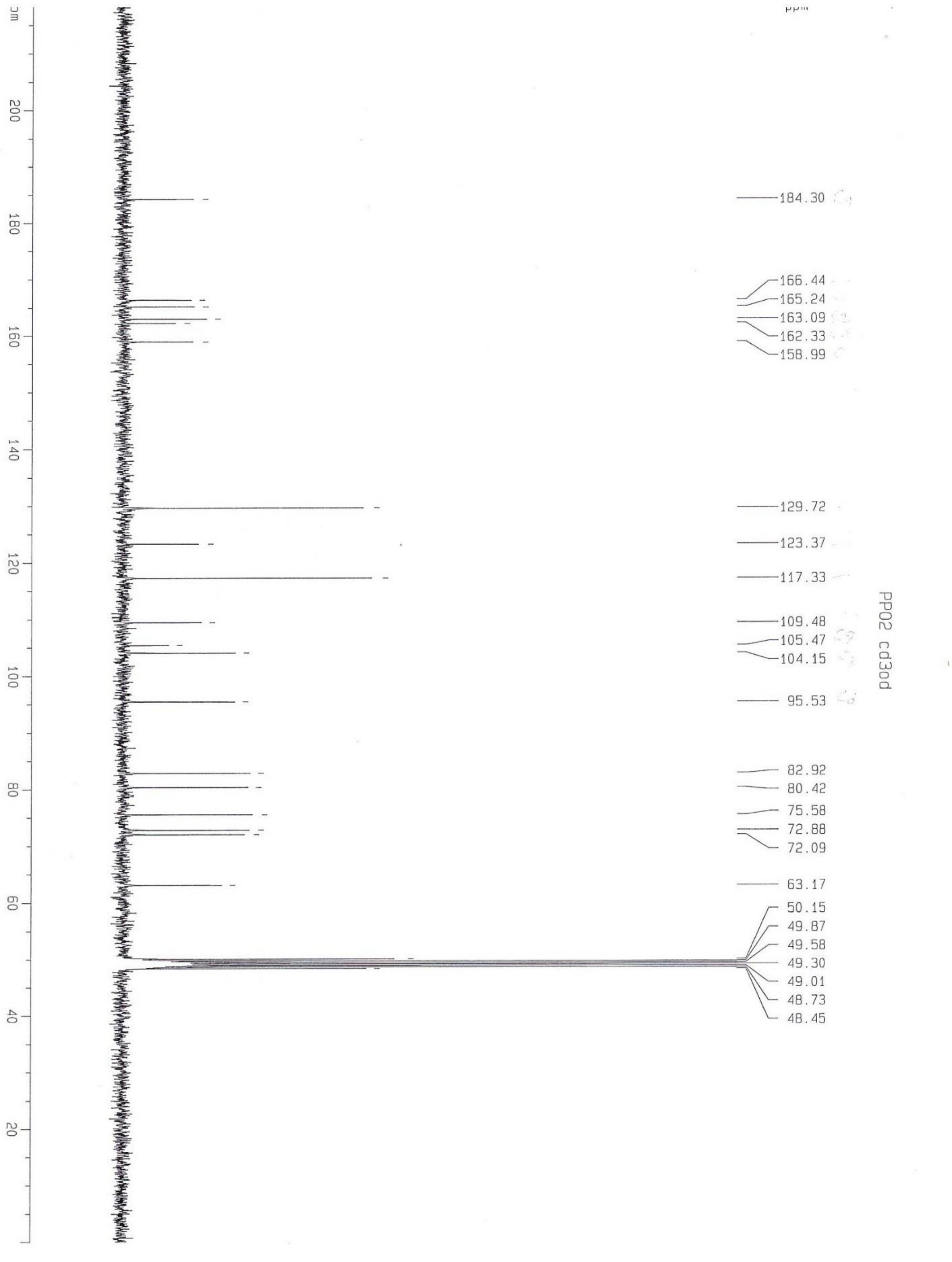

Figure S5. ^{13}C-NMR spectrum (75 MHz, CD_3OD) of isovitexin (**109**)

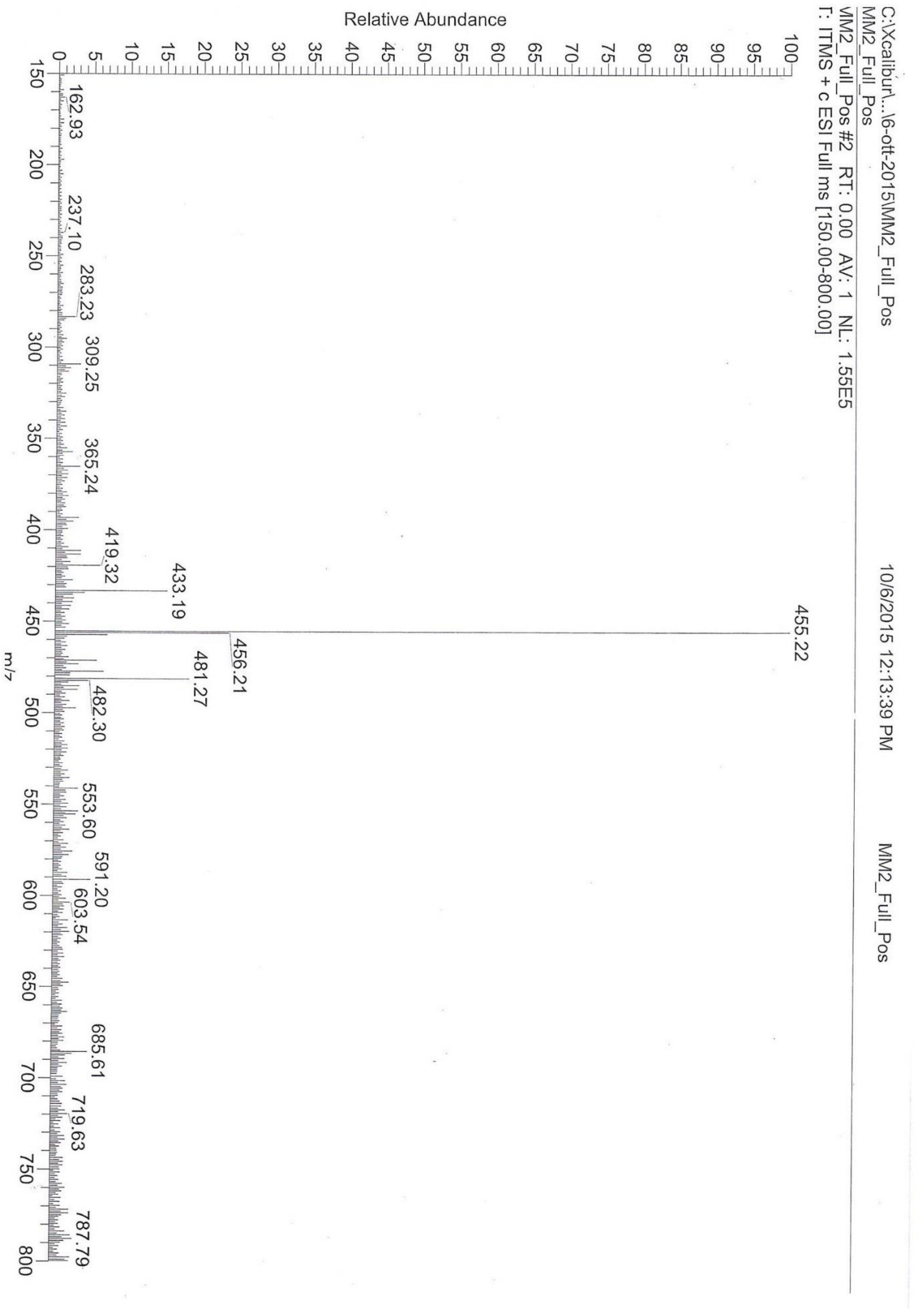

Figure S6. ESI-MS (positive ion mode) spectrum of isovitexin (**109**)

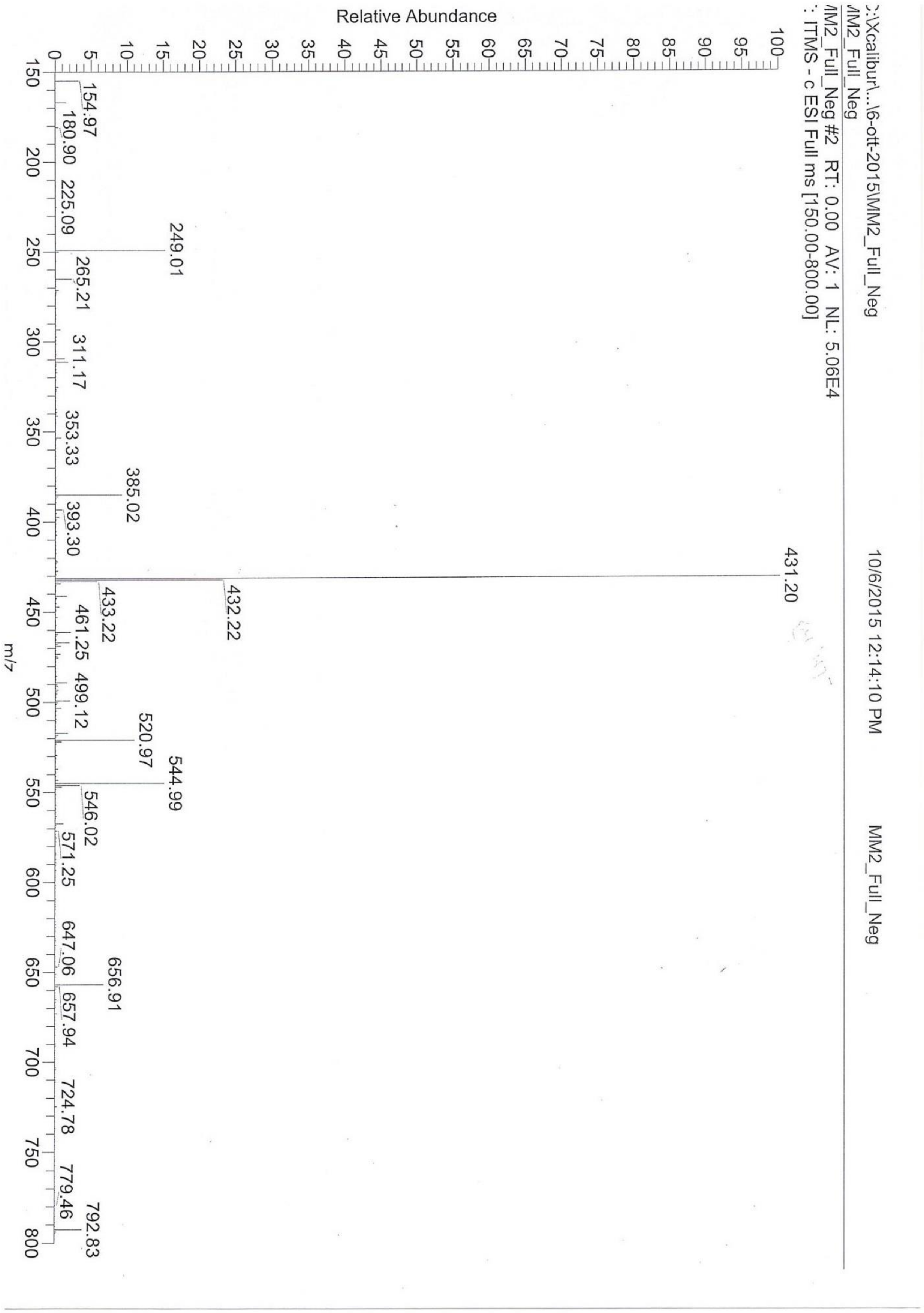

Figure S7. ESI-MS (negative ion mode) spectrum of isovitexin (**109**)

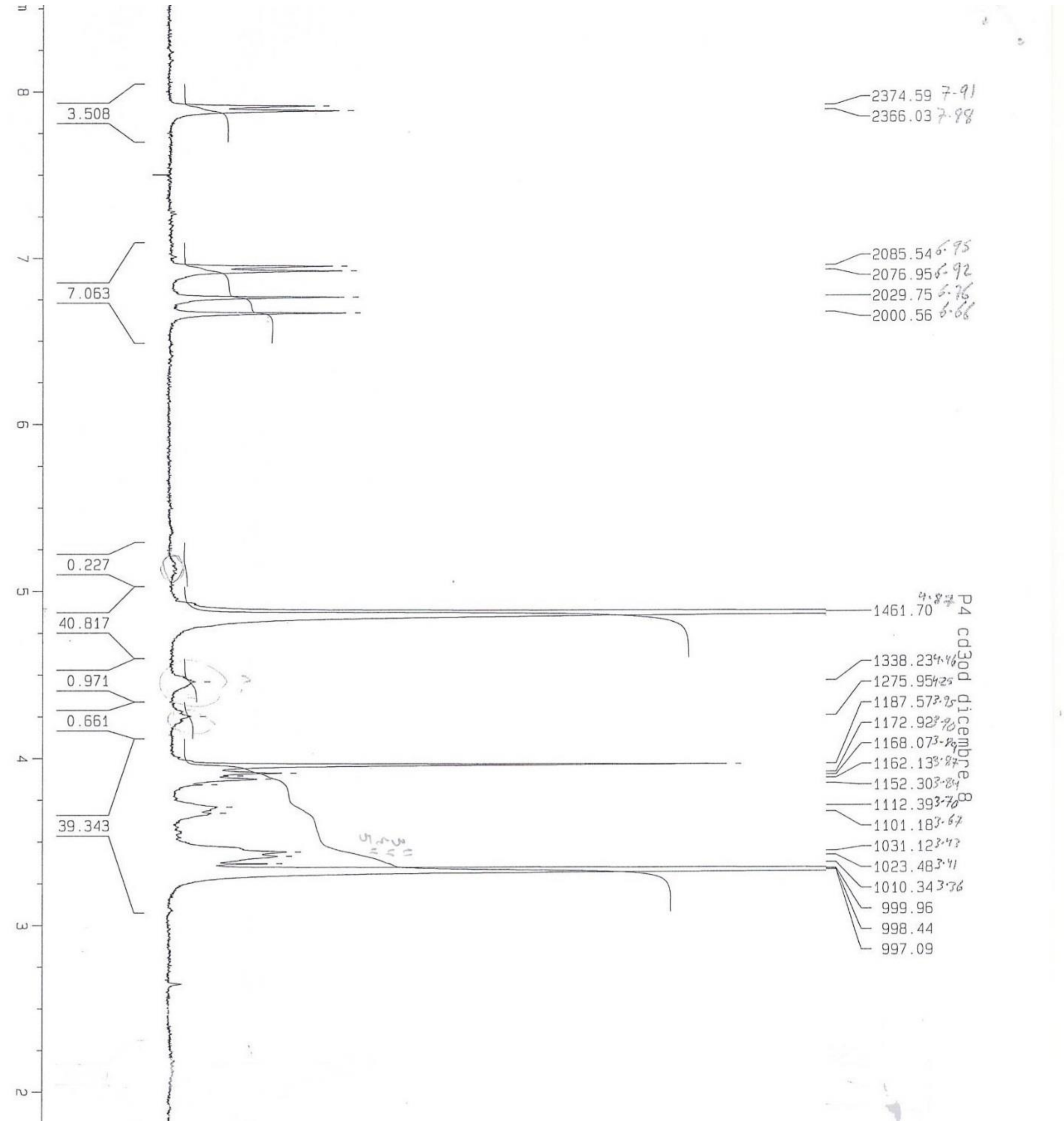

Figure S8. ^{1}H-NMR spectrum (300 MHz, CD_3OD) of swertisin (**111**)

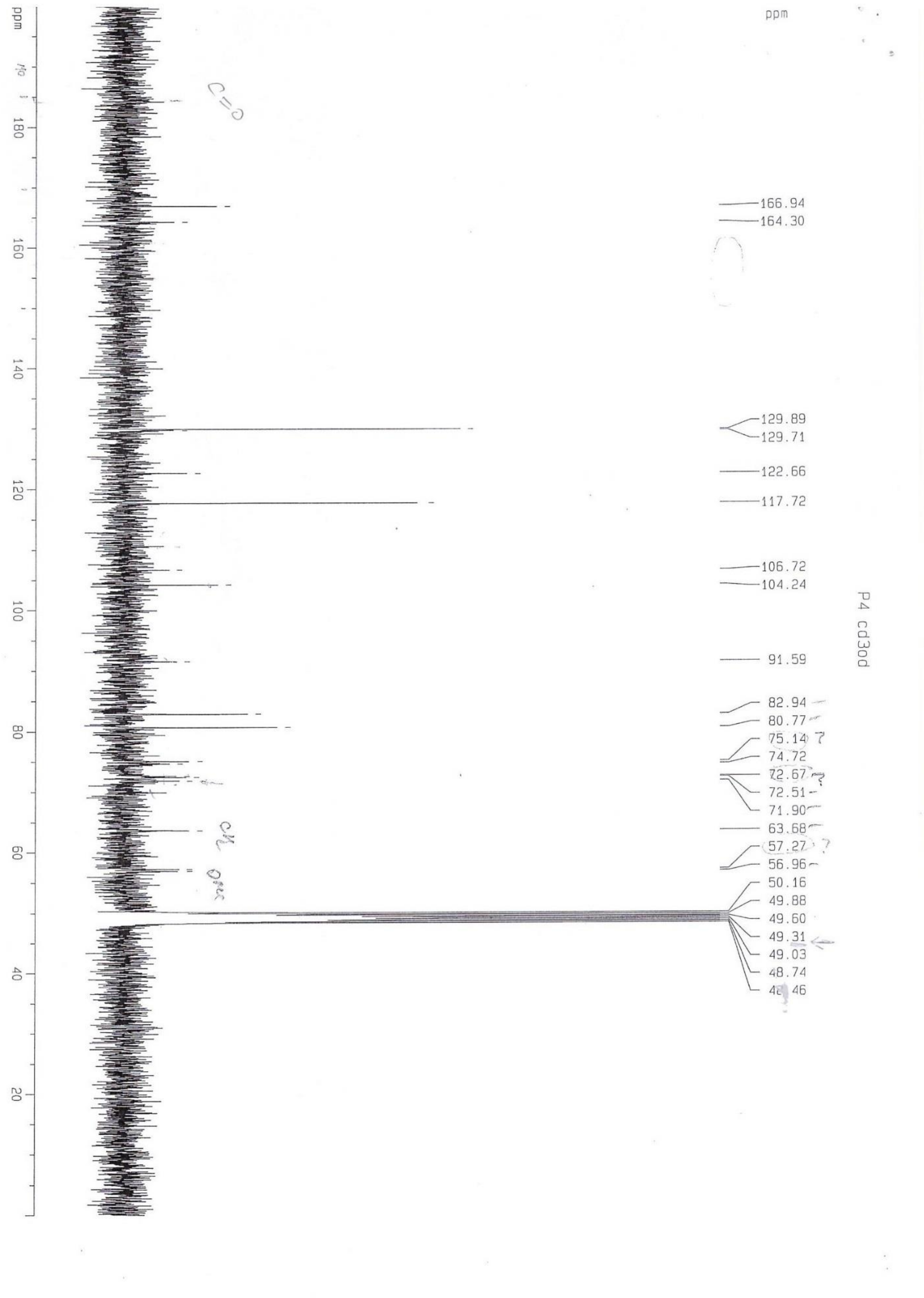

Figure S9. ^{13}C-NMR spectrum (75 MHz, CD_3OD) of swertisin (**111**)

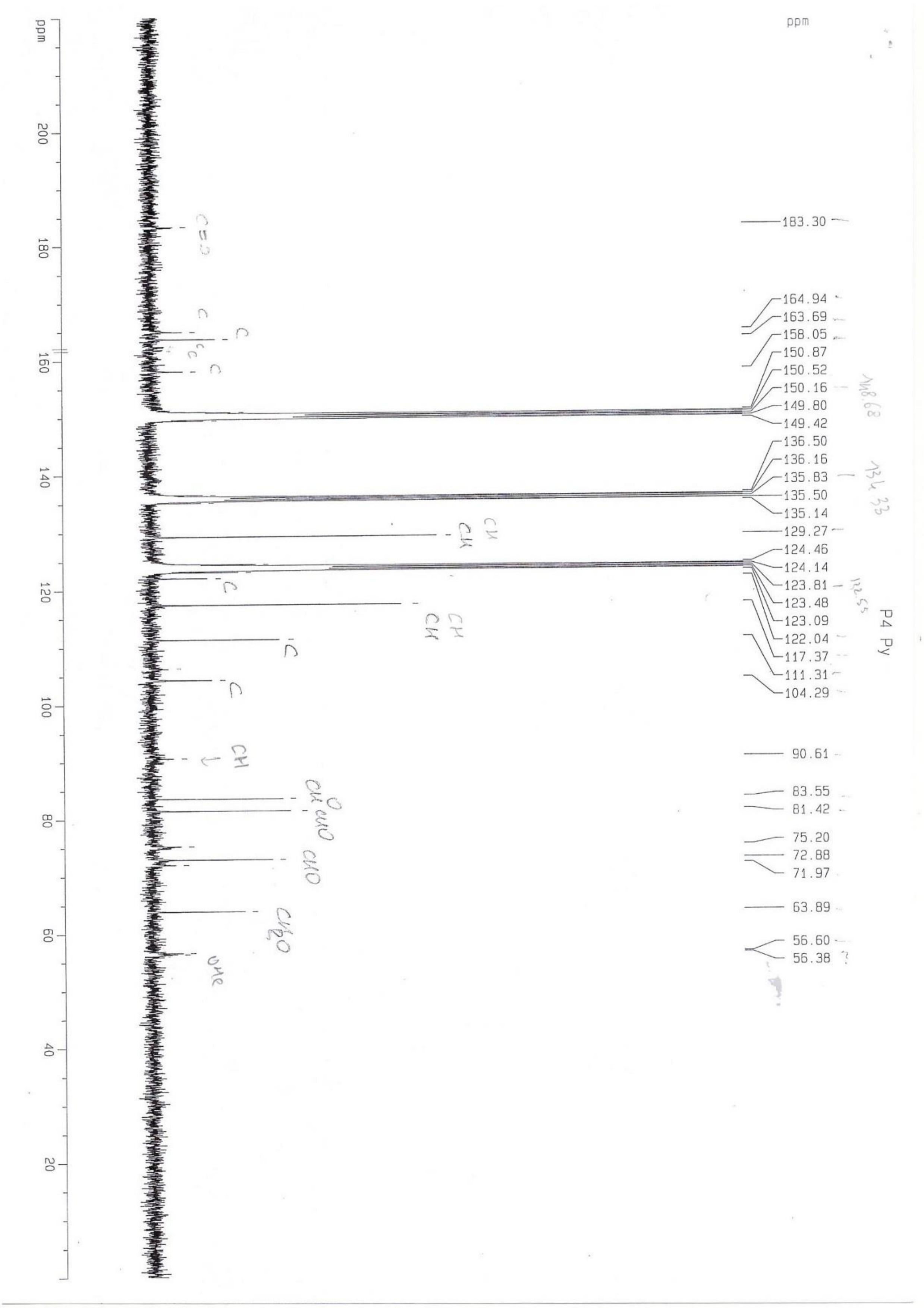

Figure S10. ^{13}C-NMR spectrum (75 MHz, C_5D_5N) of swertisin (**111**)

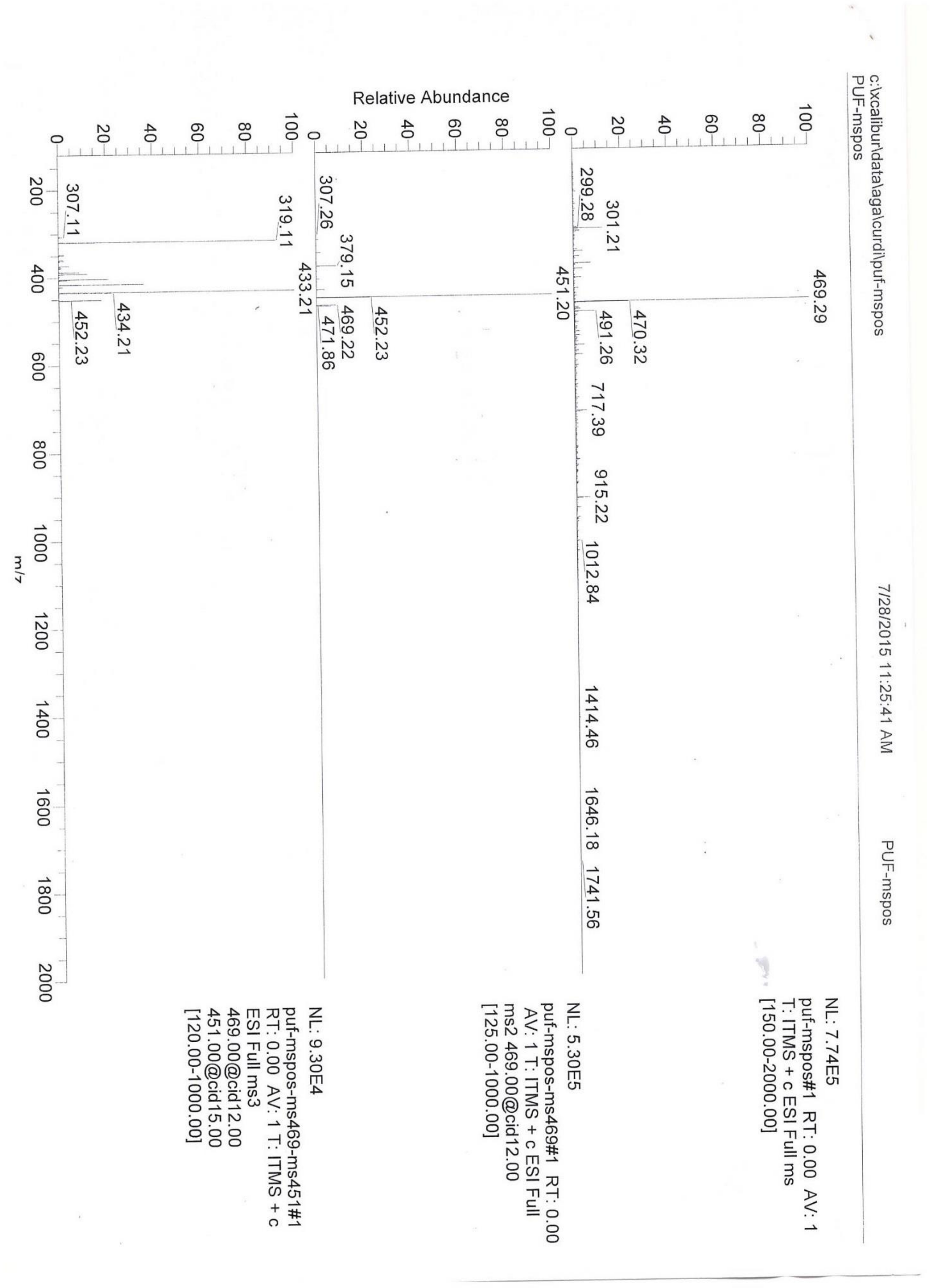

Figure S11. ESI-MS (positive ion mode) spectrum of swertisin (**111**)

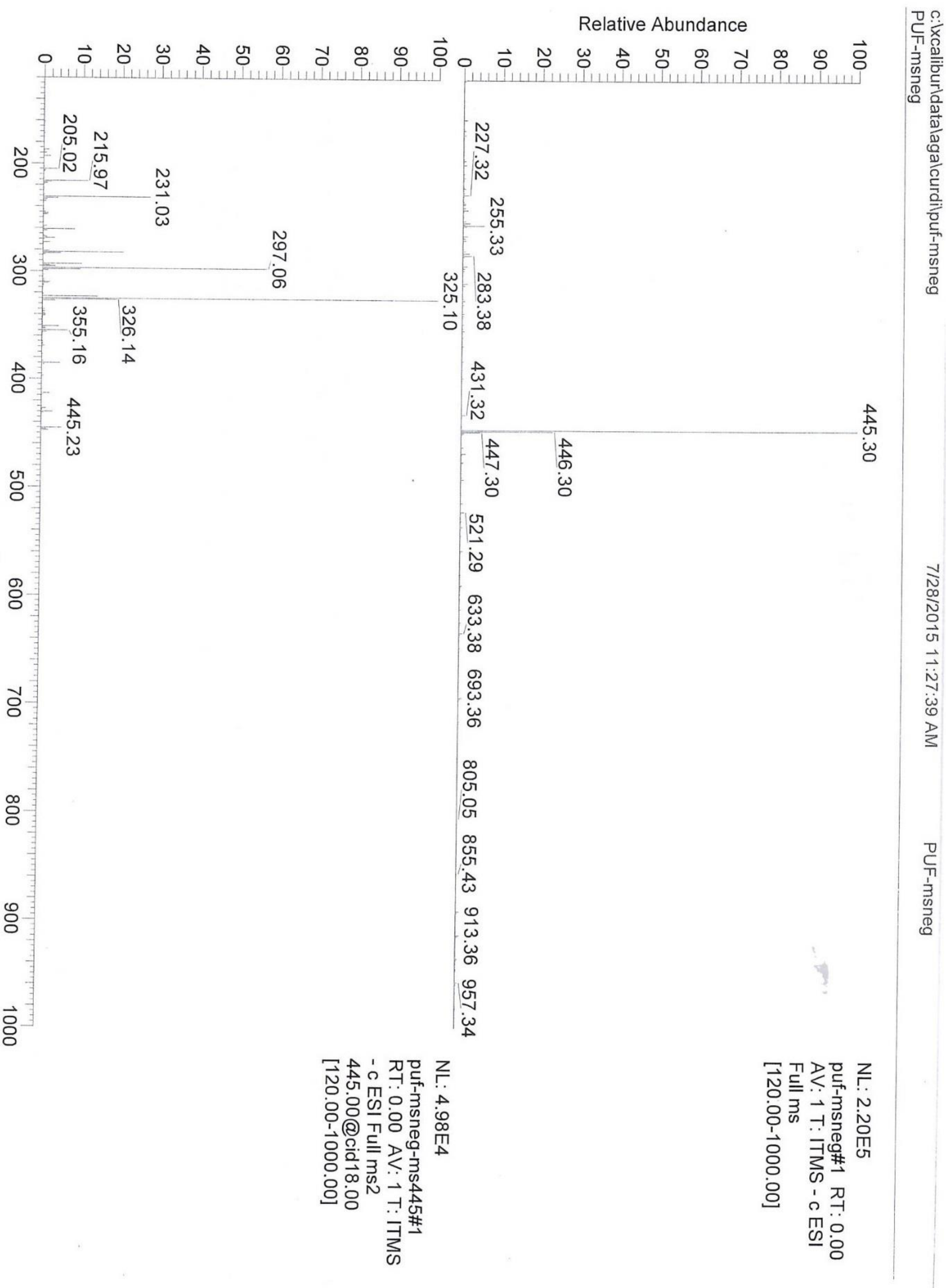

Figure S12. ESI-MS (negative ion mode) spectrum of swertisin (**111**)

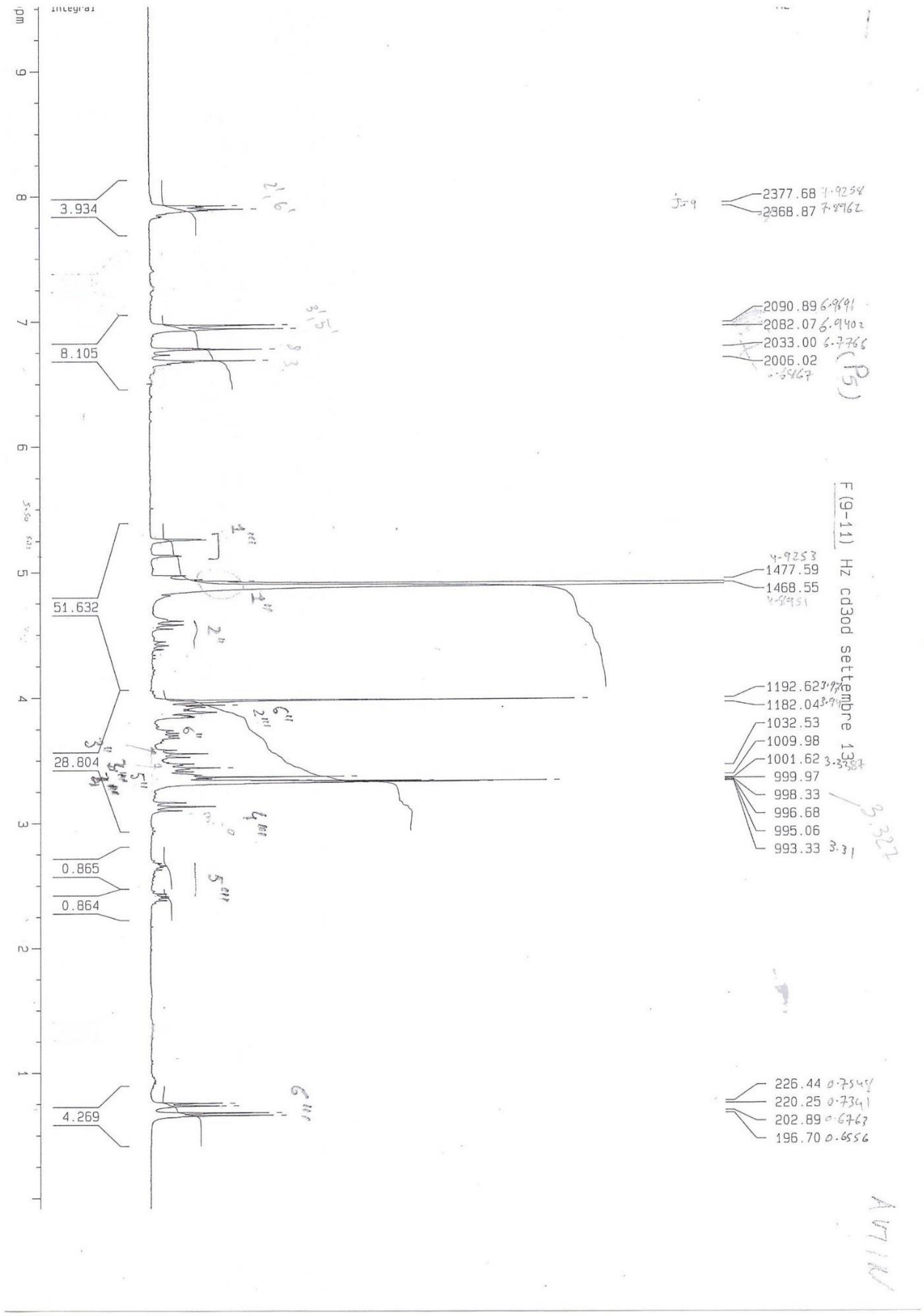

Figure S13. ^{1}H-NMR spectrum (300 MHz, CD_3OD) of 2''-*O*-α-L-rhamnosyl swertisin (**112**)

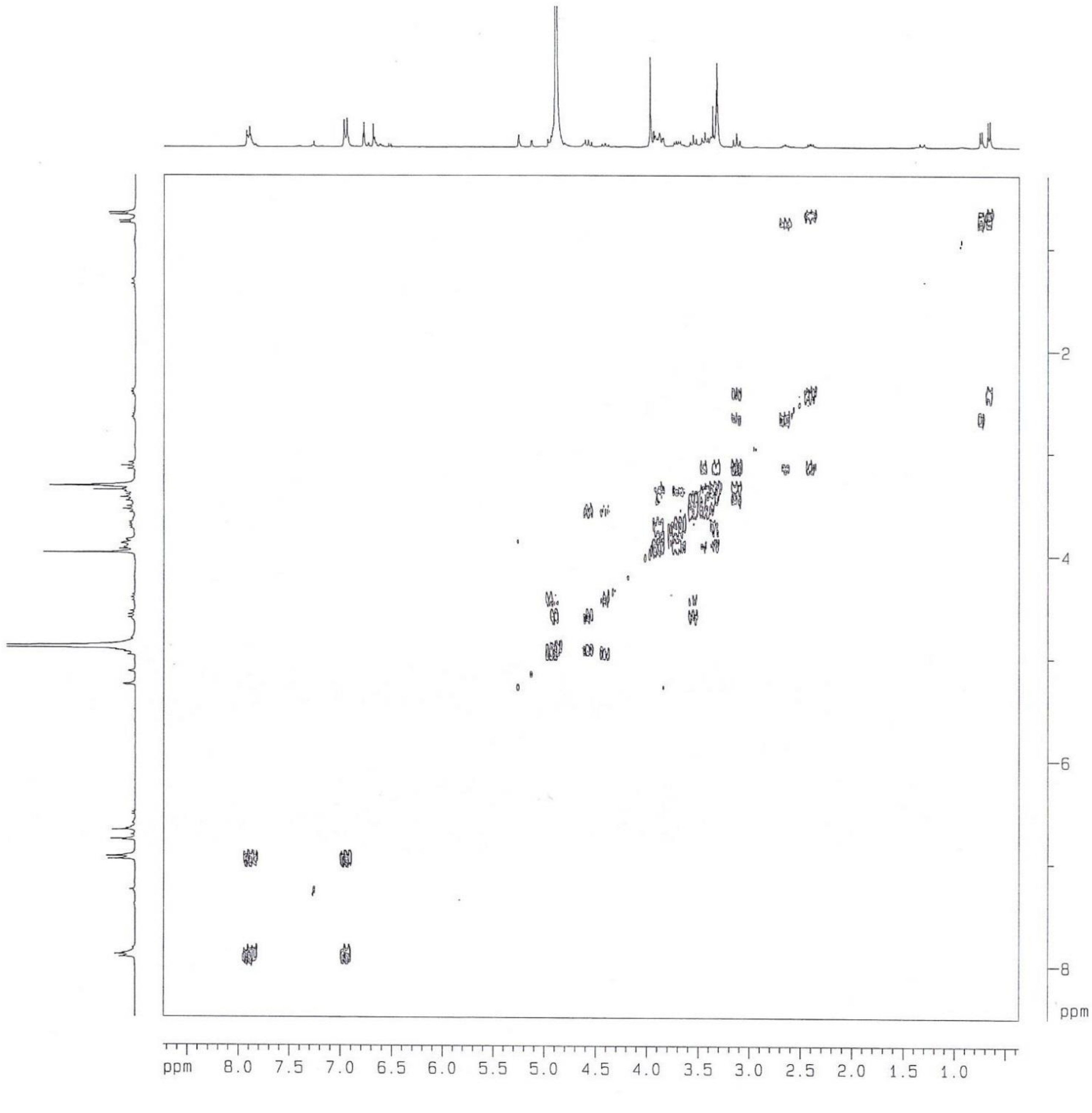

Figure S14. COSY spectrum of 2''-*O*-α-L-rhamnosyl swertisin (**112**)

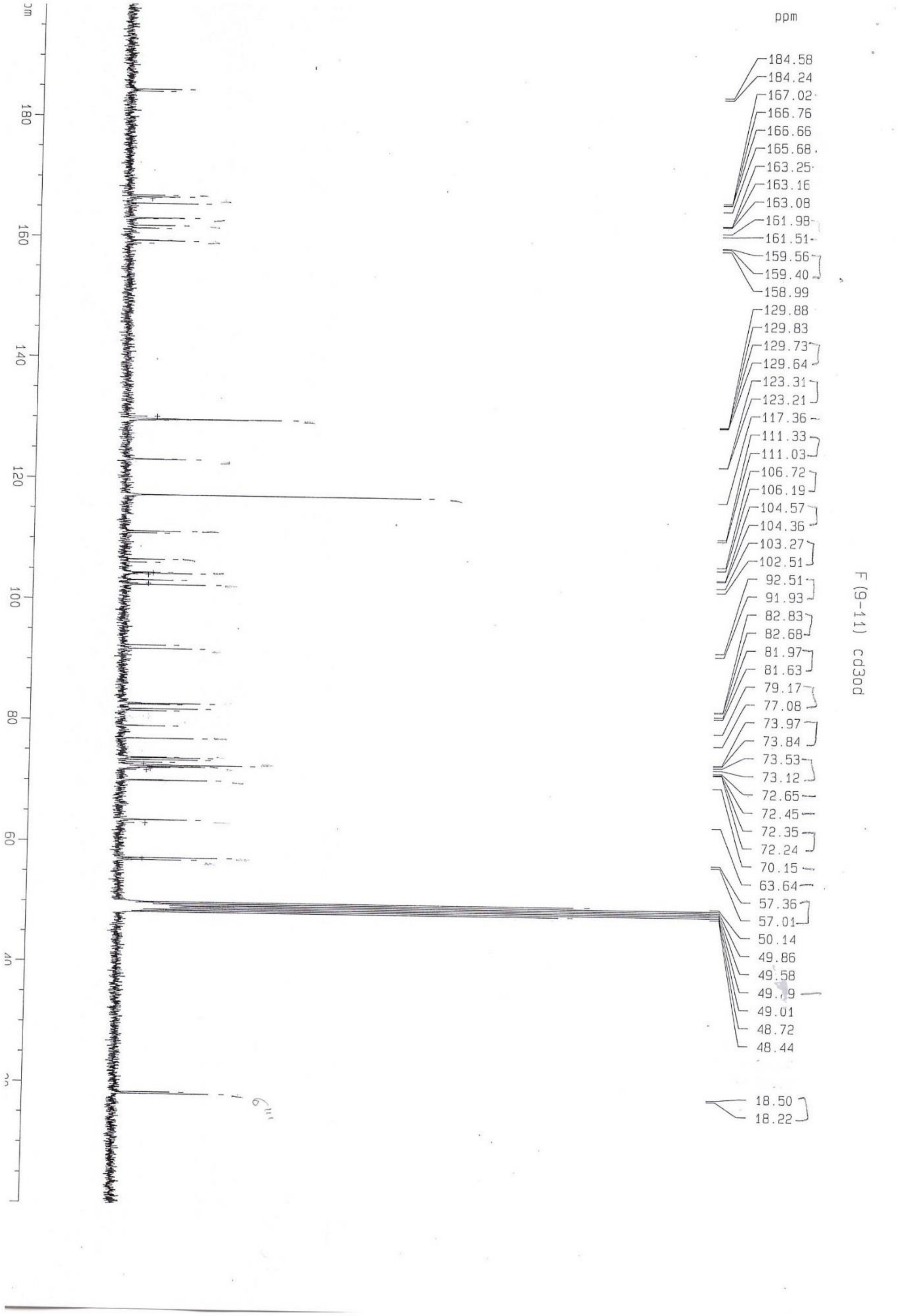

Figure S15. ^{13}C-NMR spectrum (75 MHz, CD_3OD) of 2''-*O*-α-L-rhamnosyl swertisin (**112**)

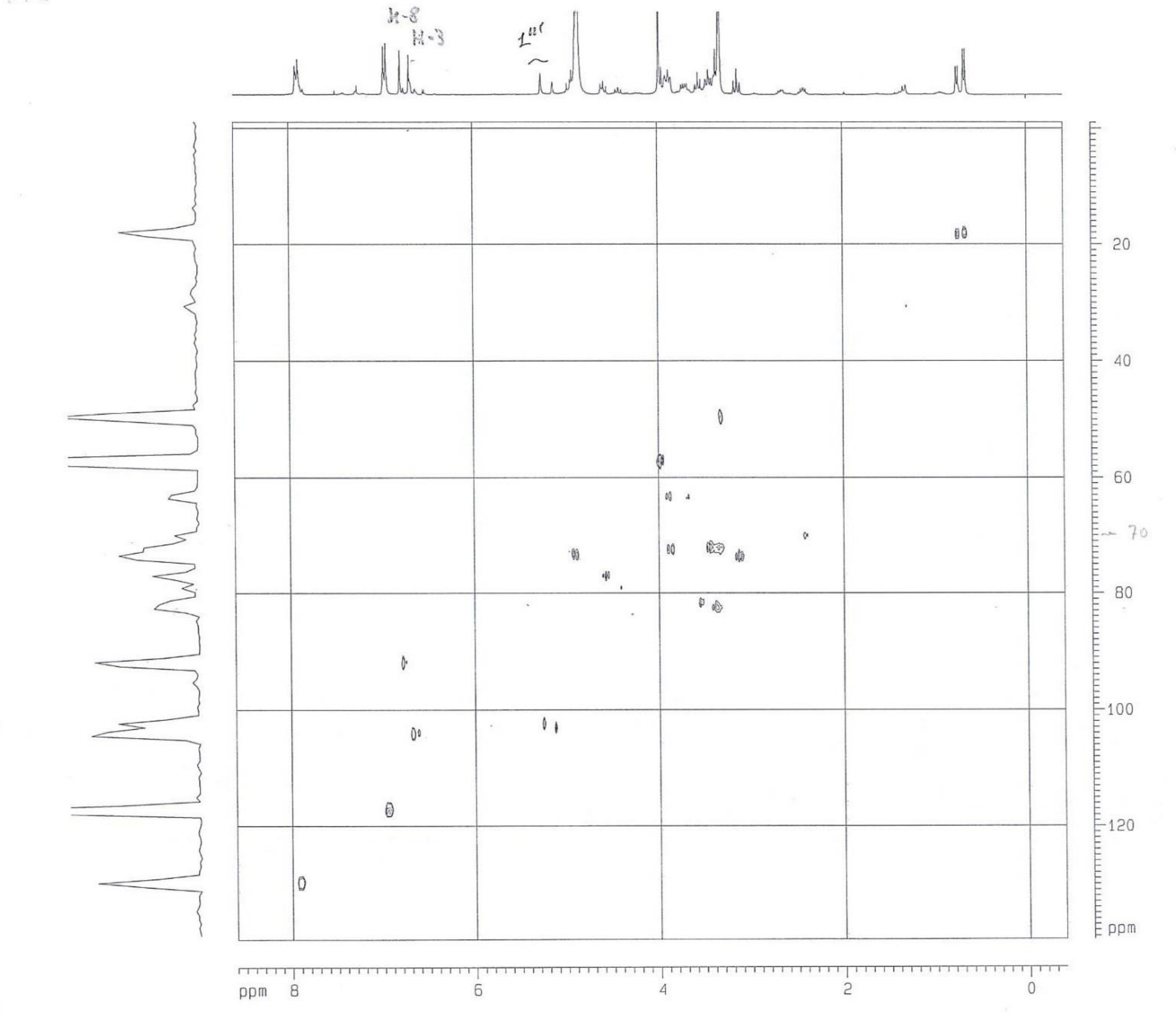

Figure S16. HSQC spectrum of 2''-*O*-α-L-rhamnosyl swertisin (**112**)

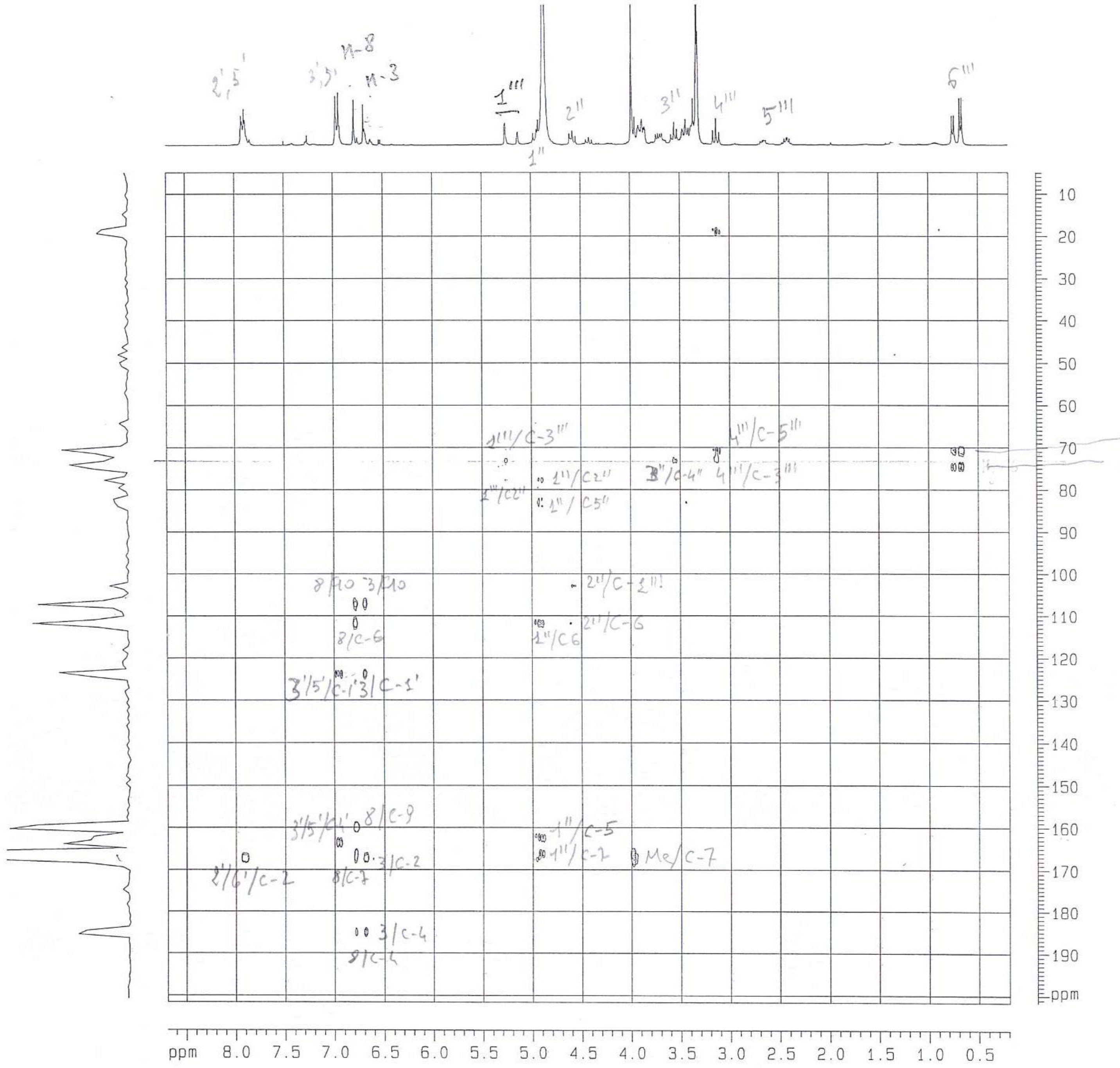

Figure S17. HMBC spectrum of 2''-*O*-α-L-rhamnosyl swertisin (**112**)

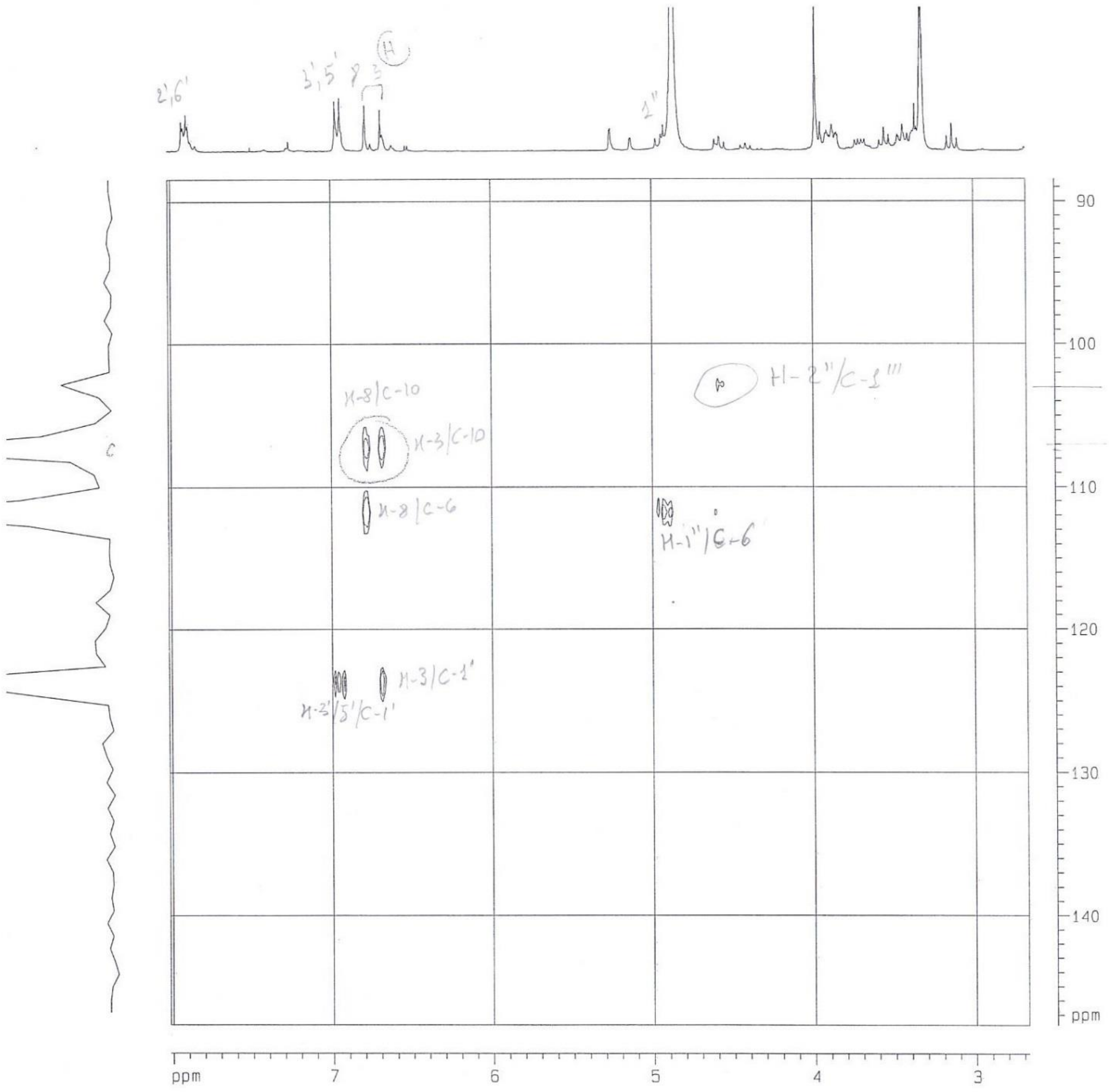

Figure S18. HMBC spectrum (enlargement 1) of 2''-*O*-α-L-rhamnosyl swertisin (**112**)

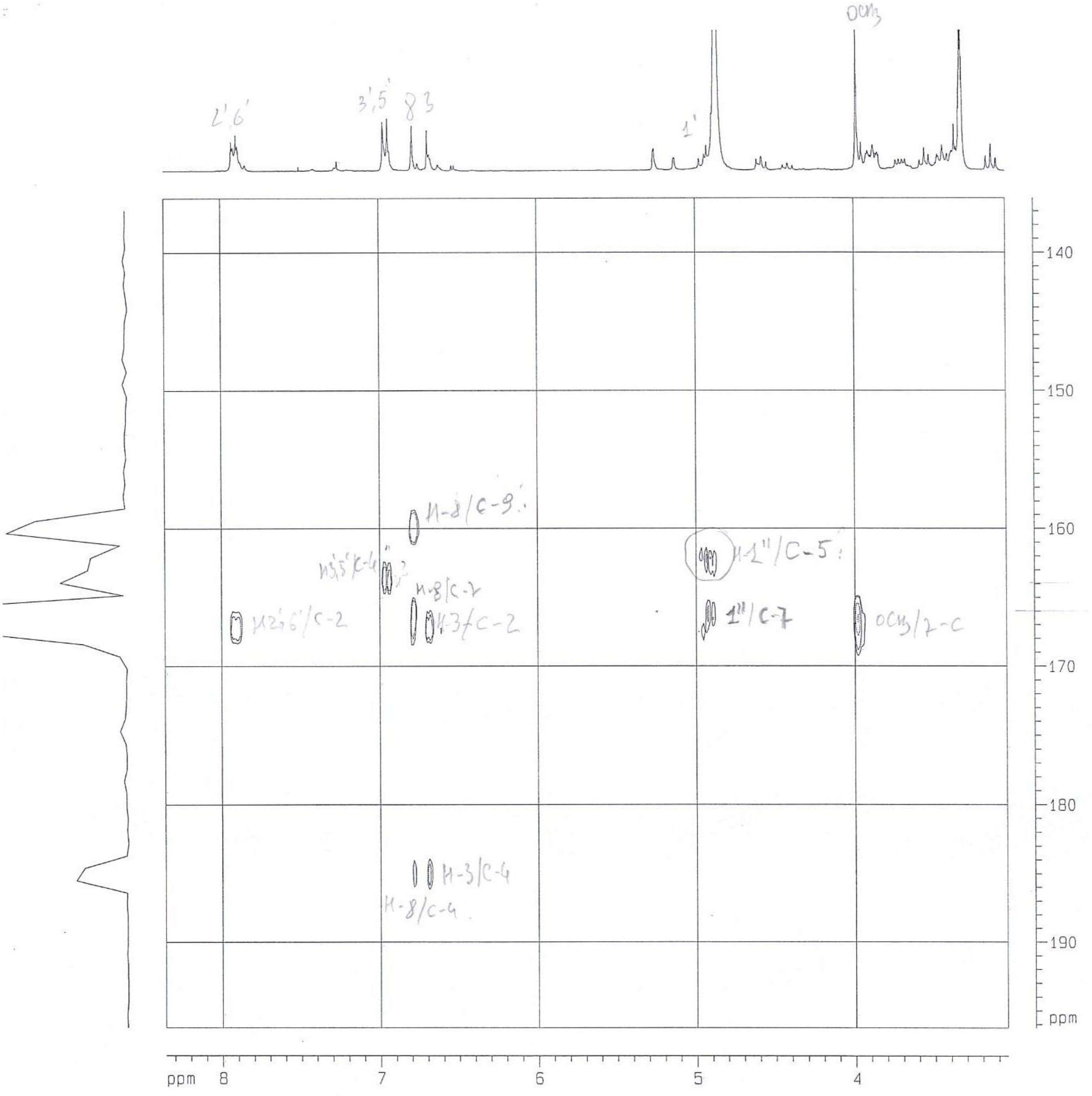

Figure S19. HMBC spectrum (enlargement 2) of 2''-*O*-α-L-rhamnosyl swertisin (**112**)

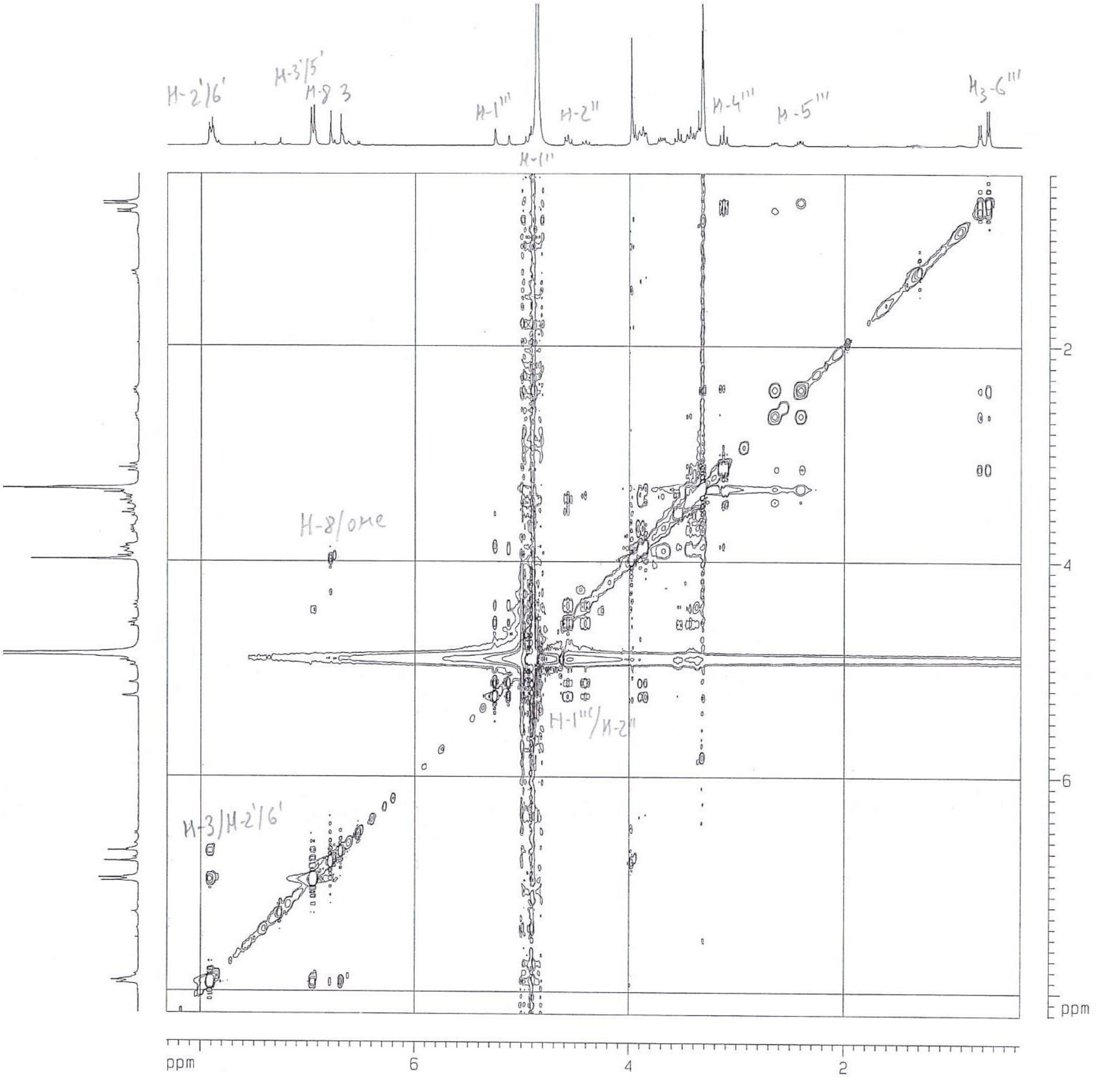

Figure S20. NOESY spectrum of 2''-*O*-α-L-rhamnosyl swertisin (**112**)

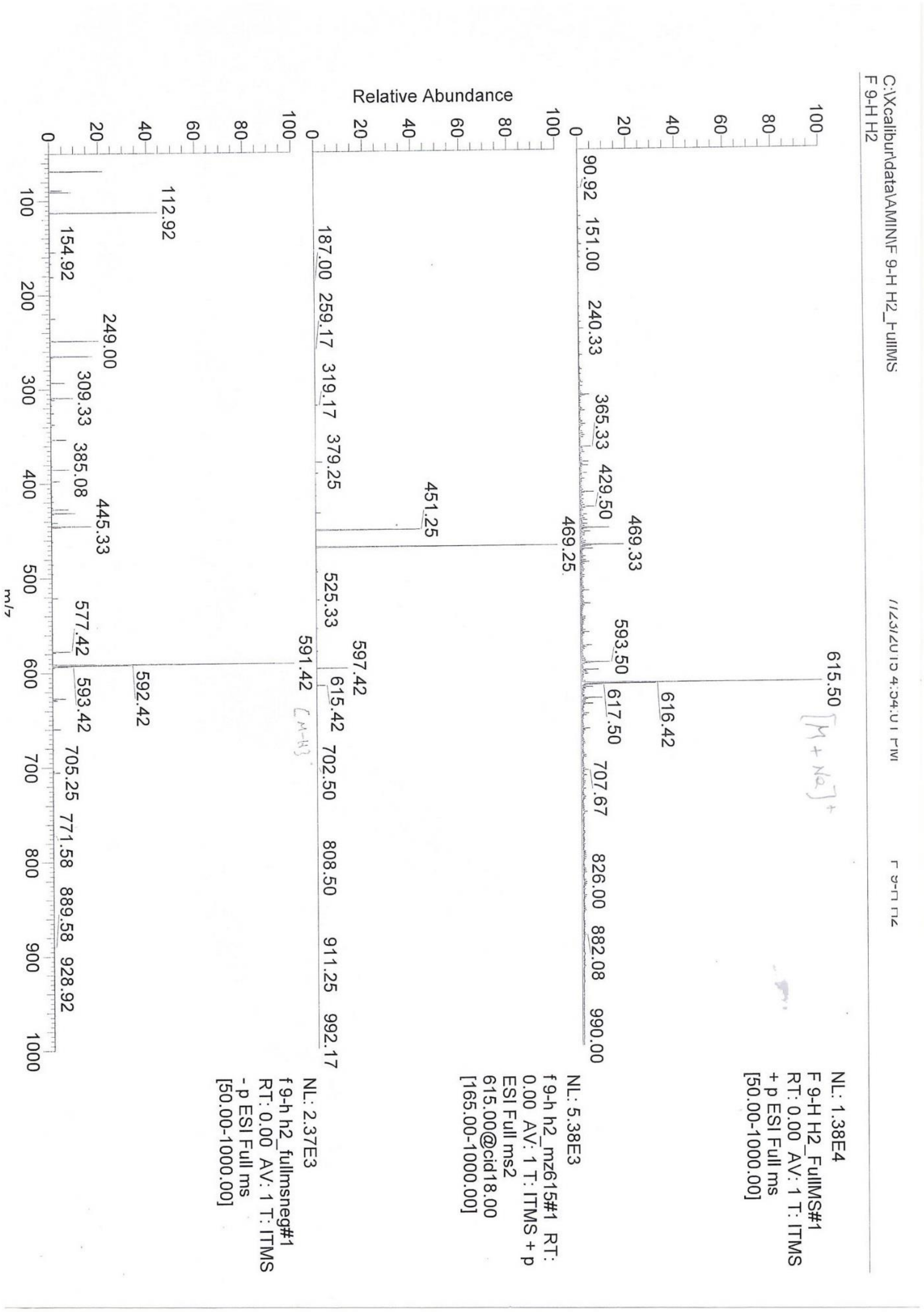

Figure S21. ESI-MS spectra (positive and negative ion mode) of 2''-*O*-α-L-rhamnosyl swertisin (**112**)

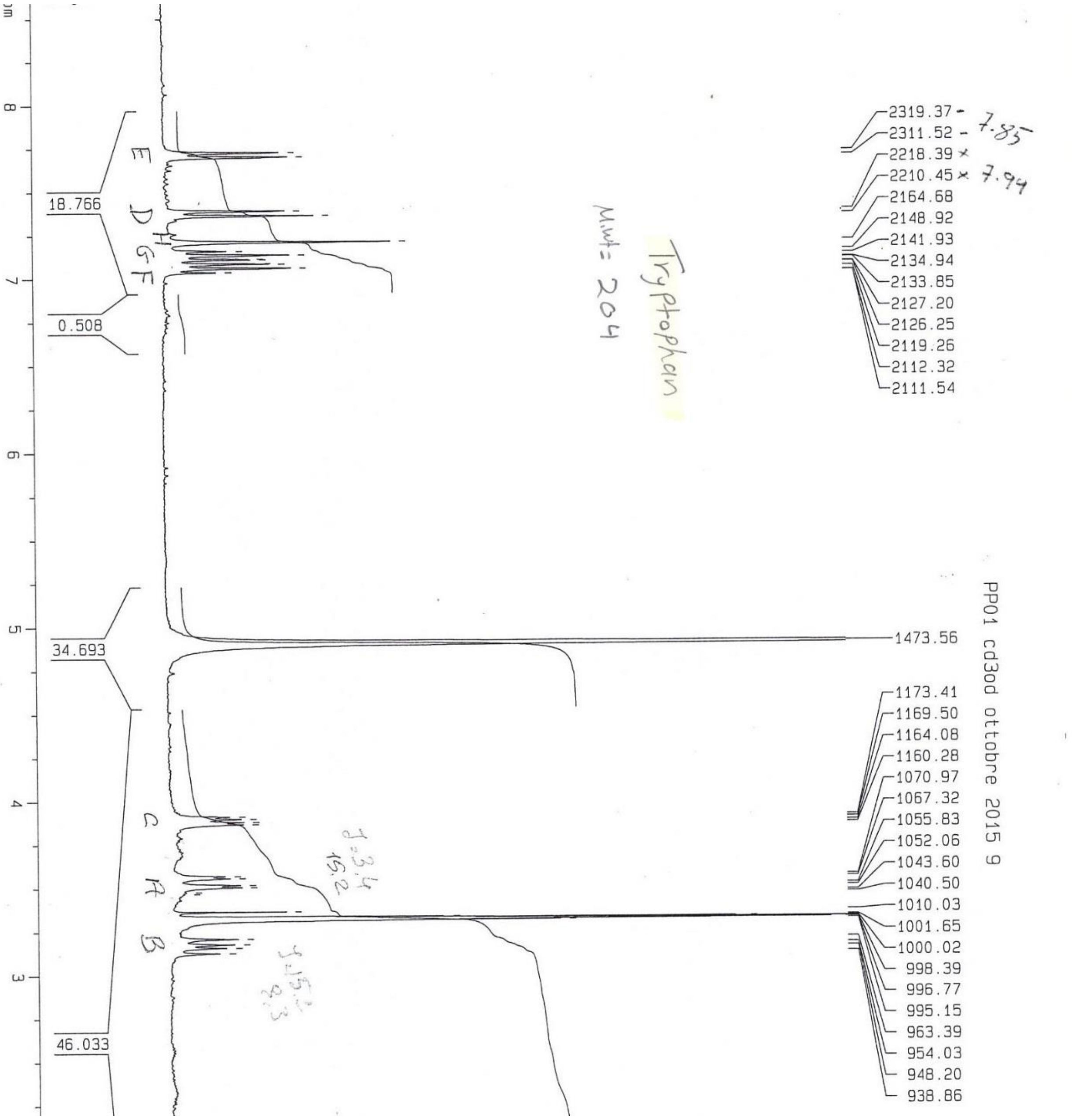

Figure S22. ^{1}H-NMR spectrum (300 MHz, CD_3OD) of tryptophan

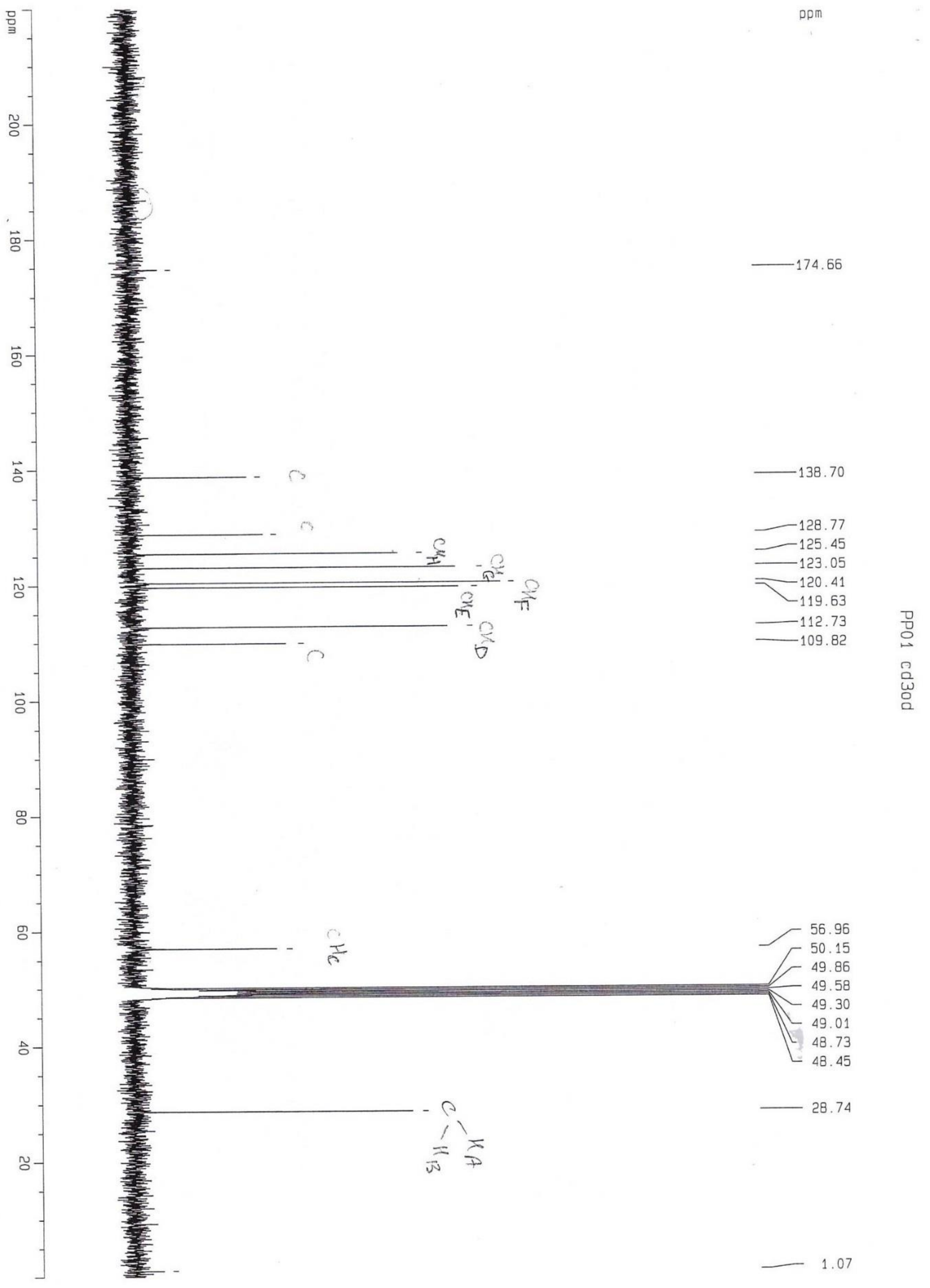

Figure S23. ^{13}C-NMR spectrum (75 MHz, CD_3OD) of tryptophan

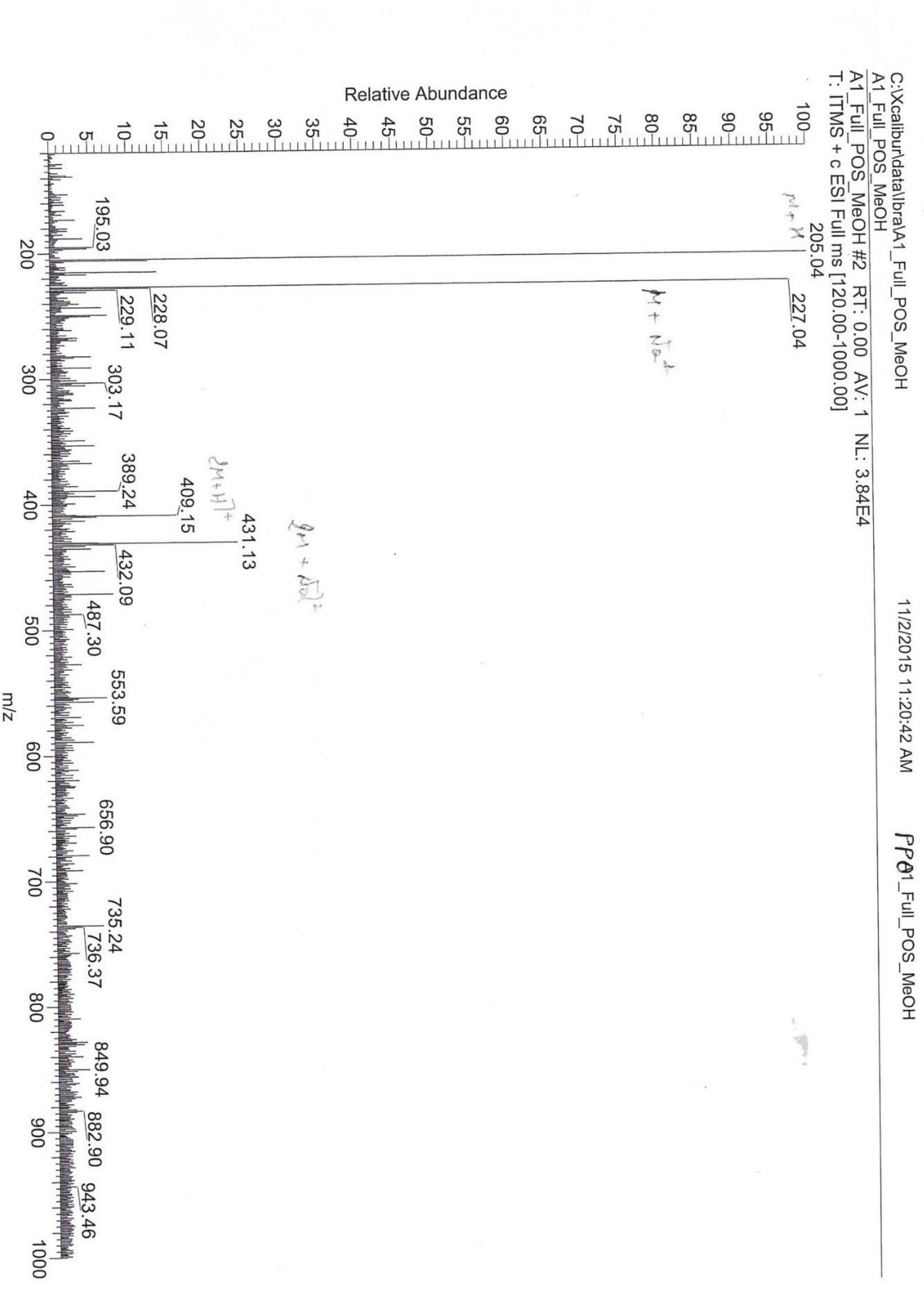

Figure S24. ESI-MS (positive ion mode) spectrum of tryptophan

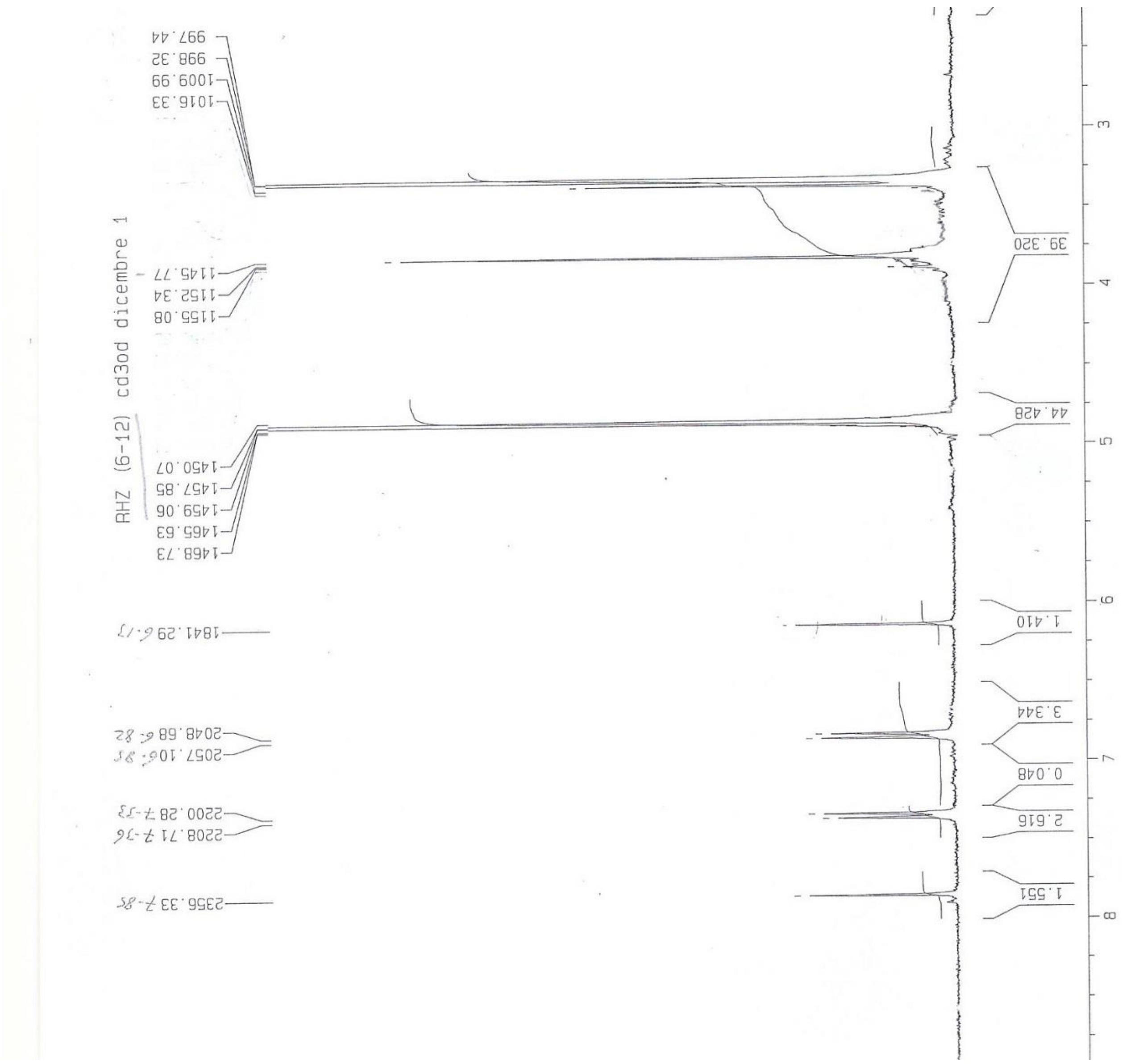

Figure S25. ^{1}H-NMR spectrum (300 MHz, CD_3OD) of isotectorigenin (**115**)

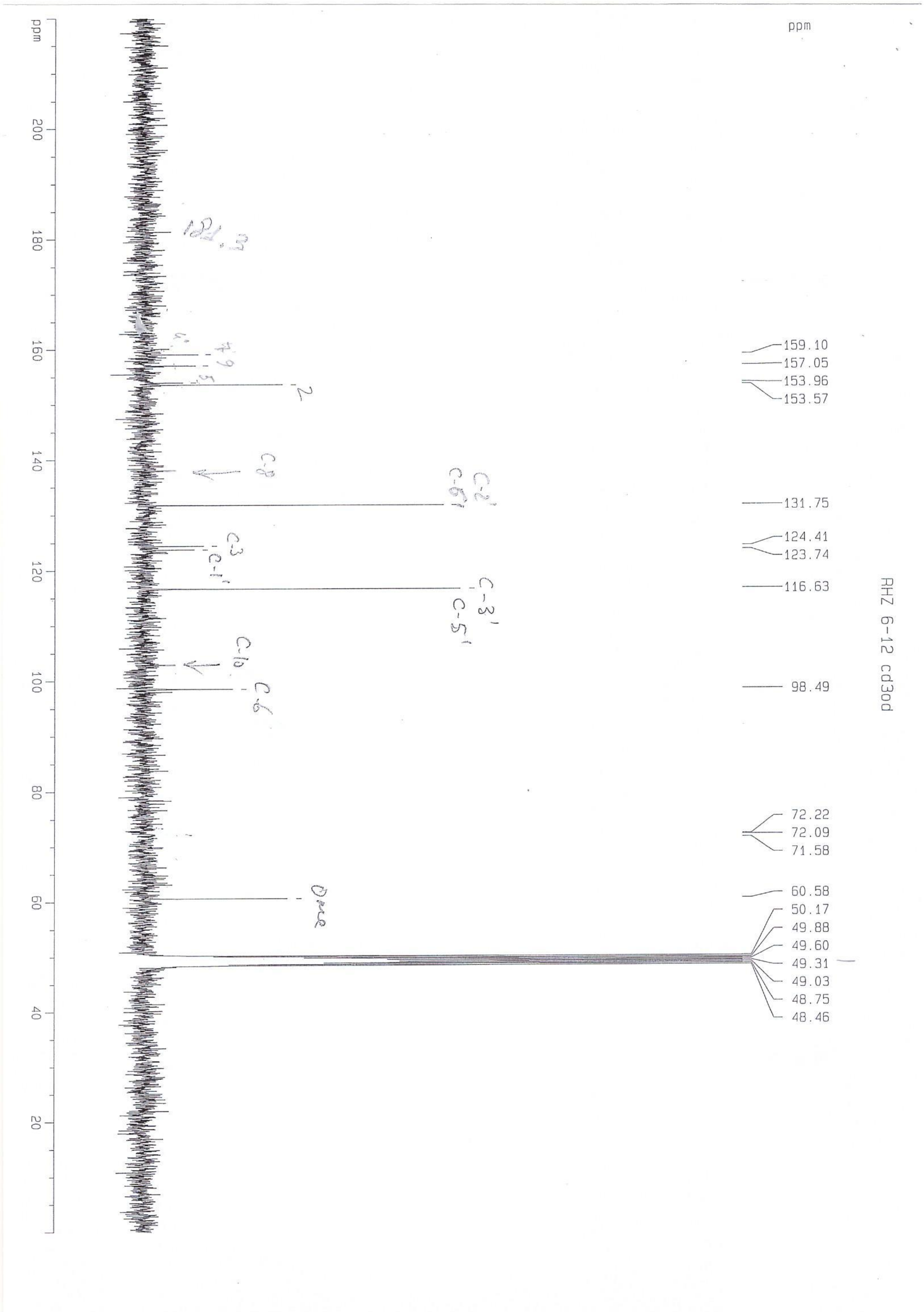

Figure S26. ^{13}C-NMR spectrum (75 MHz, CD_3OD) of isotectorigenin **(115)**

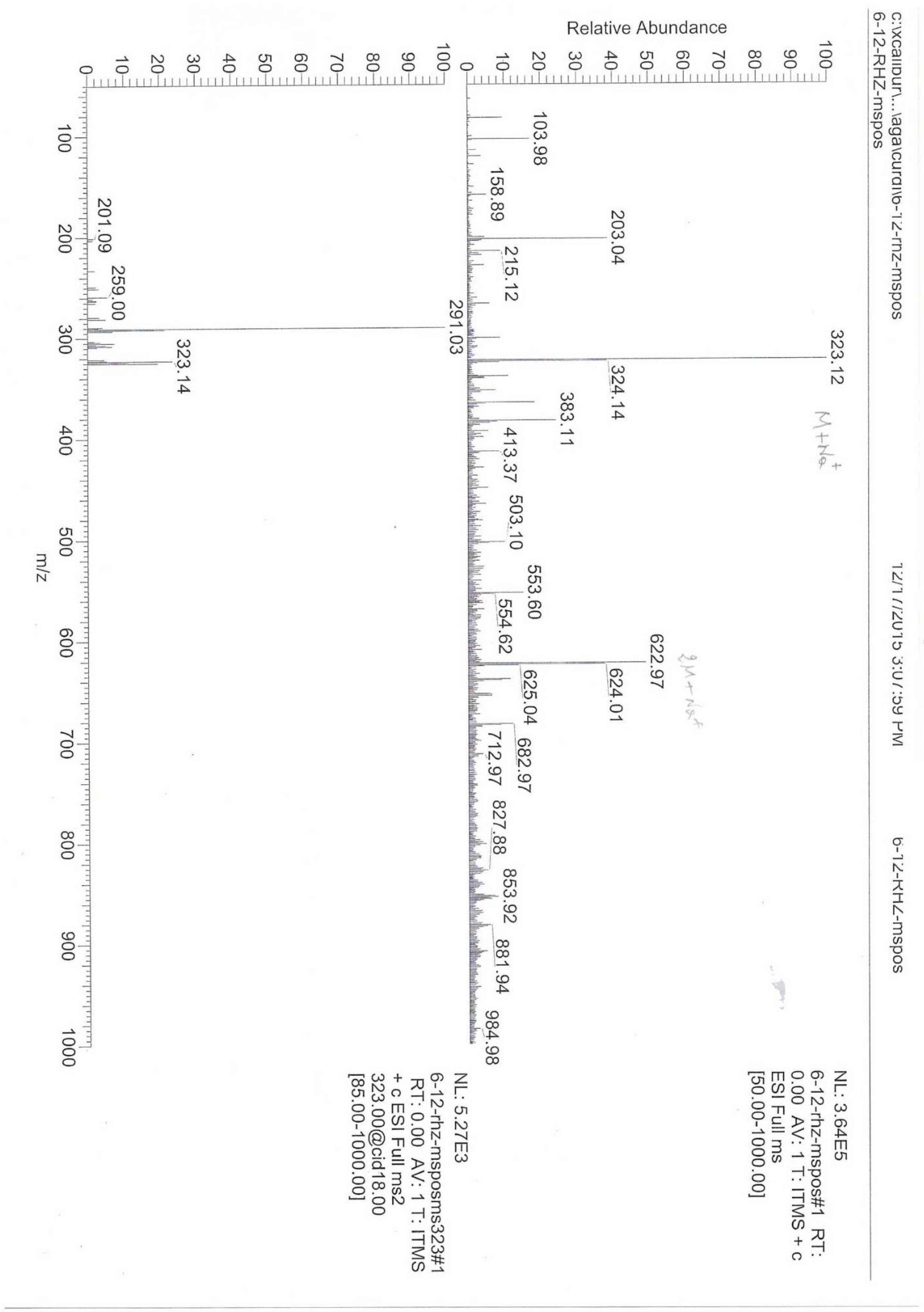

Figure S27. ESI-MS (positive ion mode) spectrum of isotectorigenin (**115**)

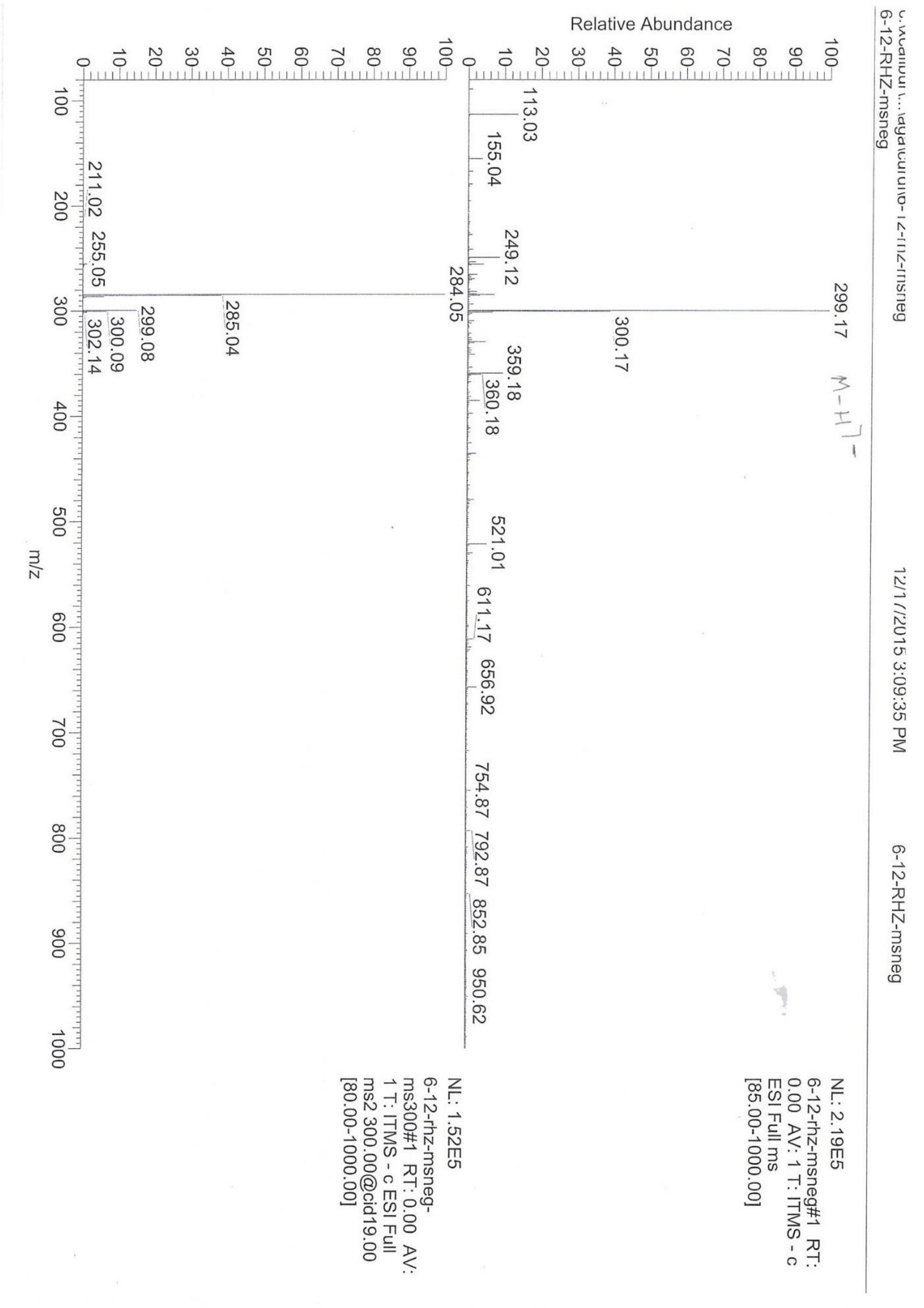

Figure S28. ESI-MS (negative ion mode) spectrum of isotectorigenin (**115**)

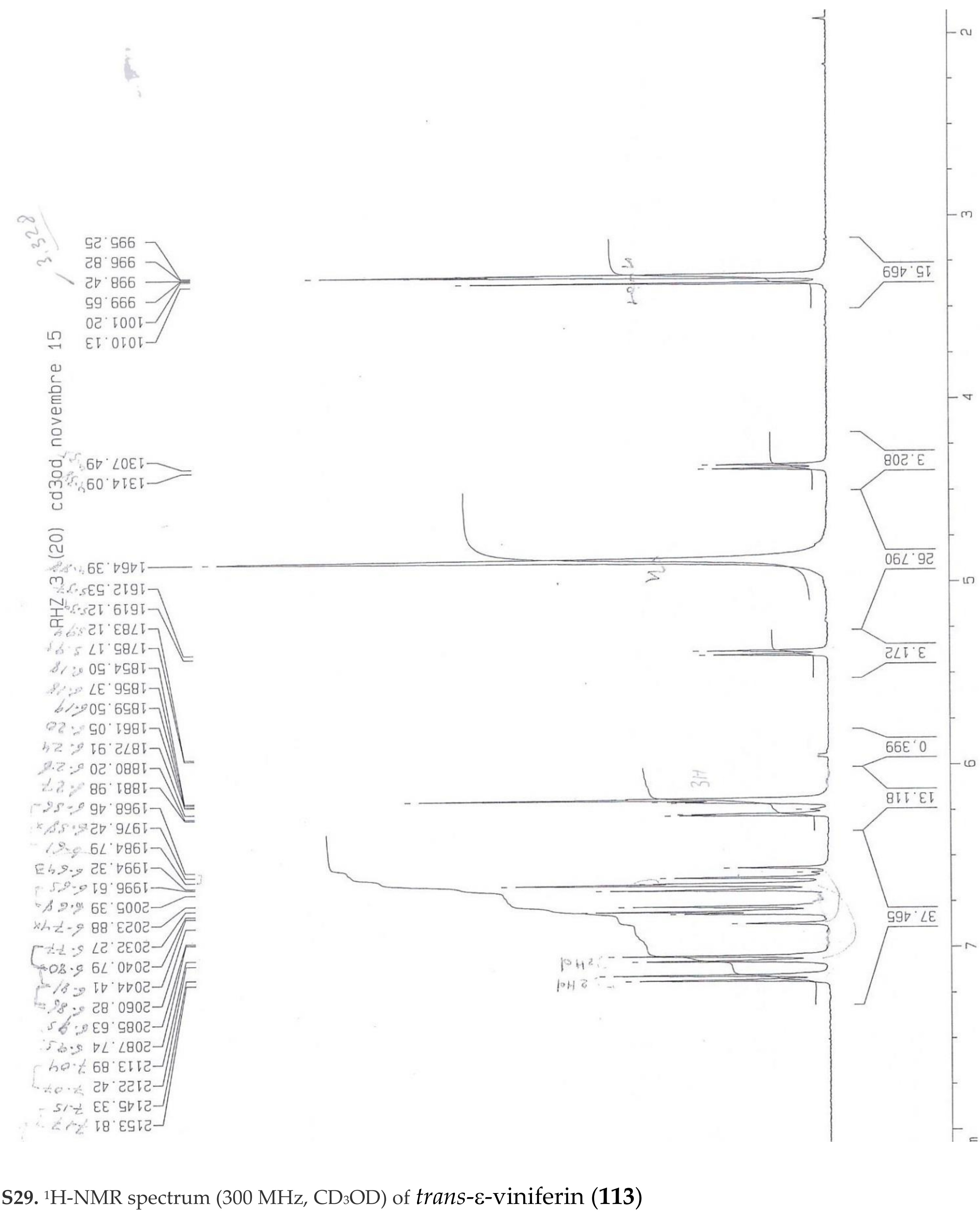

Figure S29. ^{1}H-NMR spectrum (300 MHz, CD_3OD) of *trans*-ε-viniferin (**113**)

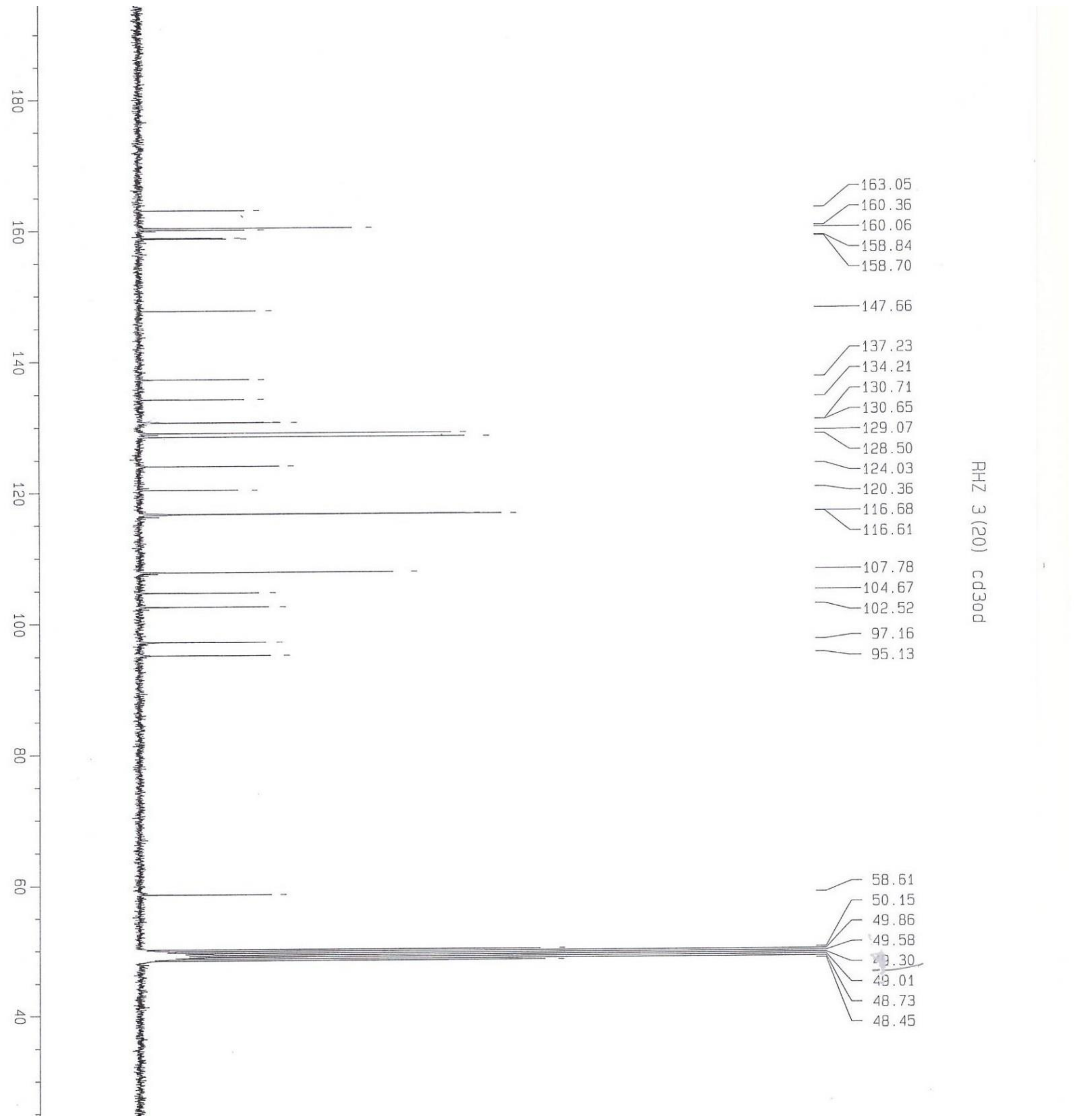

Figure S30. ^{13}C-NMR spectrum (75 MHz, CD_3OD) of *trans*-ε-viniferin (**113**)

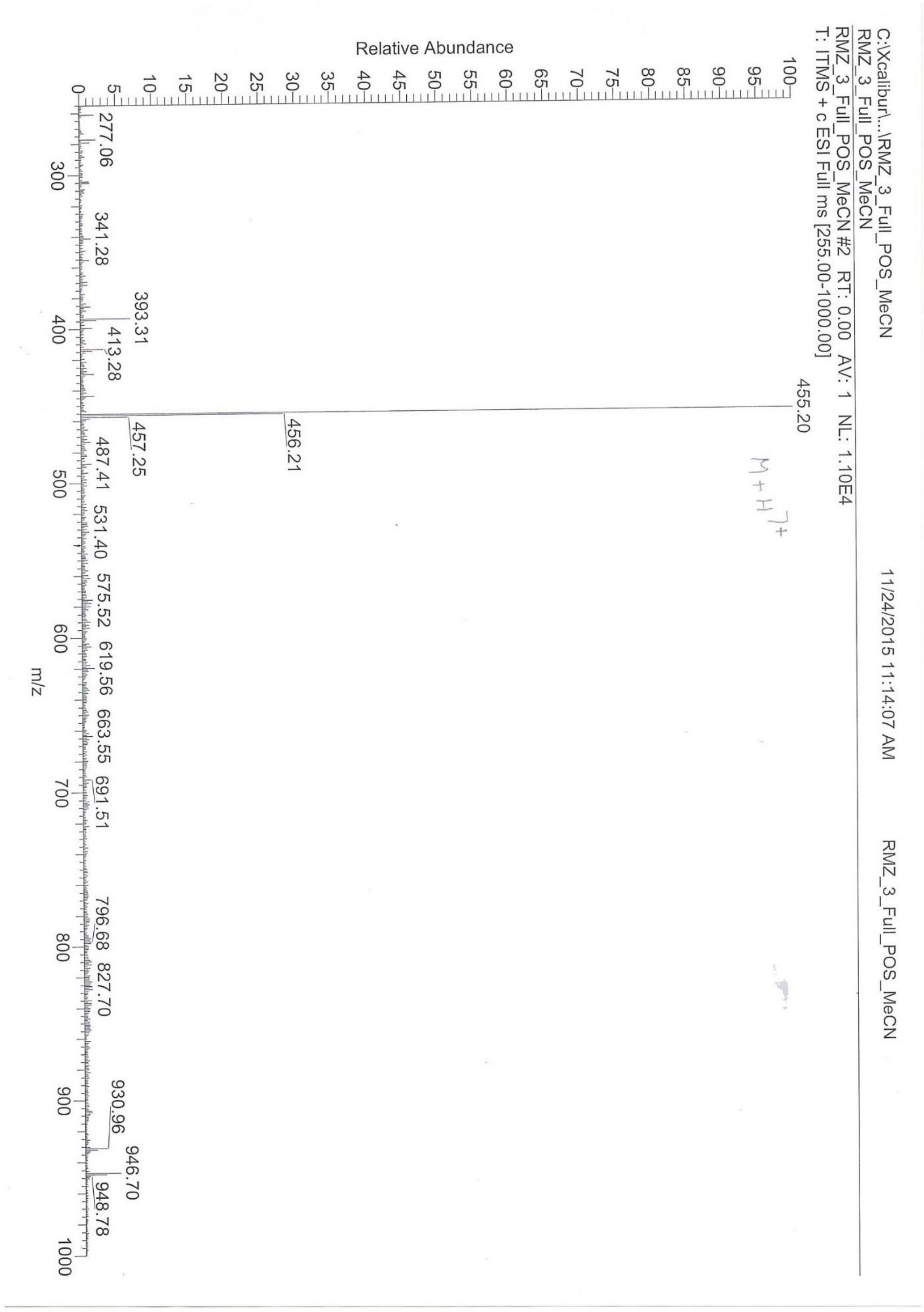

Figure S31. ESI-MS (positive ion mode) spectrum of *trans*-ε-viniferin (**113**)

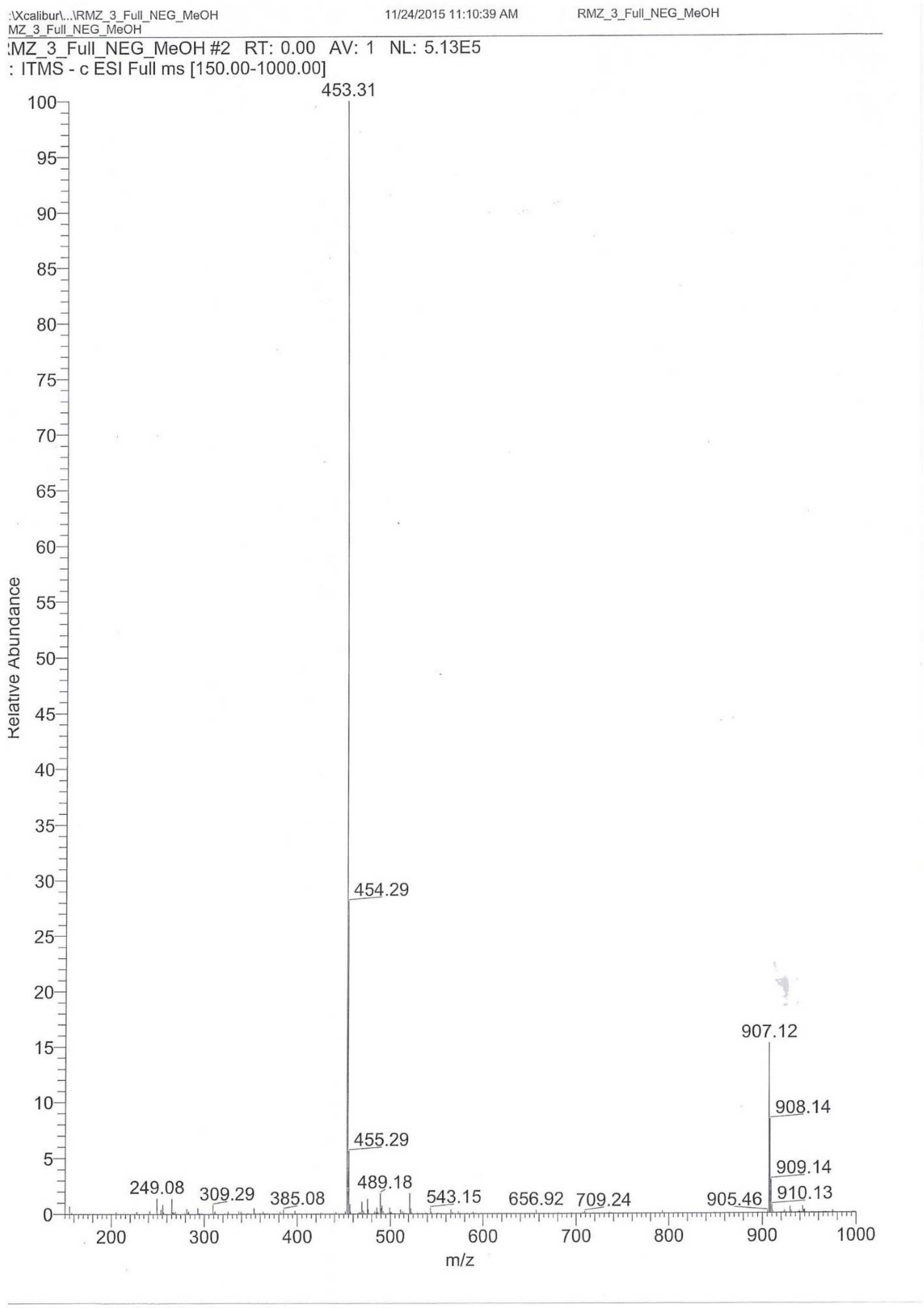

Figure S32. ESI-MS (negative ion mode) spectrum of *trans*-ε-viniferin (**113**)

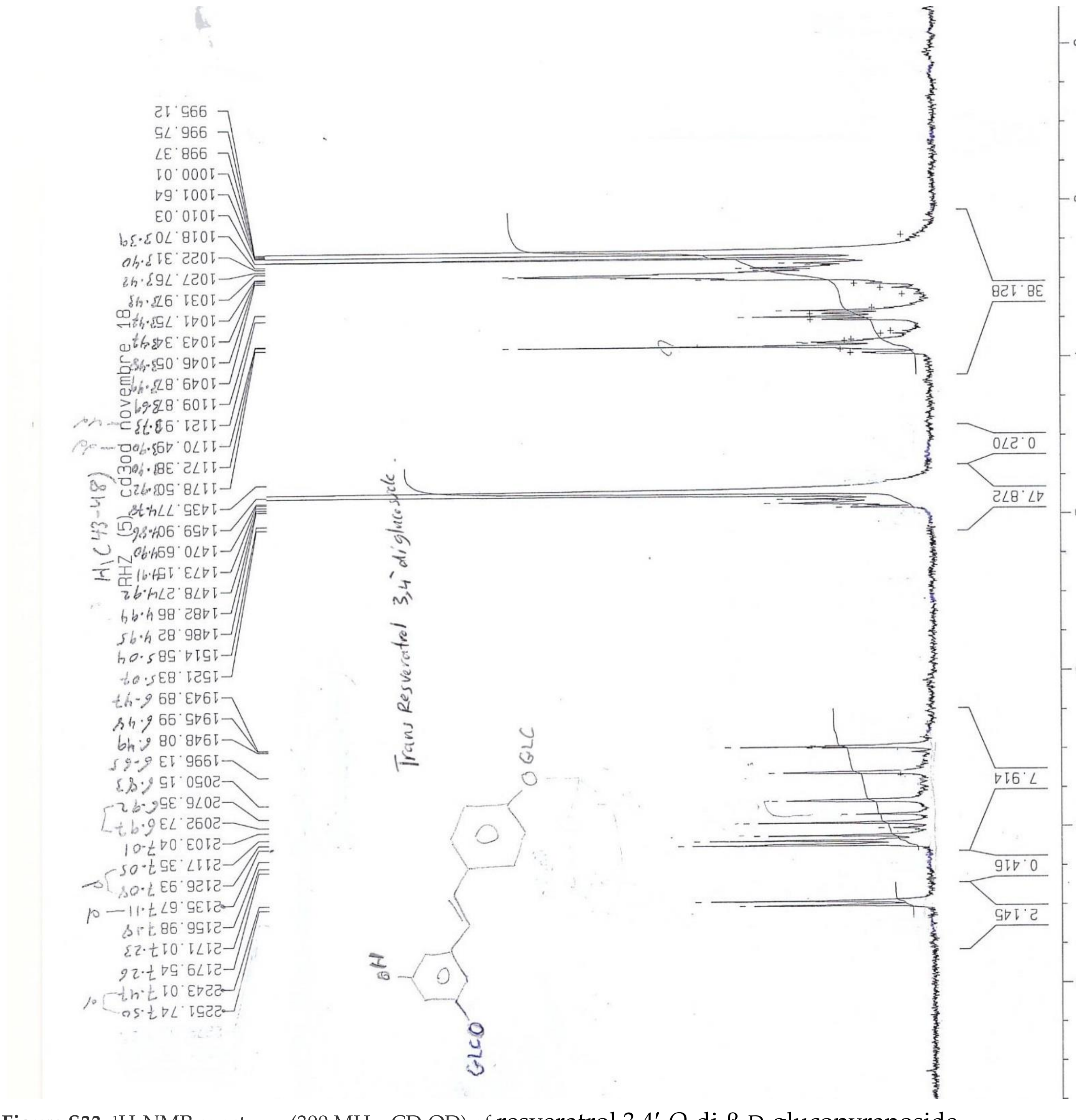

Figure S33. ^{1}H-NMR spectrum (300 MHz, CD_3OD) of resveratrol 3,4′-*O*-di-β-D-glucopyranoside (**114**)

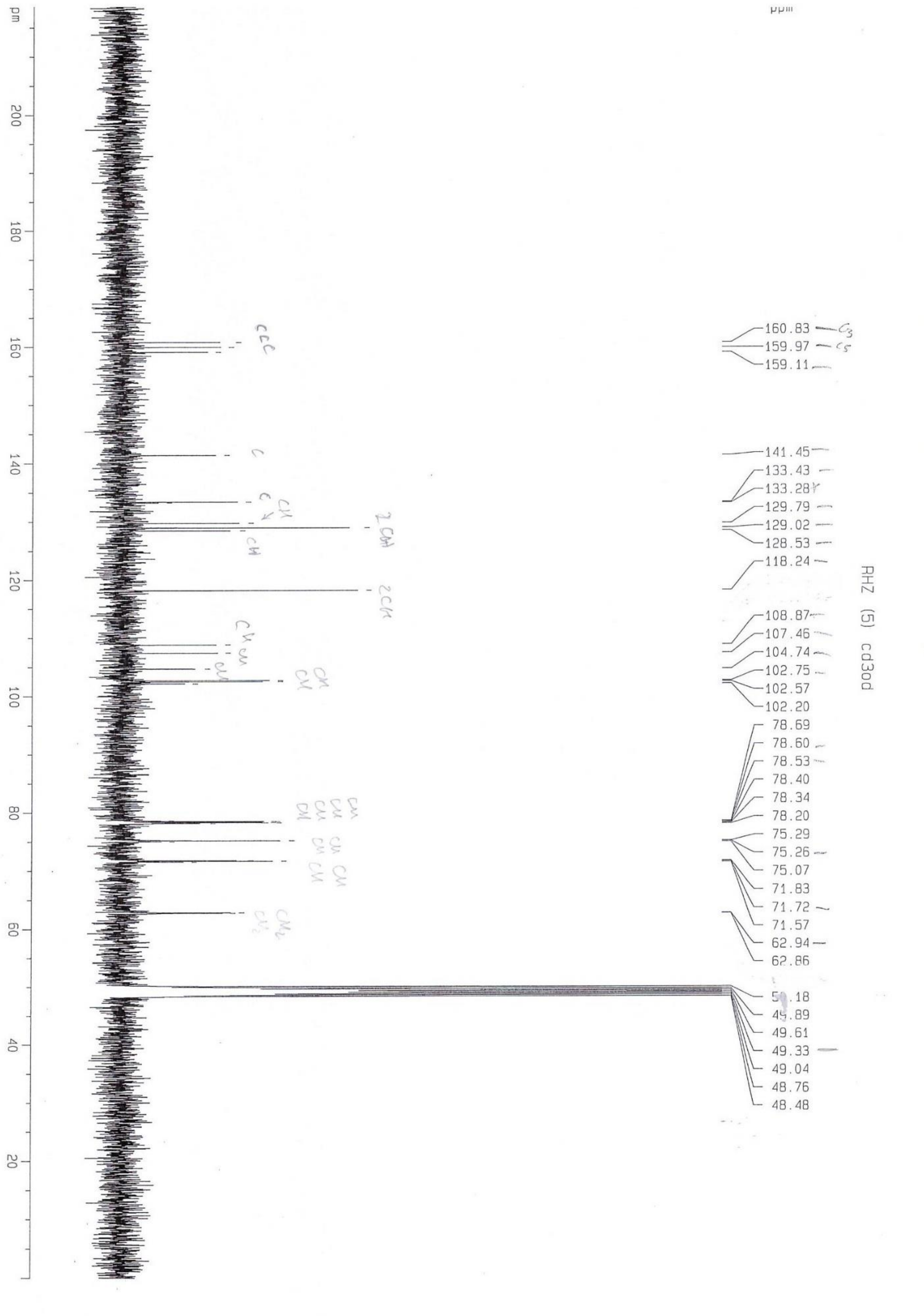

Figure S34. ^{13}C-NMR spectrum (75 MHz, CD_3OD) of resveratrol 3,4′-*O*-di-β-D-glucopyranoside (**114**)

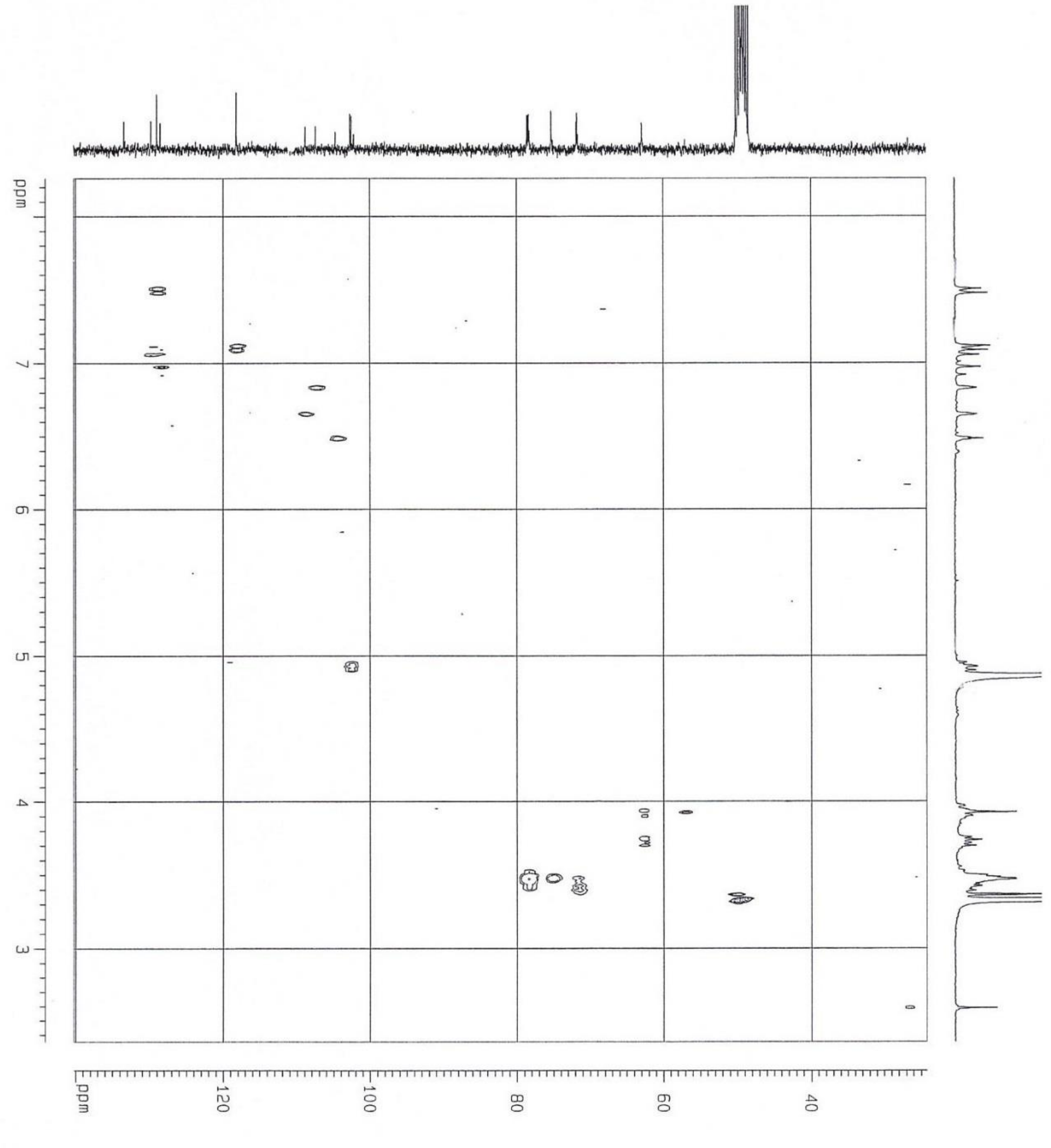

Figure S35. HSQC spectrum of resveratrol 3,4′-*O*-di-β-D-glucopyranoside (**114**)

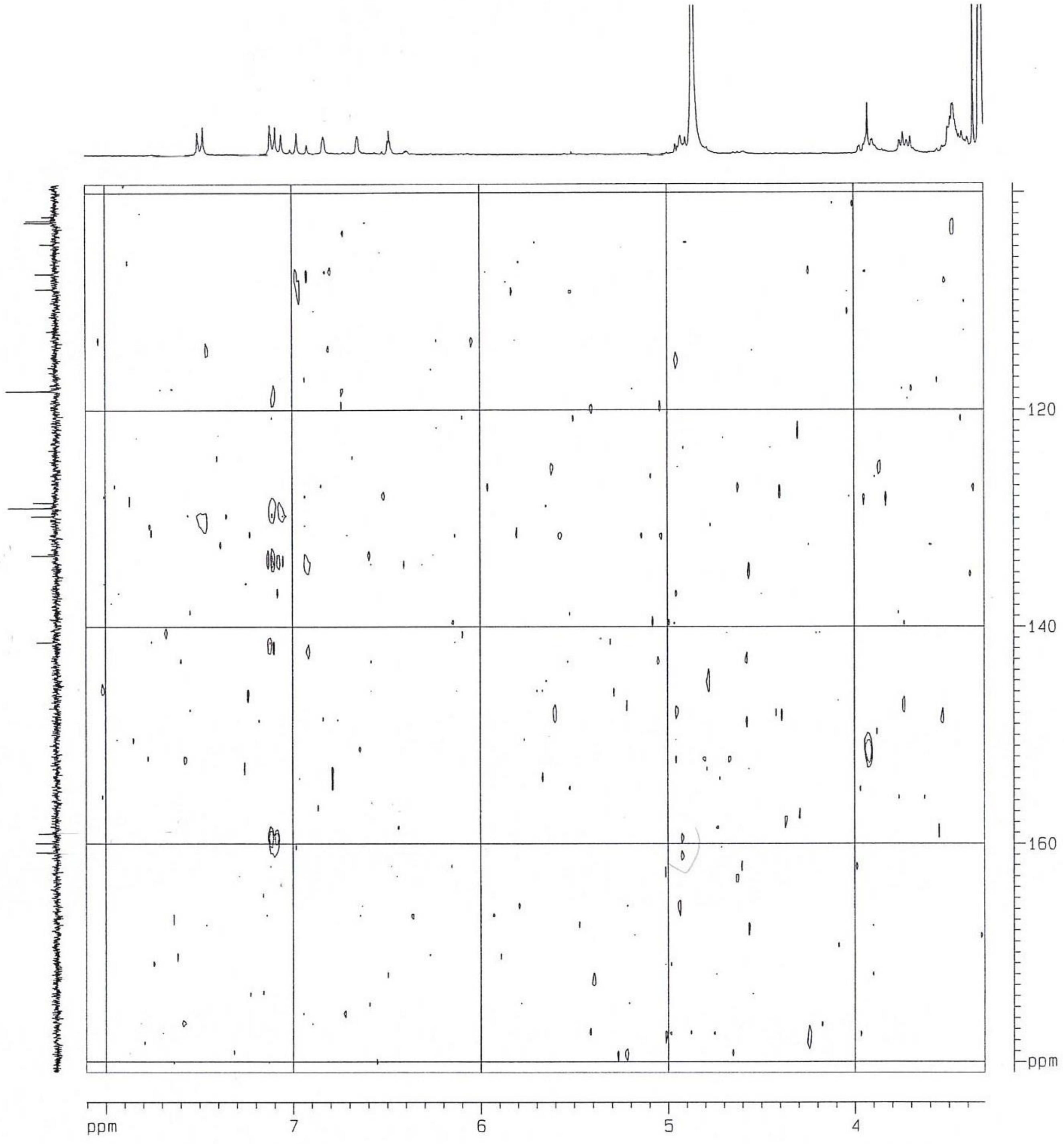

Figure S36. HMBC spectrum of resveratrol 3,4′-*O*-di-β-D-glucopyranoside (**114**)

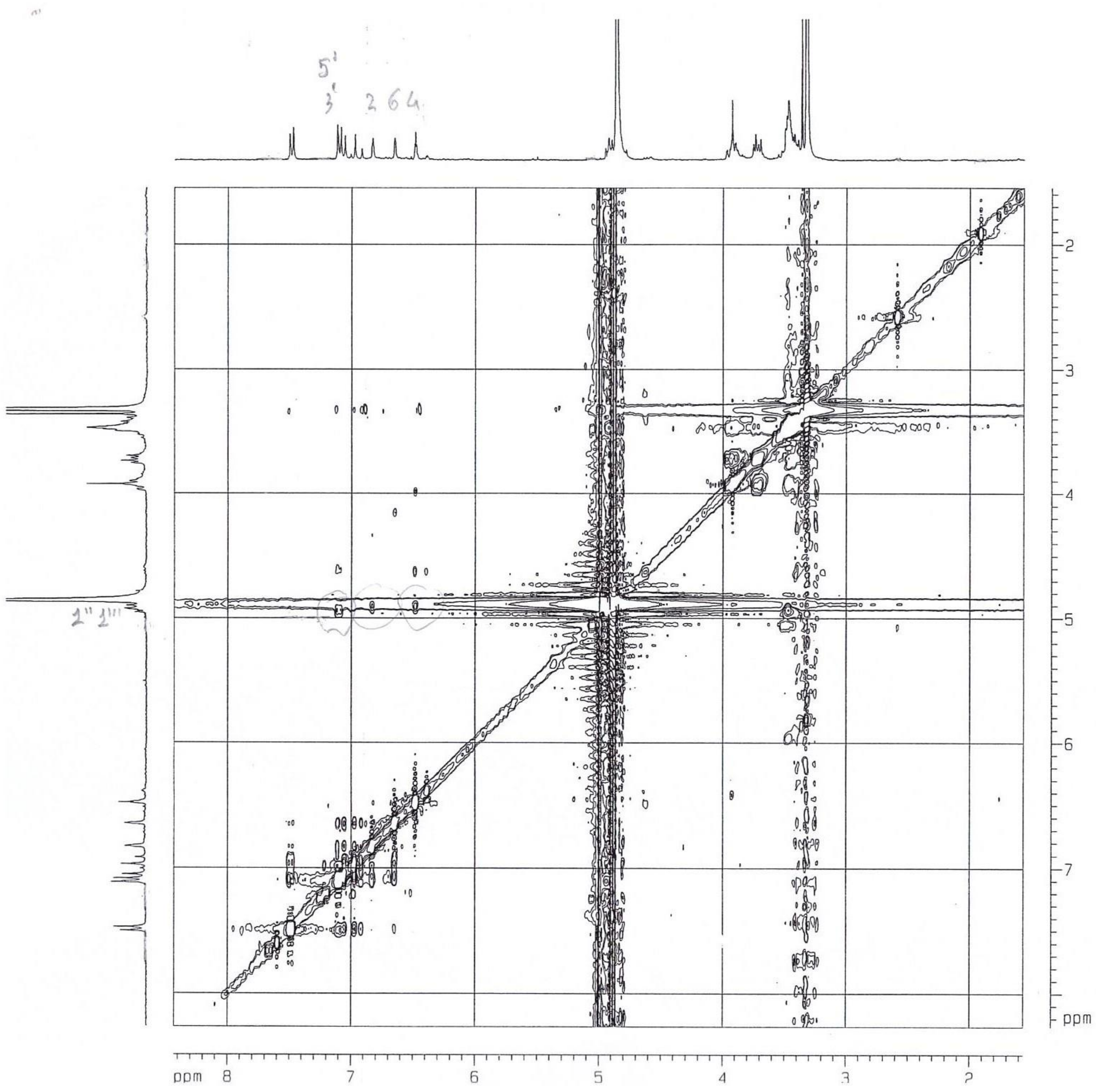

Figure S37. NOESY spectrum of resveratrol 3,4′-*O*-di-β-D-glucopyranoside (**114**)

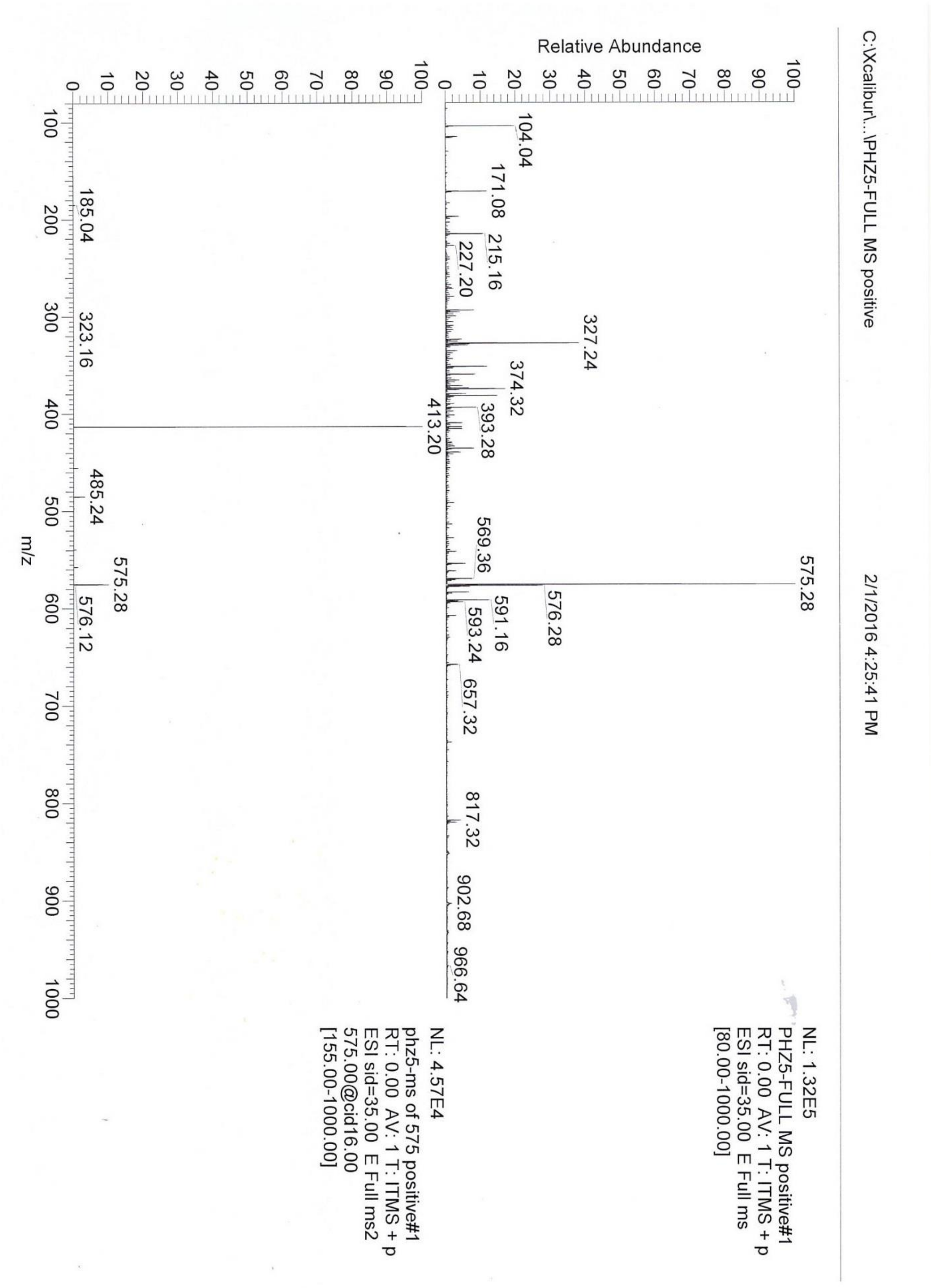

Figure S38. ESY-MS spectrum (positive ion mode) of resveratrol 3,4′-*O*-di-β-D-glucopyranoside (**114**)
